# Partial body mass regain attenuates lipid utilization and alters the hepatic lipidome linked to HDL dysfunction during metabolic syndrome in male rats

**DOI:** 10.14814/phy2.70865

**Published:** 2026-06-16

**Authors:** Eira E. Jardines, Manuel A. Cornejo, John W. Newman, Oliver Fiehn, Akira Nishiyama, Rudy M. Ortiz

**Affiliations:** ^1^ School of Natural Sciences University of California Merced California USA; ^2^ Western Human Nutrition Research Center, Agricultural Research Service United States Department of Agriculture Davis California USA; ^3^ Department of Nutrition University of California‐Davis Davis California USA; ^4^ West Coast Metabolomics Center, UC Davis Genome Center University of California Davis California USA; ^5^ Department of Pharmacology, Faculty of Medicine Kagawa University Kagawa Japan

**Keywords:** hepatic lipid metabolism, hyperinsulinemia, insulin resistance, lipid panel, MASH, MASLD

## Abstract

Increases in body mass are associated with the development of metabolic dysfunction‐associated steatotic liver disease (MASLD). Caloric restriction (CR) is the primary non‐pharmacological defense against MASLD; however, poor CR maintenance induces partial body mass regain (PR), leading to MASLD progression. We conducted an in vivo study to investigate the effects of PR following 2 weeks of CR on the hepatic lipidome during Metabolic Syndrome (MetS) using the male Otsuka Long Evans Tokushima Fatty (OLETF) rat and the Long Evans Tokushima Otsuka (LETO) rat as strain control (Ctrl). PR reduced hepatic acyl‐CoA oxidase 1 (ACOX1) and carnitine palmitoyl transferase II (CPT2) protein expression compared to OLETF CR. The lipidome indicated that PR maintained reduced total TAGs primarily MUFA and PUFA TAGs, while SFA‐TAGs were elevated. Additionally, OLETF PR demonstrated a negative correlation with cholesteryl ester and an elevation in HDL levels vs. OLETF CR. These findings suggest that PR may promote hepatic steatosis by reducing fatty acid utilization and potentially associated with dysfunctional HDL, which can induce hepatic inflammation and injury. This study highlights the detriments of PR during MetS, suggesting that careful health monitoring is necessary for individuals with compromised metabolism when using dietary interventions to ameliorate MASLD.

## 1 INTRODUCTION

Caloric restriction (CR) is the first line of behavioral intervention for metabolic dysfunction‐associated liver disease (MASLD) due to the lack of FDA‐approved treatments for MASLD. Currently, the global prevalence of MASLD is 38%, affecting 50.7% of obese individuals (Liu et al., 2022). Metabolic Syndrome (MetS) risk factors, such as hypertension and glucose intolerance, are associated with MASLD development (Chan et al., 2022), highlighting the urgency to maintain liver health. While the first FDA‐approved treatment for metabolic dysfunction‐associated steatohepatitis (MASH), resmetirom, was recently released, this new drug improves fibrosis but does not show significant improvements in MASLD activity scoring, indicating the importance of maintaining consistent behavioral interventions (Harrison, et al., 2024). Proper body mass (BM) maintenance is essential for the prevention of MASLD and the progression of MetS. Systemic hyperinsulinemia, due to insulin resistance, which is associated with glucose intolerance, can over‐activate de novo lipogenesis in the liver, causing hepatic lipid accumulation that then promotes lipotoxicity and MASH due to the formation of triglyceride (TAG) derived metabolites (e.g., diacylglycerols (DAG), ceramides, and cholesterols) and reactive oxygen species (ROS) (Vacca et al., 2015). Non‐adipose tissue, especially the liver, accounts for approximately 50% of fatty acid re‐esterification (intracellular recycling) into TAG after overnight fasting and is increased in diabetic conditions (Reshef, 2003).

Under MetS conditions, there is an imbalance between lipid utilization and availability, which induces hepatic fat accumulation and the development of MASLD (Vázquez‐Jiménez et al., 2023). In hepatocytes cultured in FA‐rich media, the downregulation of acyl‐CoA oxidase 1 (ACOX1) increased TAG accumulation and decreased β‐hydroxybutyrate (β‐HB), a clear sign of blunted β‐oxidation suggesting that changes in ACOX1 reciprocally alter hepatic TAG accumulation, which are halted by FA availability (Wang et al., 2019). Major consequences of excess hepatic fat accumulation include hepatic fibrosis, lipotoxicity, and increased oxidative stress (Jia et al., 2021).

The effects of insulin resistance, T2DM, and MetS on the plasma lipidome have been broadly studied. For example, in T2DM patients, non‐esterified fatty acids (NEFA), especially monounsaturated fatty acids (MUFA), were positively correlated with fasting glucose, while monoacylglycerols (MAG) were inversely correlated with BMI (Grapov et al., 2012). However, a robust assessment of the hepatic lipidome during insulin‐resistant conditions associated with significant oscillations in BM (adiposity) is lacking and should be of particular importance due to its relation to non‐alcoholic fatty liver disease (NAFLD, now MASLD) and MASH (Sanyal et al., 2001).

A previous study in Otsuka Long Evans Tokushima Fatty (OLETF) rats, a model of MetS that closely resembles human conditions, observed that 12 weeks of moderate CR (30%) decreased hepatic steatosis, TAG accumulation, and de novo lipogenesis, and most of these changes were partially reversed after 4 weeks of ad libitum refeeding, highlighting the importance of preserving energy balance to prevent MASLD (Linden et al., 2016). The same degree of CR from 4 to 40 weeks of age prevented the appearance of hepatic steatosis in OLETF rats, although a compensatory upregulation of lipogenesis accompanied this change (Rector et al., 2011), suggesting that moderate CR suffices to attenuate hepatic steatosis. Although these studies demonstrated the benefits of CR, the detriments of body mass regain, hepatic lipid metabolism as a whole, and hepatic lipidomic analysis were not assessed. These measurements give insights into different lipid species and their determinants, which are often missed during studies. Thus, an assessment of the effects of acute (10 days) and severe (50%) CR, and the subsequent PR, on the hepatic lipidome during insulin resistance is lacking. An acute and severe CR would allow us to potentially mimic the type of diet obese individuals with rapid weight loss expectations may attempt. Several studies using short‐term, severe (low‐calorie diets) CR have been performed on individuals with obesity to reduce body mass in clinical trials, with these studies indicating that obesity is a chronic condition requiring continuous caloric maintenance (Atkinson, 1993; Haywood et al., 2018; Thomas, 2000). Participants (*n* = 14) with obesity in a rapid weight loss competition lost 4.3 lbs/weeks, which is double to quadruple the CDC's 1 to 2 lbs/weeks recommendation, but regained 75% of the lost mass within 6 years associated with reduced metabolic function, revealing the detriments of regain (Fothergill et al., 2016). Additionally, the regain of 1.5 kg or more in males was sufficient to induce the recurrence of MASLD indicating that lesser increases in BM regain are enough to impair hepatic health once compromised (Nakanishi et al., 2021).

Many refeeding studies have focused on solely one part of lipid metabolism and miss the bigger picture. Furthermore, a study focusing on the change of the hepatic lipidome after body mass has yet to be conducted. Therefore, this paper aims to demonstrate the detriment of body mass regain after caloric restriction on hepatic lipid metabolism and hepatic lipidome during MetS. The data demonstrated that PR disrupts fatty acid utilization induced during CR, which prevents hepatic lipid accumulation by downregulating ACOX1 and carnitine palmitoyltransferase II (CPT2). Additionally, we found PR was positively correlated with lysophosphatidylcholine (LPC), while negatively correlated with cholesterol ester (CE) and an elevation in plasma HDL levels, which are associated with HDL dysfunction. Collectively, this data revealed that 1 week of improper caloric maintenance via ad lib refeeding is sufficient to abolish the benefits of CR.

## 2 METHODS

The current study focuses on hepatic lipid metabolism and complements previously published data from the sample set (Cornejo et al., 2020). All experimental procedures were reviewed and approved by the institutional animal care and use committee of Kagawa Medical University (Kagawa, Japan).

### 2.1 Caloric restriction (CR) and partial regain (PR)

Briefly, the Long‐Evens Tokushima Otsuka (LETO) strain control (*n* = 29) and obese, insulin‐resistant OLETF rats (n = 29) were fed ad libitum with standard laboratory rat chow (MF; Oriental Yeast Corp., Tokyo, Japan) for 4 weeks. At 15 weeks of age, male rats of each strain were separated into ad libitum fed control groups (*n* = 8/group/strain), CR groups (*n* = 7/group/strain), and partial BM regain (PR) groups (n = 7/group/strain) (Figure S1). Both CR and PR groups were subjected to 50% CR (compared to their respective ad libitum controls) for 10 days. CR groups were subjected to an oral glucose tolerance test (oGTT) and dissected 3 days later along with their respective ad libitum controls (CR Ctrl, *n* = 8/group/strain). Meanwhile, PR groups were fed ad libitum for 7 days, achieving partial body mass regain (73% regain of mass loss) before an oGTT and dissection, paired with their PR ad libitum control groups (PR Ctrl, *n* = 7/group/strain). Both CR and PR control groups were fed ad libitum for the entire study. The animal groups were as follows: (1) LETO CR ad libitum fed control (LETO CR Ctrl; *n* = 8), (2) LETO CR (*n* = 7), (3) LETO PR ad libitum fed control (LETO PR Ctrl; n = 8), (4) LETO PR (n = 7), (5) OLETF CR ad libitum fed control (OLETF CR Ctrl; n = 8), (6) OLETF CR (n = 7), (7) OLETF PR ad libitum fed control (OLETF PR Ctrl; n = 8), and (8) OLETF PR (n = 7). However, they were considered independent groups to avoid the confounding factor of age as they were dissected 1 week apart; however, no significant differences in physiological measurements were detected between CR and PR control groups despite the slight age difference, suggesting that age was not a confounding factor here. Animals were maintained in a specific pathogen‐free facility under controlled temperature (23°C) and humidity (55%) with a 12‐h light, 12‐h dark cycle. All animals were given free access to water for the entire study.

### 2.2 Oral glucose tolerance test (oGTT)

Oral glucose tolerance tests (oGTT) were performed as previously reported (Cornejo et al., 2020) and insulin resistance index (IRI) was calculated as previously described (Rodriguez et al., 2012) and reported (Cornejo et al., 2020). Briefly, before dissections, a 2 g/kg glucose bolus was administrated using an oral gavage to overnight fasted rats where blood was collected via caudal vein before gavage and 15, 30, 60, and 120 min after. While these data have been previously reported, they were used here to correlate the shifts in lipid classification with glucose area under the curve (AUC), insulin AUC, and IRI.

### 2.3 Tissue collection

After an overnight fast, animals were anesthetized with pentobarbital (100 mg/kg i.p.) and perfused systemically with chilled PBS via the abdominal aorta with an incision in the inferior vena cava as the exit point and proceeded until no blood was present. Thereafter, several tissues, including liver, were rapidly removed, weighed, snap frozen in liquid nitrogen, and kept at −80°C until analyzed. Aliquots of liver (*n* = 5 per group/strain, 10 ± 1 mg wet mass) were analyzed for lipidomics by charged surface hybrid column (CSH) electrospray ionization (ESI) quadrupole time‐of‐flight (QTOF) mass spectrometry (MS) data acquisition as previously described (Fiehn, 2016), generating a dataset of 553 consistently identified lipids.

### 2.4 Western blots

Homogenates of liver were separated into cytosolic and plasma membrane fractions. For proteins located in the nuclear fraction, cytosol and nuclear extractions were conducted as previously described (Dimauro et al., 2012; Godoy‐Lugo et al., 2022). Fraction purity was assessed by measuring fraction‐specific proteins (α‐tubulin for cytosol, Na^+^‐K^+^ATPase for membrane, and H3 for nuclear). Supernatant total protein content was measured by Bradford assay (ThermoFischer Scientific, Bothell, Washington, 23238) and Pierce BCA assay (Thermo Scientific, Bothell, Washington, PI23224 and PI23228). Western blots were performed as we previously reported (Godoy‐Lugo et al., 2022). Details of primary antibodies are listed in Table 1. The secondary antibodies (LI‐COR Biosciences, Lincoln, USA) were diluted 1:20,000 in TBS with 0.2% Tween‐20 and 0.1% SDS. Membranes were rewashed and visualized using a Li‐Cor Odyssey Imaging System. Densitometry values were quantified by ImageJ software (NIH) and further normalized by correcting for densitometry values of a representative protein band stained with Ponceau S (except for KAT3B, which was normalized by total protein content). Normalization using Ponceu S was done by dividing the band intensity of the protein of interest, normalized using LETO CR Ctrl, by the densitometry values of the Ponceu S bands.

**TABLE 1:** Antibodies used for western blots.

Antibody name	Catalog number	Company	Dilution	Total protein	Fractions
Na+/K+ ATPase	3010	Cell Signaling	1:40,000	5–20 μg	Membrane and Cytosol
FATP5	390,917	Invitrogen	1:1000	40 μg	Membrane
CD36	PA1‐16813	Invitrogen	1:1000	40 μg	Membrane
α‐tubulin	3873	Cell Signaling	1:40,000	5–20 μg	Membrane and Cytosol
B‐Actin	3700S	Cell Signaling	1:1000	5–20 μg	Cytosol and Nuclear
CPT1A	15,184‐1‐AP	Proteintech	1:2000	15 μg	Cytosol
Acox1	10,957‐1‐AP	Proteintech	1:4000	25 μg	Cytosol
CPT2	PA5‐109586	Invitrogen	1:500	35 μg	Cytosol
GPAM	AB6990	Abcam	1:500	35 μg	Cytosol
DGAT1	PA5‐79150	Invitrogen	1:500	20 μg	Cytosol
FAS	3189S	Cell Signaling	1:500	35 μg	Cytosol
H3	9715S	Cell Signaling	1:3000	5–25 μg	Cytosol and Nuclear
KAT3B	AB246522	Abcam	1:500	20 μg	Nuclear

### 2.5 Biochemical analysis

Liver total protein content was measured by Bradford assay (ThermoFischer Scientific, Bothell, Washington, 23238) and Pierce BCA assay (Thermo Scientific, Bothell, Washington, PI23224 and PI23228). Plasma clinical lipid profiles, including HDL, LDL, and total cholesterol, were measured by the Clinical Pathology Lab (UC Davis).

### 2.6 Lipidomics data acquisitions and processing

Hepatic, nontargeted lipidomic analyses were conducted by charged surface hybrid column‐electrospray quadruple time of flight mass spectrometer tandem mass spectrometry (CSH‐ESI QTOF MS/MS) at the West Coast Metabolomics Center as previously described (Bishop et al., 2023). Briefly, 3 μL of hepatic resuspended nonpolar phases were injected into a ThermoFisher Scientific Vanquish quadrupole time of flight mass spectrometer (UHPLC) + liquid chromatography systems coupled to a Q‐Exactive HF orbital ion trap mass spectrometer. The column compartment and mobile phase preheater were set at 65°C and with a 0.6 mL/min mobile phase flow rate.

The raw data was processed by Agilent's MassHunter Qual software to detect peaks up to 300 chromatograms. The peak features were then imported into MassProfiler Professional for peak alignments to determine the peaks that are present in multiple chromatograms. After which, the peaks are then transferred into a MassHunter quantification method using MS/MS information and LipidBlast library to identify lipids.

### 2.7 Data analysis and statistics

Data were presented as means (±SE). Group differences were assessed using a three‐way ANOVA when normalization was met, and Kruskal–Wallis when violated for lipidomics data. Multiple paired differences within groups for each combination of lipids, chain length, or saturation were individually assessed using Friedman with Dunn's to correct for multiple comparisons. Pairwise comparisons were performed using the Wilcoxon matched‐pairs signed rank test or Mann–Whitney test, which was dependent on whether the data were paired or unpaired. All tests used LETO CR Ctrl as the reference to determine relative changes. Significance (*p* < 0.05) is considered only for age‐paired groups, except for CR versus PR phases. Outliers were excluded by the extreme studentize deviate test with an *α* = 0.05. Results are reported as a percentage of expression compared to LETO CR Ctrl mean or fold‐change.

### 2.8 Data normalization and transformation

Lipidomic samples were run in a single batch in a random injection order, with one quality control (human plasma), pool sample (rat liver), and blank for every 10 samples. Data were reported as quantitative ion peak heights and the Systematic Error Removal Using Random Forest (SERRF) algorithm (Fan et al., 2019) was run on raw data (without pools or blanks) to compare the quality control relative standard deviation (QC RSD) of different normalization methods, using the SERRF algorithm code in R (https://raw.githubusercontent.com/slfan2013/SERRF‐online/master/backup%20js/normalization.R). Transformations were performed using rank transformation, depending on data normality (Dhillon et al., 2018). The Man‐Whitney test was performed in 10 previously selected pairwise comparisons (Table S1), and Benjamini‐Hochberg False Discovery Rate (FDR) correction *q*‐values (Benjamini & Hochberg, 1995) were calculated for each metabolite per comparison.

### 2.9 Principal component analysis (PCA)

A single principal component analysis (PCA) was run for all groups. Each comparison was graphed using the principal components that best demonstrated the separation between the groups, which was determined using a one‐way ANOVA. For visualization purposes only, the two groups are shown in each figure. For each comparison, 10% of the highest positive and negative loadings with *p*‐values <0.05 are shown in Table 2 and all loadings are available for each PCA in Tables S3–S7.

**TABLE 2:** Lipids with the top 5 positive and negative loadings (by absolute value) for PC1.

Principal component 1, Comparison I		
Lipid	PC1 score	*Z*‐scores
PC 38:5 A (2)	0.94452	1.233549549
PC 40:6 A (2)	0.94009	1.224130117
PC 38:3 A	0.93565	1.214670536
PC 38:5 B (2)	0.92569	1.193475514
PC 36:5 C	0.92078	1.183021062
PC 35:3	0.91984	1.181017616
PC 37:5	0.91196	1.164248168
PC 36:5 D	0.91135	1.162943637
PC 35:2 B	0.90855	1.156995783
PC 34:3 B	0.90789	1.155583388
LPC o‐16:0	−0.60564	−2.06588218
LPC p‐16:0 or LPC o‐16:1	−0.62092	−2.09839197
SM d34:0	−0.62317	−2.10318567
LPE 16:1e	−0.64322	−2.14585877
LPE 18:1e (2)	−0.67031	−2.20352447
LPE 18:1e	−0.67357	−2.21044974
Ceramide d34:2	−0.69672	−2.25972773
Cholesterol	−0.75059	−2.37439971
PE 40:7 B	−0.7703	−2.41634573
PE 35:0 PE 17:0–18:0	−0.77515	−2.42666021

Principal component 2, Comparisons: A, C, E		
Lipid	PC2 score	*Z*‐scores
Ceramide d39:1	0.89947	2.065670552
Cer‐NS d41:2 Cer‐NS d26:2/15:0	0.89765	2.06117398
Ceramide d42:2 B	0.88485	2.029625362
Cer‐NS d42:3 Cer‐NS d18:2/24:1	0.87852	2.013999609
Cer‐NS d41:2 Cer‐NS d18:2/23:0	0.85973	1.967678879
Cer‐NS d42:3 Cer‐NS d18:1/24:2	0.85559	1.957476168
Ceramide d43:1	0.85309	1.95130615
Cer d42:2 B	0.8451	1.931615193
Ceramide d41:1	0.8421	1.924200799
SM d39:1 (2)	0.82161	1.873685094
TAG 56:3 TAG 18:1–18:1–20:1	−0.87886	−2.31919958
TAG 50:5	−0.8791	−2.31979686
TAG 53:3 TAG 17:1–17:1–19:1	−0.88024	−2.32260018
TAG 52:7 TAG 16:1–16:1–20:5	−0.88268	−2.32860119
TAG 51:3	−0.8845	−2.33310383
TAG 54:6	−0.88542	−2.33536109
TAG 54:3 TAG 16:0–18:1–20:2	−0.8878	−2.34123649
TAG 52:5 TAG 16:0–16:0–20:5	−0.89198	−2.35153323
TAG 50:4 TAG 16:1–16:1–18:2	−0.89425	−2.35713408
DG 34:3	−0.89496	−2.35888945

Principal Component 3, Comparisons: B, D, H		
Lipid	PC3 Score	*Z*‐scores
FA 20:3 (eicosatrienoic acid)	0.93483	2.230706377
FA 20:2	0.92239	2.191657886
FA 22:3	0.9196	2.182910295
FA 18:1 (oleic acid)	0.90592	2.13998005
FA 18:2 (linoleic acid)	0.89731	2.1129834
FA 15:0 (pentadecylic acid)	0.8911	2.093491655
FA 16:0 (palmitic acid)	0.88845	2.085168726
LPE 19:0	0.88202	2.065001661
FA 18:1	0.8759	2.045789109
FA 15:1 B	0.87259	2.035408292
PC 33:2	−0.38152	−1.89938855
PC p‐36:2 or PC o‐36:3	−0.39862	−1.95302888
PC 35:4	−0.41215	−1.9954977
LNAPS 38:5 LNAPS 18:0/n‐20:5	−0.43115	−2.055115
LNAPS 38:4 LNAPS 17:2/n‐21:2	−0.44155	−2.08772384
LNAPS 36:2 LNAPS 17:2/n‐19:0	−0.44335	−2.09337539
LNAPS 40:5 LNAPS 18:0/n‐22:5	−0.44569	−2.10071504
LNAPS 36:4 LNAPS 16:0/n‐20:4	−0.45889	−2.14212927
LNAPS 40:6 LNAPS 18:0/n‐22:6	−0.47031	−2.17797266
LNAPS 38:6	−0.54261	−2.40480675

Principal component 4, Comparisons: F & J		
Lipid	PC4 score	*Z*‐scores
PC p‐36:3 or PC o‐36:4	0.77509	2.590523
PE p‐38:4 or PE o‐38:5	0.72211	2.402941
PC p‐38:4 or PC o‐38:5 A	0.72082	2.398368
PC p‐38:4 or PC o‐38:5 B	0.70855	2.354952
PE p‐36:4 or PE o‐36:5	0.70111	2.328583
PC p‐38:4/PC o‐38:5 B	0.70083	2.327617
PC p‐36:3 or PC o‐36:4 2	0.6942	2.304134
PE 40:4 PE 18:0–22:4	0.69374	2.3025
PC p‐36:4/PC o‐36:5	0.69094	2.292583
PC 32:0	0.68902	2.285797
PC 32:1	−0.43517	−1.6945
PE 36:5 A	−0.44193	−1.71845
PE 40:6 A	−0.44722	−1.73717
PC 35:2 A	−0.45512	−1.76516
PC 38:7	−0.46962	−1.8165
PC 38:6 C	−0.47144	−1.82294
PC 40:7 A	−0.47952	−1.85155
PC 36:3 B	−0.48197	−1.86023
PI 40:7	−0.50415	−1.93875
PE 38:7 PE 16:1–22:6	−0.52506	−2.01279

Principal component 5, Comparisons: G & H		
Lipid	PC5 score	*Z*‐scores
PE p‐36:1 or PE o‐36:2	0.66824	2.952599311
SM d36:1 (2)	0.66819	2.952364515
PE p‐40:6 or PE o‐40:7	0.61744	2.712161806
SM d38:1 (2)	0.61215	2.687134771
DAG 36:0 DAG 18:0–18:0	0.54907	2.388584333
PE p‐38:3 or PE o‐38:4	0.54477	2.368254189
Ceramide d38:1	0.54294	2.359570181
Ceramide d36:1	0.54113	2.351017451
Cer d36:1	0.52658	2.282140425
SM d36:2 (2)	0.50658	2.187465769
PC 36:4 C	−0.36933	−1.95821381
PE 38:5 B	−0.37172	−1.9694992
PG 36:3 PG 18:1–18:2	−0.37413	−1.98091823
PE 37:4 PE 17:0–20:4	−0.37732	−1.9960079
FA 18:4	−0.38291	−2.02250357
PE 37:4	−0.38539	−2.0342178
PG 36:4 PG 18:2–18:2	−0.39315	−2.07093777
PE 40:9	−0.40709	−2.13691562
PE 36:5 B	−0.46426	−2.40753065
PE 40:8 A	−0.53879	−2.76026439

### 2.10 Fold‐change analysis

All identified lipids were divided into 22 different classifications and analyzed by classification (sum of all lipids in the same classification) or by single lipids. Fold changes were calculated as mean condition B / mean condition A. The conditions are described in Table S1. Log_2_ of fold‐changes of the raw data were plotted vs. –Log_10_ FDR (*q* values) of the Johnson transformed data for each comparison as a heat map for lipid classifications (Figure 3) and volcano plot for single lipids (Figure 4). The reasoning behind using the *q*‐values of the Johnson transformed data is based on the non‐parametric nature of the data. This approach minimized the likelihood of spurious significance arising from distributional violations. For classification analysis, all fold‐changes and –Log_10_ FDR are plotted; however, changes with *q* > 0.1 (−Log_10_ FDR <1.0) are not considered significant. For single lipid analysis, values with a *q* > 0.1 (−Log_10_ FDR <1) were categorized as “no change” regardless of fold‐change value. The raw data for these plots, including specific names, can be found in the data repository.

Class lipid analysis for FAs, CE, DAGs, LPC, PC, and TAGs fold changes relative to LETO were calculated using the average of each class divided by the average for LETO CR Ctrl. The average, rather than the summation for lipid saturation, is due to the platform exhibiting higher sensitivity for detecting lipids with longer chain lengths. This approach normalization strategy mitigated the overrepresentation of highly detected species and allowed for a more balanced assessment across species. Thus, the fold‐change relative to LETO CR Ctrl was plotted for each group.

### 2.11 Correlations with Glucose & Insulin AUC, IRI, and changes in BM and WAT mass

Spearman's rank correlation coefficients (*ρ*) were calculated for parameters previously reported (Cornejo et al., 2020). Glucose and insulin AUCs, insulin resistance index (IRI), and changes in BM and white adipose tissue (WAT) mass were treated as independent variables, and lipid classifications were treated as dependent variables. IRI values were calculated using the glucose AUC divided by insulin AUC/100, after conducting an oral glucose tolerance test. Blood measurements were collected at 0, 15, 30, 60, and 120 min after a glucose bolus (2 g/kg) was administered to overnight‐fasted animals (14+). Commercially available blood glucose monitors and strips were used (CVS, Woonsocket, Rhode Island, 985464 and 391800). IRI was measured 7 days after ending CR, while terminal values (lipidomics, WAT mass, and BM) were measured 10 days after CR. This was done to avoid changes in terminal values due to 16 h fasting and glucose challenge. Correlations were considered strong at −0.8 < *ρ* > 0.8. Statistical analyses were performed in JMP pro (version 15, SAS Institute Inc., Cary, NC, USA).

## 3 RESULTS

### 3.1 PR reduced membrane FATP5 and CR reduced CD36 abundance in OLETF

**FIGURE 1:**
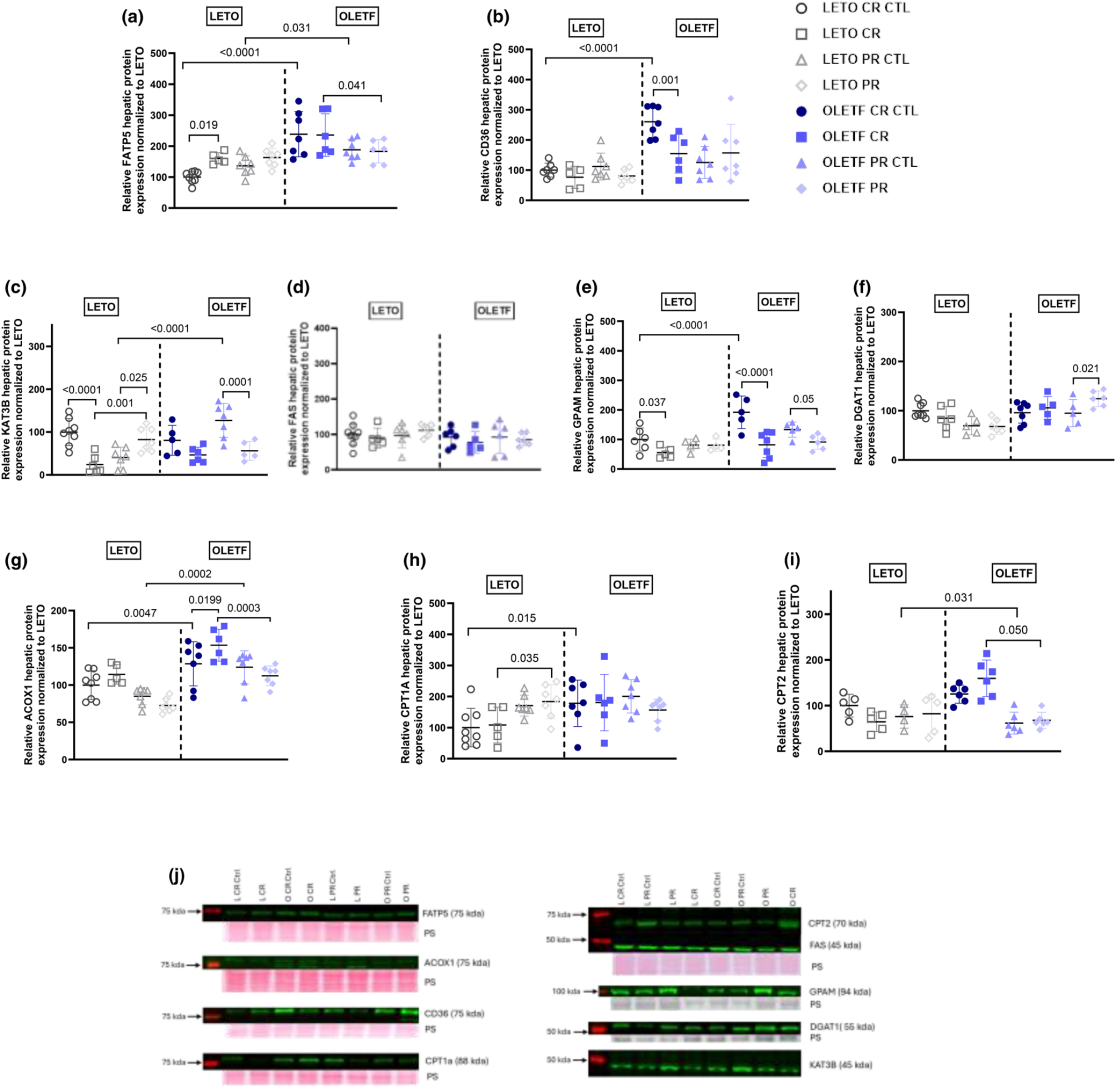
Partial body mass regain downregulated ACOX1 and CPT2 protein expression. Mean ± SE relative expressions of liver membrane (a) fatty acid transporter protein 5 (FATP5), (b) cluster differentiation 36 (CD36), (c) nuclear histone acetyltransferase p300 (KAT3B), and cytosolic (d) diacylglycerol O‐acyltransferase 1 (DGAT1), (e) glycerol‐3‐phosphate acyltransferase (GPAM), (f) fatty acid synthase (FAS), (g) acyl‐CoA oxidase 1 (Acox1), (h) carnitine palmitoyl transferase 1A (CPT1A), (i) carnitine palmitoyl transferase 2 (CPT2), and (j) ponceau stains.

At baseline, mean membrane FATP5 abundance in OLETF CR Ctrl was 138% greater than LETO CR Ctrl (*p* < 0.0001) and this continued as MetS progressed (*p* = 0.031). All OLETF groups, including CR, maintained elevated FATP5 expression levels. CR increased FATP5 abundance by 60% in LETO (*p* = 0.019). PR significantly reduced FATP5 expression by 22% (*p* = 0.041) compared to CR in OLETF, while maintaining levels similar to PR Ctrl (Figure 1a). Membrane CD36 was also greater in OLETF CR Ctrl compared to LETO CR Ctrl (160% higher; *p* < 0.0001); however, after CR, levels decreased 40% in OLETF (*p* = 0.001 vs. ad libitum Ctrl; Figure 1b).

### 3.2 PR maintained reduced KAT3B expression and increased DGAT1 in OLETF

Lysine acyltransferase (KAT3B) is a histone acetyltransferase enzyme that regulates lipogenic enzyme expression through the acetylation of transcription factors (Ponugoti et al., 2010). Thus, we sought to determine the potential transcriptional effect that PR has on lipogenesis. Nuclear KAT3B expression was not different between CR ad libitum LETO and OLETF groups. As MetS progressed, levels increased (222%) between OLETF PR Ctrl and LETO PR Ctrl groups (*p* < 0.0001) (Figure 1c). CR reduced (*p* < 0.0001) KAT3B expression by 76% compared to LETO CR Ctrl. Refeeding in the LETO PR induced a 241% increase (*p* = 0.001) in KAT3B compared to LETO CR, reversing the reduction induced by CR. Interestingly, this elevation in KAT3B was also significantly higher than LETO PR Ctrl (*p* = 0.025). In the OLETF strain, PR maintained lower KAT3B expression levels similar to the OLETF CR animals despite refeeding. Additionally, OLETF PR had lower (57%; *p* = 0.0001) KAT3B protein expression compared to OLETF PR Ctrl.

To assess the effects of PR on de novo lipogenesis, we measured crucial enzymes associated with the production of FAs and TAGs. Glycerol‐3‐phosphate acyltransferase 1, mitochondria (GPAM) is the rate‐limiting enzyme in TAG synthesis, and the last step of TAG production is catalyzed by diacylglycerol O‐acyltransferase (DGAT) (Yu et al., 2018). Cytosolic GPAM abundance was 92% greater in OLETF CR Ctrl than LETO CR Ctrl (*p* < 0.0001), demonstrating a strain effect. CR reduced GPAM hepatic expression in LETO (*p* = 0.037) and OLETF (*p* < 0.0001) when compared to its ad libitum Ctrl (Figure 1e). Both strains demonstrated that PR maintained this reduction in GPAM induced by CR; however, OLETF PR levels were significantly lower than OLETF PR Ctrl (57%; *p* = 0.05). Refeeding in the OLETF, PR demonstrated a significant increase (*p* = 0.021; 30%) in DGAT1 protein expression compared to its ad libitum Ctrl (Figure 1f). Aside from this, no other changes were seen in DGAT1 and FAS protein expression (Figure 1d,f).

### 3.3 PR decreased ACOX1 and CPT2 expression in OLETF

The expression of ACOX1 has been positively associated with the development of hepatic steatosis and its knockout has shown a reduction in hepatic fat accumulation, demonstrating its importance to hepatic fat availability (Yang et al., 2024). Basal cytosolic ACOX1 abundance demonstrated a significant increase (29%; *p* = 0.0047) in OLETF CR Ctrl compared to LETO CR Ctrl and continued as MetS progressed (*p* = 0.0002). CR induced a non‐significant increase in LETO. Conversely, in LETO, PR non‐significantly decreased ACOX1 by 27% relative to LETO CR (Figure 1g). In OLETF, CR significantly elevated ACOX1 expression (19%; *p* = 0.02) which was significantly reduced by PR (27%; *p* = 0.0003) similar to OLETF PR Ctrl levels. Carnitine palmitoyl transferase I (CPT1) protein expression also demonstrated a significant difference between the strains (*p* = 0.015) (Figure 1h). Refeeding in LETO significantly elevated CPT1A protein expression by 70% relative to CR (*p* = 0.035). Meanwhile, OLETF PR had 20% lower CPT1A expression than OLETF PR Ctrl, although not significant. CPT2 is the second rate‐limiting step of β‐oxidation and its expression is reduced during MASLD (Enooku et al., 2019). As MetS progressed, the hepatic protein expression of CPT2 was elevated in OLETF PR Ctrl relative to LETO PR Ctrl (*p* = 0.031). Although PR induced no change in the LETO animals, PR significantly reduced (44%; *p* = 0.050) CPT2 protein expression, similar to PR Ctrl compared to OLETF CR (Figure 1j). This reduction in CPT2 induced by PR in OLETF abolished the non‐significant increase seen in OLETF CR.

### 3.4 PR increased Total cholesterol and HDL in OLETF

**FIGURE 2:**
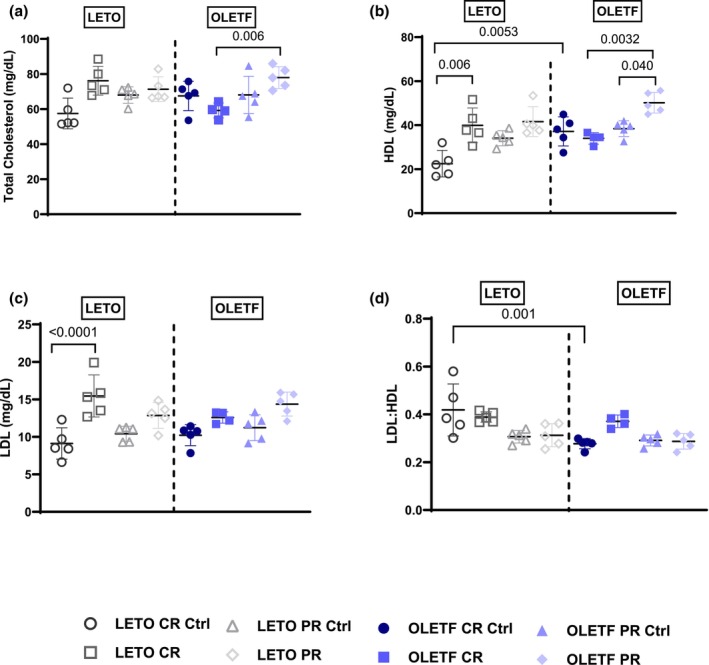
Partial body mass regain elevated total cholesterol and HDL. Mean ± SE for hepatic lipid content (a) total cholesterol, (b) HDL, (c) LDL, and (d) LDL:HDL.

To assess the effect of PR on plasma cholesterols, total cholesterol (TC), high‐density lipoprotein (HDL), and low‐density lipoprotein (LDL) levels were measured. CR non‐significantly increased TC levels by 32% in LETO but not OLETF. Conversely, levels increased by 64% (*p* = 0.006) in OLETF PR compared to OLETF CR, which was non‐significantly higher than baseline levels (Figure 2a). HDL levels were elevated in OLETF CR Ctrl compared to LETO CR Ctrl (*p* = 0.0053). In LETO, CR significantly increased HDL levels by 77% (*p* = 0.006) compared to LETO CR Ctrl, and this increase was maintained after PR. Interestingly, no significant difference was detected between LETO PR and LETO PR Ctrl. HDL levels increased 48% (*p* = 0.0032) in OLETF PR compared to OLETF CR and 31% (*p* = 0.04) compared to OLETF PR Ctrl (Figure 2b). CR increased LDL levels 70% (*p* < 0.0001) compared to its control (Figure 2c) in LETO and a non‐significant increase in OLETF. PR non‐significantly increased LDL levels compared to OLETF CR and OLETF PR Ctrl. The ratio between LDL and HDL can be used as an independent predictor of MASLD (Zou et al., 2021); thus, we calculated LDL:HDL for each group. LDL:HDL ratios were significantly reduced during MetS compared to LETO CR Ctrl (*p* = 0.001) (Figure 2d). In OLETF, CR non‐significantly increased the LDL:HDL ratio (34% increase) compared to OLETF CR Ctrl. Meanwhile, PR non‐significantly reduced the LDL:HDL ratio (22% decrease) relative to OLETF CR back to baseline.

### 3.5 Basal TAG, DAG, and PE increased in OLETF

**FIGURE 3:**
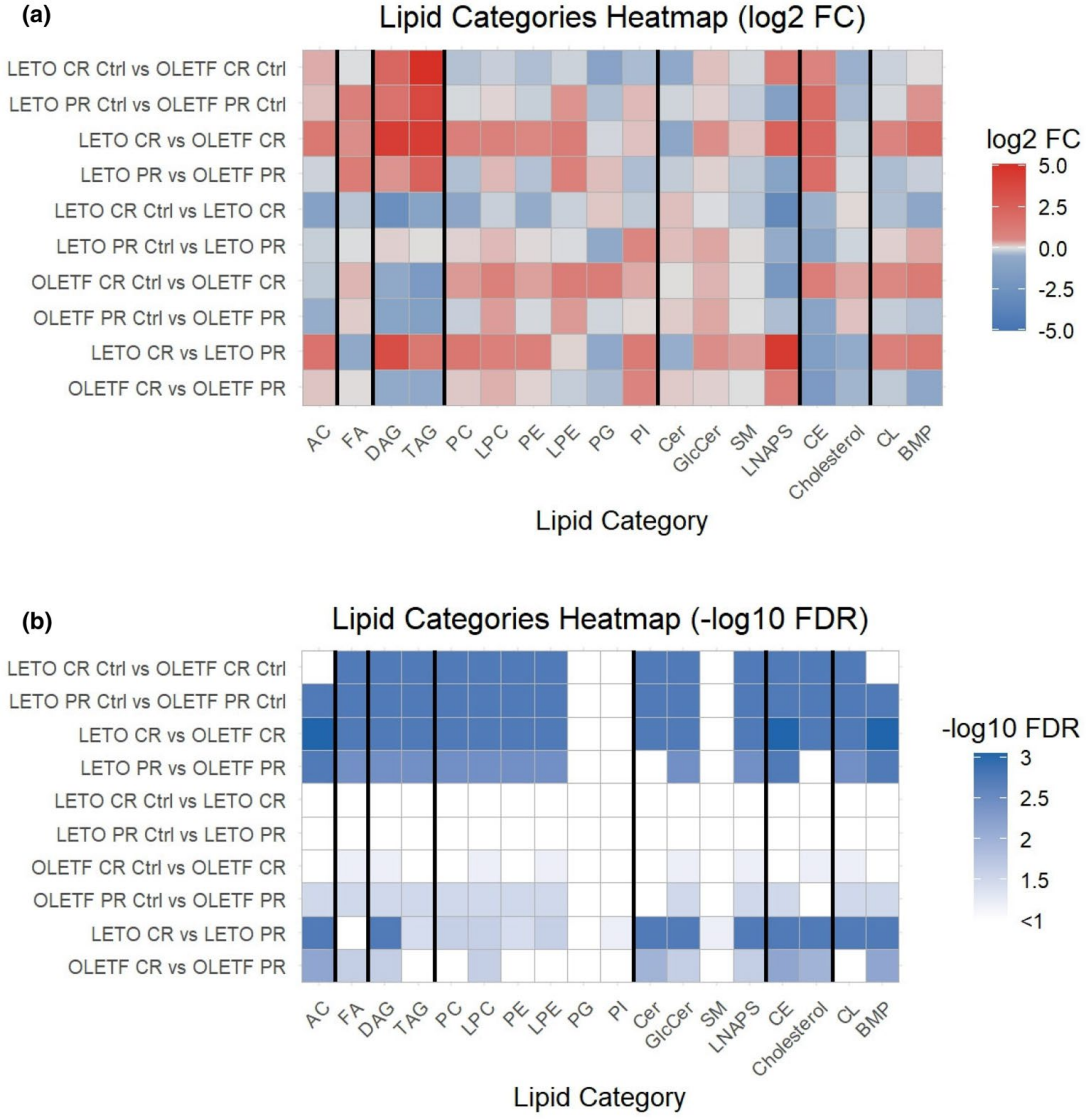
Partial body mass regain increased more lipid categories in LETO. Heat maps of (a) Log_2_ Fold‐change and (b) −Log_10_ FDR for lipids classification per comparison. The black lines separate distinct classes of lipids: (a) acylcarnitine: AC, (b) free fatty acids: FA, (c) glycerolipids: DAG and TAG, (d) glycerophospholipids: PC, LPC, PE, LPE, PG, and PI, (e) sphingolipids: Cer, GlcCer, SM, LNAPs, (f) sterol lipids: CE and cholesterol, and (g) organelle lipids: CL and BMP.

**FIGURE 4:**
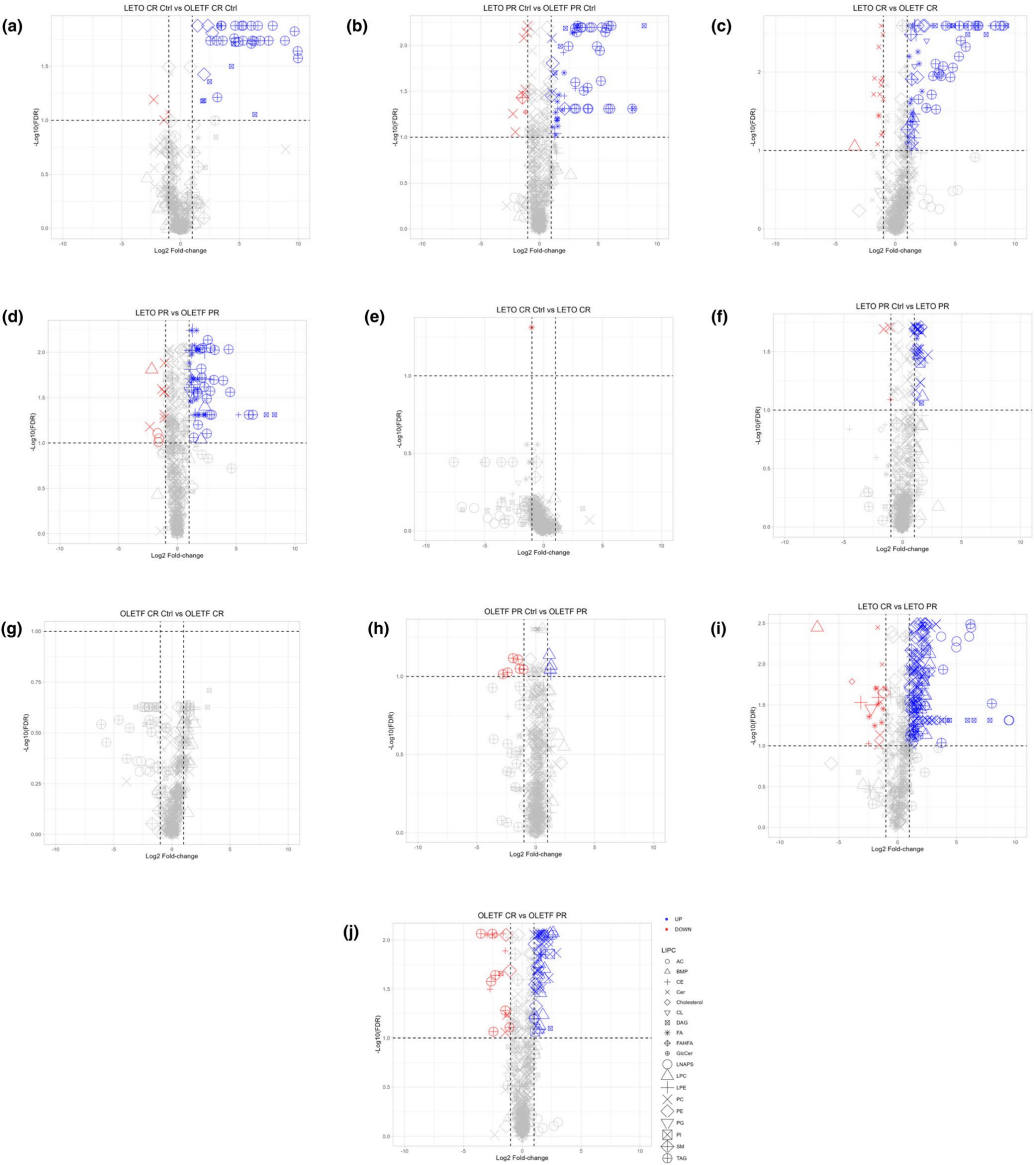
Partial body mass regain demonstrates more lipid fold‐changes in LETO. Volcano plots of Log_2_ fold changes vs. –Log_10_ FDR for single lipids by lipid classification for (a) LETO CR Ctrl vs. OLETF CR Ctrl, (b) LETO PR Ctrl vs. OLETF PR Ctrl, (c) LETO CR vs. OLETF CR, (d) LETO PR vs. OLETF PR, (e) LETO CR Ctrl vs. LETO CR, (f) LETO PR Ctrl vs. LETO PR, (g) OLETF CR Ctrl vs. OLETF CR, (h) OLETF PR Ctrl vs. OLETF PR, (i) LETO CR vs. LETO PR, and (j) OLETF CR vs. OLETF PR.

**FIGURE 5:**
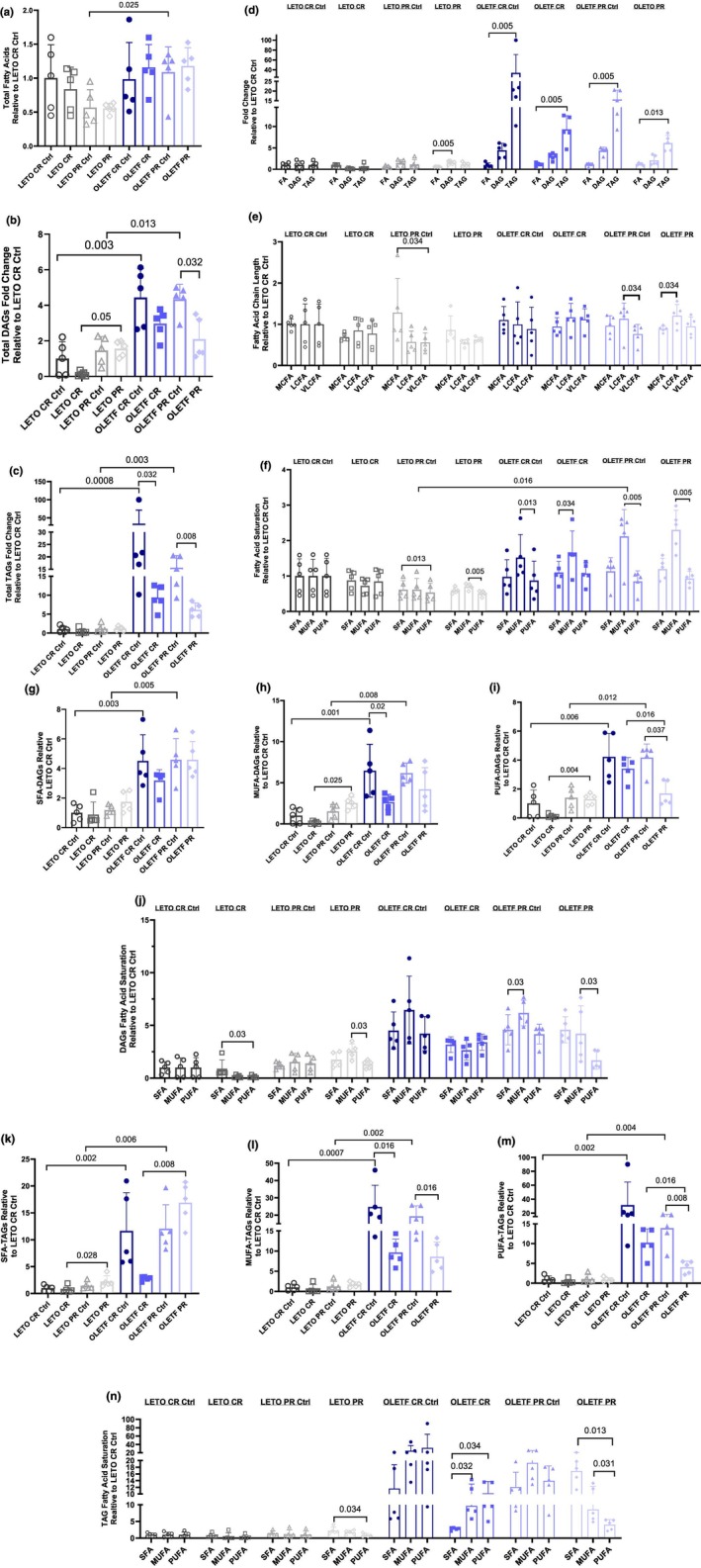
Partial body mass regain maintained reduced total DAGs and TAGs. (a) Total fatty acids, (b) total DAGs, (c) total TAGs, (d) total FAs, DAGs, and TAGs, (e) FAs chain length, (f) FAs saturation, (g) SFA‐DAGs, (h) MUFA‐DAGs, (i) PUFA‐DAGs, (j) DAG fatty acid saturation, (k) SFA‐TAGs, (l) MUFA‐TAGs, (m) PUFA‐TAGs, and (n) TAG fatty acid saturation relative to LETO CR Ctrl.

Total TAG and DAG were increased in OLETF CR Ctrl (33.3‐fold, *q* < 0.002 and 4.4‐fold, *q* < 0.002 respectively) compared to LETO CR Ctrl (Figure 3a,b). Moreover, several TAG and DAG species were increased as well (Figure 4a,b). When compared individually, several phosphatidylethanolamines (PE) were also increased in OLETF. Even more lipids were altered when comparing LETO PR Ctrl vs. OLETF PR Ctrl (Figure 4b), with increases in several fatty acids (FA) and total FAs (Figures 3a and 5a) in OLETF, which may suggest increased FA accumulation over the development of MetS in OLETF. All OLETFs demonstrated insignificantly lower total FAs (*p* < 0.01) than DAG; however, TAGs were significantly elevated relative to total FAs (*p* < 0.05) (Figure 5d).

### 3.6 PR maintained reduced Total DAGs and TAGs after CR compared to OLETF

PR in LETO significantly increased total DAGs by 990% (10.9 fold‐change; *p* = 0.05) compared to CR (Figures 3a and 5b). LETO CR demonstrated non‐significantly higher total FAs than DAGs, which was the opposite effect of PR where total DAGs were elevated compared to FAs (*p* = 0.005) (Figure 5d). In OLETFs, CR demonstrated a non‐significant decrease in total DAGs and significantly reduced total TAGs by 70% (*p* = 0.032) compared to OLETF CR Ctrl (Figure 5b,c). Interestingly, OLETF PR demonstrated similar total DAGs and TAGs compared to OLETF CR (Figure 5b,c).

### 3.7 PR increased SFA‐TAGs versus OLETF PR Ctrl

Differences in fatty acid saturation can be distinguished between the older OLETF PR Ctrl and LETO PR Ctrl. MUFAs were significantly elevated in OLETF PR Ctrl vs. LETO PR Ctrl (1.8‐fold‐change; *p* = 0.016) (Figure 5f). LETO PR demonstrated significantly elevated MUFAs relative to PUFAs (*p* = 0.013) (Figure 5f). Both OLETF PR (*p* = 0.005) and OLETF PR Ctrl (*p* = 0.005) demonstrated elevated MUFA levels relative to PUFA (Figure 5f).

Similarly, OLETF controls had elevated SFA‐DAGs (*p* < 0.01), MUFA‐DAGs (*p* < 0.01), and PUFA‐DAGs (*p* < 0.05) compared to their respective LETO control (Figure 5g–i). LETO CR demonstrated elevated SFA‐DAGs relative to PUFA‐DAGS (*p* = 0.03) (Figure 5j). LETO PR demonstrated elevated relative MUFA‐DAGs (16.5‐fold‐change; *p* = 0.03) and PUFA‐DAGs (9.5‐fold‐change; *p* = 0.004) compared to CR. In OLETF, CR significantly reduced MUFA‐DAGs (*p* = 0.02) by 30% while only exhibiting a non‐significant decrease for SFA‐DAGs and PUFA‐DAGs vs. OLETF CR Ctrl (Figure 5g–i). OLETF PR showed a non‐significant increase in SFA‐DAGs and MUFA‐DAGs relative to CR (Figure 5g,h). Unexpectedly OLETF PR significantly reduced PUFA‐DAGs compared to CR by 50% (*p* = 0.016) and PR Ctrl by 60% (*p* = 0.037) in OLETF (Figure 5i). Interestingly both LETO and OLETF PR groups demonstrated significantly lower PUFA‐DAGs relative to MUFA‐DAGs (*p* < 0.05) (Figure 5j).

Analogously to DAGs, TAGs showed SFA‐TAGs (*p* < 0.01), MUFA‐TAGs (*p* < 0.01), and PUFA‐DAGs (*p* < 0.01) in the OLETF controls versus LETO controls (Figure 5k–m). In the LETO group PR significantly increased SFA‐TAG by 2.3‐fold‐change (*p* = 0.028) compared to CR. Likewise, PR significantly elevated SFA‐TAG by 6.1‐fold‐change (*p* = 0.008) compared to the caloric restriction group in OLETF. OLETF PR also maintained the reduction in MUFA‐TAG (*p* = 0.016) induced by CR versus OLETF PR Ctrl (Figure 5l). Interestingly, PR further reduced PUFA‐TAGs compared to OLETF CR by 60% (*p* = 0.016) and OLETF PR Ctrl by 70% (*p* = 0.008) (Figure 5m).

### 3.8 PR reversed the TAG saturation profile observed in CR in OLETF

TAG fatty acid saturation profile changes were seen between CR and PR in OLETF (Figure 5n). OLETF CR demonstrated lower SFA levels than both MUFA and PUFA TAGS (*p* < 0.05). OLETF PR showed elevated SFA levels compared to PUFA (*p* < 0.05). Additionally, MUFA levels were also higher than PUFA levels (*p* < 0.05), but MUFA‐TAGs were non‐significantly lower than SFA in OLETF PR. PUFA had the lowest fold change compared to SFA and MUFA, which is the opposite of CR (Figure 5n). This change in saturation profile may be indicative of MASLD progression.

### 3.9 PR reduced the CE increase induced by CR in OLETF

**FIGURE 6:**
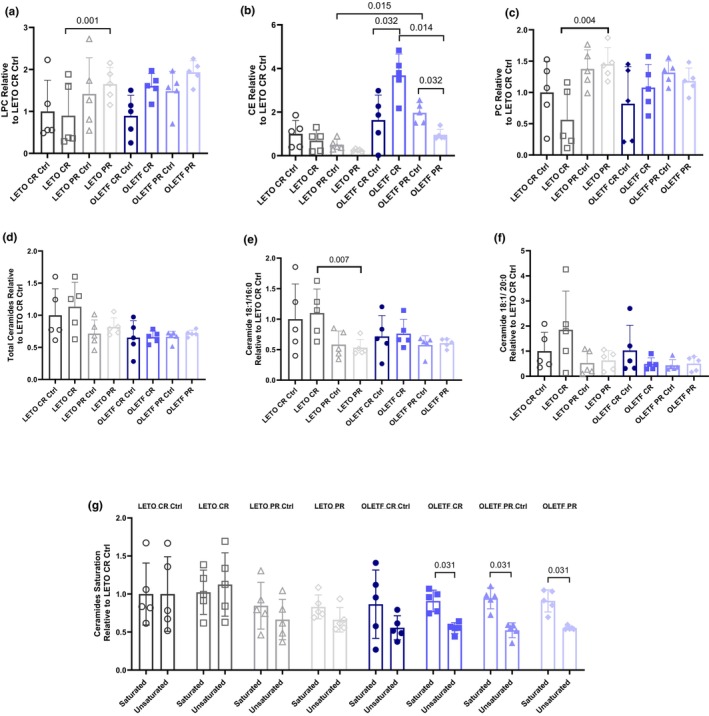
Partial body mass regain reduced hepatic CE levels. Total lipid analysis (a) LPC, (b) CE, (c) PC, and (d) ceramides relative to LETO CR Ctrl. Single lipid analysis for (e) ceramide 18:1/16:0 and (f) ceramide 18:1/20:0 relative to LETO CR Ctrl. (g) Ceramide saturation relative to LETO CR Ctrl.

Caloric restriction significantly increased CE by 2.1‐fold‐change (*p* = 0.034) in OLETF vs. ad libitum Ctrl (Figure 6b). Conversely, OLETF PR reduced CE levels achieved by OLETF CR (0.3‐fold‐change; 70%; *p* = 0.014) and OLETF PR Ctrl (50%; *p* = 0.032). In LETO, LPC levels were also elevated by PR (*p* < 0.05) relative to CR (Figure 6a). Changes in PC were only detected in the LETO animals where PR (2.6‐fold‐change; *p* < 0.05) abolished CR's non‐significant reduction and elevated levels back to its ad lib Ctrl (Figure 6c). In LETO, PR lowered ceramide NS 18:1/16:0 by 50% (*p* < 0.01) from the CR state, returning to baseline levels (Figure 6e). No statistically significant changes were noticed for total ceramides and ceramide NS 18:1/20:0 (Figure 6d,f). However, regarding ceramide saturation, all OLETF groups except OLETF CR Ctrl demonstrated higher saturated ceramides compared to unsaturated ceramides (Figure 6g).

### 3.10 PR increased more lipid categories in LETO compared to OLETF

In LETO, PR increased total bis(monoacylglycero)phosphate (BMP) (2.5‐fold‐change; *q* = 0.002), cardiolipin (CL; 1.8‐fold‐change; *q* = 0.002), DAG (10.9‐fold‐change; *q* = 0.002), lysophosphatidylethanolamine (LPE; 1.1‐fold‐change; *q* = 0.025), PC (2.6‐fold‐change; *q* = 0.025), PE (1.8‐fold‐change; *q* = 0.04), phosphatidylinositol (PI; 2.2‐fold‐change; *q* = 0.06), sphingomyelin (SM; 1.3‐fold‐change; *q* = 0.06), and TAG (2.5‐fold‐change; *q* = 0.04) compared to LETO CR group (Figure 3a,b). Total acylcarnitine (AC), glucosylceramide (GlcCer), N‐acyl‐lysophosphatidylserine (LNAPS), and LPC species were increased in both strains after PR. Conversely, PR increased total ceramide (Cer; 1.1‐fold‐change; *q* = 0.01) while decreasing total BMP (0.6‐fold‐change; *q* = 0.007) and DAG (0.7‐fold‐change; *q* = 0.025) in OLETF PR vs. OLETF CR (Figure 3a,b). PR reduced total cholesterol in both strains after caloric restriction. Moreover, more single lipids were increased in LETO compared to OLETF after PR. Additionally, LETO PR also increased several LPC, PC, and PE, which were also seen in OLETF PR, to a lesser degree. PR showed an increase in hepatic TAG species after CR in LETO, but a non‐significant decrease in the OLETF (Figure 3a,b).

### 3.11 IRI had the Most direct correlations in LETO

IRI levels were previously published (Cornejo et al., 2020). IRI levels for LETO were between 5 and 8 and for OLETF 15–20. Levels of cardiolipin (CL) (*ρ* = 0.9), DAG (*ρ* = 0.9), LNAPS (*ρ* = 0.9), PC (*ρ* = 0.9), PE (*ρ* = 0.9), PI (*ρ* = 0.9) were strongly and directly correlated with IRI in LETO CR Ctrl. In the OLETF CR Ctrl group, only the levels of LNAPS and WAT mass were strongly correlated (*ρ* = −0.9; Figure S3A,B). However, as MetS progressed, the OLETF PR Ctrl group demonstrated that more correlations were detected, including DAG with glucose AUC and IRI, AC and ceramides with insulin AUC and body mass (BM), and cholesterol with white adipose tissue (WAT), all with *q* = 0.9 and *p* = 0.05.

### 3.12 Insulin AUC and WAT mass were more directly correlated with lipid categories than BM changes

Hepatic total cholesterol (*ρ* = 0.9), DAG (*ρ* = 1), LNAPS (ρ =0.9), PC (*ρ* = 0.9), PI (*ρ* = 0.9), PS (*ρ* = 0.9), SM (ρ =1), and TAG (*ρ* = 0.9) were directly and positively correlated with WAT mass in OLETF after PR (Figure S3A,B). FA (*ρ* = −1) and PG (*ρ* = −1) were negatively correlated with WAT mass during PR in OLETF. Insulin AUC was also directly correlated with the same lipids as WAT mass, including Cer (*ρ* = 0.9) and CL (*ρ* = 0.9). In LETO, GlcCer (*ρ* = −0.9) and LNAPS (*ρ* = −0.9) were strongly and inversely correlated with glucose AUC after CR (Figure S3A,B). Conversely, in OLETF, CR did not induce any positive or negative correlations (0.8 > ρ <0.8).

### 3.13 PCA successfully separated each pair of groups

Each pairwise comparison had significantly different (*p* < 0.05) principal component (PC) score means, except for LETO CR vs. LETO CR Ctrl (Comparison E; *p* = 0.134) and OLETF PR vs. OLETF PR Ctrl (Comparison H; *p* = 1) (Table S2). All groups were compared in the same PC plot; however, different PCs were used to differentiate the comparisons (Figure S2).

### 3.14 TAG and DAG contributed the Most to the distinction between LETO and OLETF ad libitum controls, but not after CR or PR

For both strains, comparisons A (CR Ctrl) and B (PR Ctrl) were separated by different principal components, hence different positive and negative loadings (Table 2, Comparisons A, B). Comparison A was differentiated by principal component 2, reflecting TAG species enrichment for OLETF CR Ctrl and Cer species enrichment for LETO CR Ctrl (Table 2, PC2). The progression of MetS induced a change in the lipidomic profile, where PC3 showed that FAs characterized OLETF PR Ctrl, and both PC and LNAPS characterized LETO PR Ctrl (Table 2, PC3). Nonetheless, when comparing both strains after CR, TAG species contributed to LETO and OLETF the most, while ceramides further characterized LETO (Table 2, PC2). Following PR, PC and LNAPS species had the greatest weight in differentiating LETO, while LPE and FA differentiated OLETF the most (Table 2, PC4).

### 3.15 Lipid classifications that distinguish CR from control in LETO are different than in OLETF

In LETO, most of the top 20 lipids that distinguished CR were Cer and SM, while TAGs differentiated CR control (Table 2, PC2). In OLETF, all top 10 lipids that differentiated CR Ctrl were Cer, PE, SM, and DAG, while CR was characterized by PE, PC, PG, and FA (Table 2, PC5). Both LETO PR Ctrl and PR were differentiated by different PC and PE lipids. The chain length that influenced the separation of PR in LETO was shorter than that of its PR control (Table 2, PC4). The top 20 lipids that separated OLETF PR vs. OLETF PR Ctrl were the same as CR (Table 2, PC5). The top 20 lipids that characterized CR in LETO were SM, PE, LPE, and Cer, meanwhile, for PR it was only PC (Table 2, PC1). For OLETF, PR vs. PR Ctrl, the top 20 lipids that segregated the groups were PCs and PEs; however, PR demonstrated short chain lengths of PC and PEs compared to PR Ctrl (Table 2, PC4).

## 4 DISCUSSION

**FIGURE 7:**
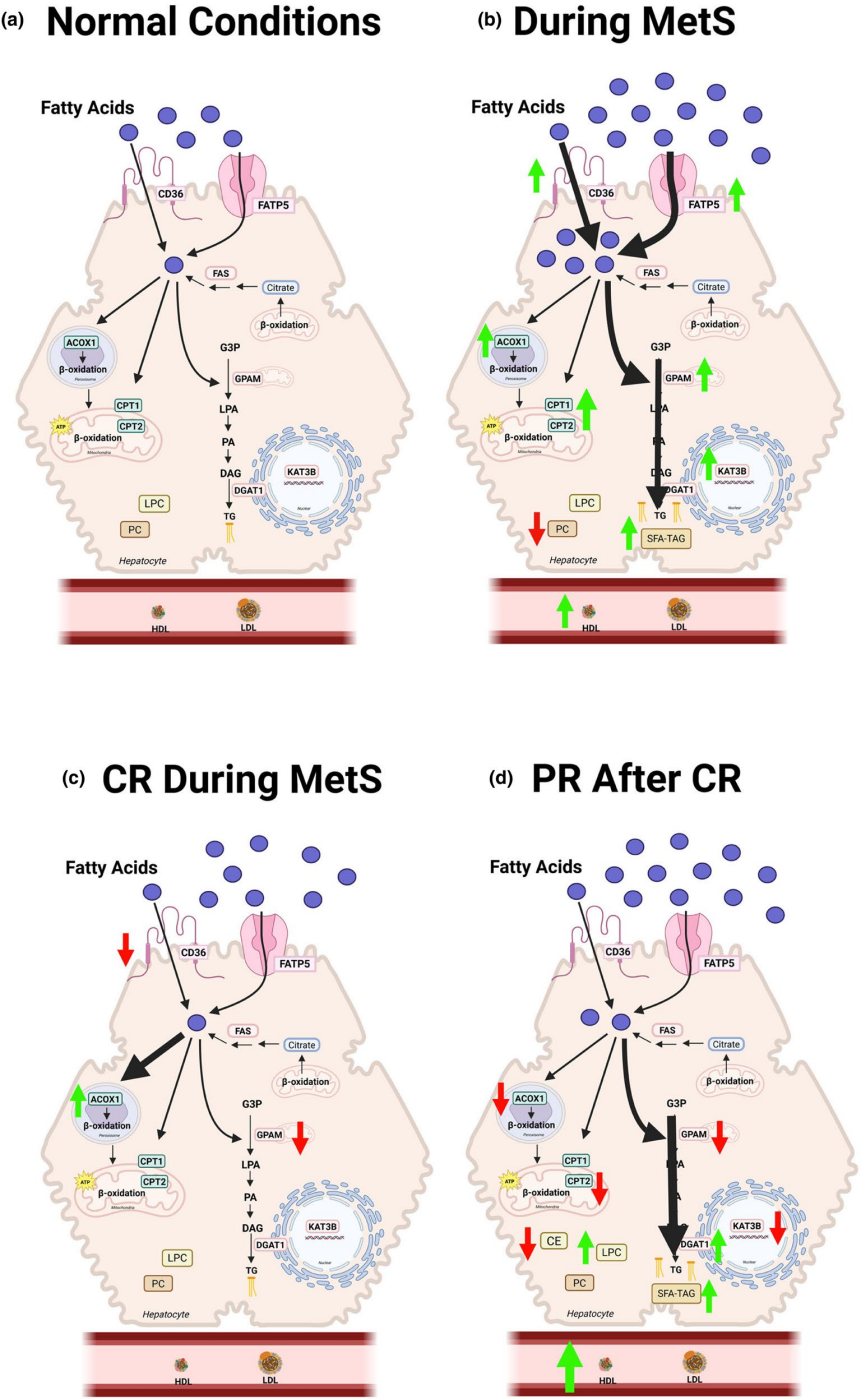
Schematic demonstrating the changes in hepatocytes seen during MetS, CR, and PR in this study.

Caloric restriction is a common behavioral intervention to ameliorate the detriments of metabolic defects associated with MetS. Previous studies have shown that CR ameliorates MetS and improves insulin resistance status (assessed by IRI) (Cornejo et al., 2020). However, recent literature focuses primarily on CR while neglecting the effects of non‐compliance, the subsequent body mass regain and their effects on the hepatic lipidome. For example, previous research has shown that contestants in a rapid weight loss contest regained a substantial amount of BM and metabolic impairment, demonstrating the detriments of BM regain without defined mechanisms (Fothergill et al., 2016). Thus, this study aimed to better understand the potential detriments of BM regain following CR on liver health and the hepatic lipidome during MetS, and insights into some of the mechanisms regulating lipid metabolism in this setting (Figure 7).

### 4.1 Partial BM regain elevated SFA‐TAGs correlated with MASLD

The results demonstrated that total DAG and TAG were significantly reduced after PR similar to CR in OLETF, which is typically associated with MASLD prevention. However, these reductions are primarily associated with MUFA and PUFA DAGs and TAGS but not SFAs. The data demonstrated an non‐significant increase in SFA‐DAGS with an elevation in SFA‐TAGs after PR. Saturated fatty acid enriched TAGS have been associated with both MASH and MASLD patient livers, demonstrating lipid remodeling and change in de novo lipogenesis (Chiappini et al., 2017; Puri et al., 2007; Walle et al., 2016). Collectively, these data demonstrate that PR may contribute to promoting MASLD, and if left to progress, may lead to MASH via upregulation of SFA‐TAGs.

### 4.2 Partial BM regain promoted hepatic fat accumulation by downregulating fatty acid utilization

During fasting, plasma FAs are the primary substrate for hepatic triglyceride production (Donnelly et al., 2005). In the liver, FATP5 is a predominant FATP isoform. Hepatic FATP5 expression is elevated during the initial stages of MASLD and reduces as the disease progresses (Enooku et al., 2020). Additionally, silencing FATP5 in mice in diet‐induced MASLD reduced hepatic fatty acid uptake (Doege et al., 2008), demonstrating that FATP5 is a primary hepatic FA transporter. In this study, PR maintained elevated FATP5 protein expression similar to its MetS‐induced MASLD control. This suggests that PR may not be attenuating enhanced FA mobility into the liver, which can lead to MASLD. To further examine the effects of PR on hepatic fat accumulation, markers of fatty acid oxidation were measured. ACOX1 is the rate‐limiting enzyme in the peroxisomal β‐oxidation pathway known for the catabolism of very long‐chain fatty acids (VLCFAs). Excess hepatic fat accumulation has been detected in mice with ACOX1 mutations, indicating the importance of ACOX1's presence for lipid utilization (Sheridan et al., 2011). Once VLCFAs are broken down into 8–12 carbon chains, they are transported to the mitochondria to be further catabolized by β‐oxidation. CPT2, the second rate‐limiting enzyme of mitochondrial β‐oxidation, is downregulated during MASLD (Enooku et al., 2019). The data demonstrated that the reduction induced by PR in OLETF is similar to that of its control for ACOX1 and CPT2 protein expression, suggesting that regaining BM had deleterious effects on fatty oxidation enzymes, potentially impairing efficient FA utilization and promoting fat accumulation.

### 4.3 Partial BM regain upregulated LPC: Marker of hepatic damage

Lysophosphatidylcholine (LPC) is a lipid produced when PC is cleaved by phospholipase A_2_. Elevated levels of LPC are typically correlated with cardiovascular disease, diabetes, renal failure, and other detriments (Rabini et al., 1994). In endothelial cells, LPC increases reactive nitrogenous species, demonstrating its potential to promote nitrosative damage (Kim et al., 2009) and (Sasagawa et al., 1998). Chronic elevations in LPC have also been shown to reduce mitochondrial β‐oxidation in hepatocytes and alter mitochondrial integrity by increasing mitochondrial permeability, demonstrating its deleterious effects on cell viability and hepatic health (Hollie et al., 2014). The data demonstrated in both volcano plots and heatmap comparisons between OLETF PR vs. OLETF OLETF PR Ctrl and OLETF PR vs. OLETF CR an increase in total and various species of LPCs. The results here suggest that LPC may be one of the primary contributors to the reduction in CPT2 expression. Furthermore, the current findings suggest that prolonged PR may induce determinants to hepatic health and function via LPC‐mediated impairments on mitochondrial function, but more research is required.

### 4.4 Partial BM regain increased HDL and changes in hepatic lipidome: HDL Dysfunction?

For years an association between HDL levels and its protective effects against CVD has been established (Trieb et al., 2020). Similarly, several studies have demonstrated a reduction in HDL availability during MASLD emphasizing the importance of proper HDL levels (Bril et al., 2016; DeFilippis et al., 2013; Ren et al., 2019). Conversely, current data have demonstrated that inappropriately elevated HDL levels may be indicative of dysfunctional HDL metabolism, which promotes inflammation and cardiac damage (Smith, 2010). Additionally, T2DM has been associated with impairing HDL's anti‐inflammatory and antioxidant properties (Morgantini et al., 2011). There are key changes in the lipidome including an increase in PC, LPC, free cholesterol, and the reduction of CE and membrane phospholipids during HDL dysfunction (Pruzanski et al., 2000; Wiesner et al., 2009). This study demonstrated similar changes in hepatic lipidome following PR, which were positively correlated with LPC and negatively correlated with CE and elevated HDL levels in OLETF. These data suggest that the elevated HDL levels and specific signatures in the hepatic lipidome are potentially indicative of HDL dysfunction, which may potentially contribute to the manifestation of MASLD during MetS.

### 4.5 Partial body mass regain abolishes caloric Restriction's benefits

MAFLD is associated with an increase in TAG, which may be attenuated during CR, indicating that severe CR is characterized by a shift in lipid metabolism due to a reduced TAG availability (Steinhauser et al., 2018). In OLETF, reduced GPAM expression and TAG levels during CR suggest suppressed TAG synthesis, while increased CE levels indicate a shift from TAGs to CEs, which is associated with decreased risk of diabetes (Rhee et al., 2011). FA utilization is crucial during periods of low‐energy availability, such as fasting (Donnelly et al., 2005). Previously, we showed an increasing trend in hepatic NEFA levels after CR in OLETF (Cornejo et al., 2020) and in total FAs (Figure 5a). Consistent with this, the upregulation in ACOX1 and maintained FATP5 levels suggest that the sequestered FAs are for mitochondrial energy production rather than TAG production. Finally, reduced PCs may serve as a biomarker of hepatic steatosis because their reduction is associated with liver diseases (Chen et al., 2019). PCs were among the top lipids associated with CR, demonstrating reciprocal changes in PCs and TAGs in OLETF. This finding is congruent with others regarding increases in PC correlating with healthier individuals that have reduced TAG synthesis during CR. Collectively, the data demonstrate that CR shifted lipid metabolism during MetS in a multitude of ways to potentially improve hepatic health. Despite these benefits, PR downregulated lipid utilization, increased SFA‐TAGs, was negatively correlated with CEs, and non‐significantly upregulated PCs, which are all associated with MASLD. These findings further emphasize the detriments of PR by nullifying the benefits of CR and reverting the liver back to a compromised condition.

### 4.6 MetS promoted hepatic steatosis by overexpressing the first step of TAG synthesis

In the present study, hepatic CD36 and FATP5 protein expressions were elevated in the hyperinsulinemic OLETF rats compared to LETO, suggesting that the chronic and statistically elevated insulin levels in OLETF may contribute to hepatic TAG accumulation by increasing insulin‐mediated TAG synthesis maintained by sustained FFA sequestration. These FFAs can then be used in the initial step in TAG synthesis that is mediated by GPAM, which can be stimulated by elevated insulin (Yu et al., 2018). Our findings also showed that GPAM expression was elevated in OLETF rats associated with greater hepatic TAG and DAG levels compared to LETO rats, suggesting that the hyperinsulinemia characterized by MetS may promote TAG accumulation via the increased expression of GPAM. Additionally, ACOX1 was greater in OLETF compared to LETO rats. This apparent paradox (increased TAG synthesis in the presence of increased ACOX1, a β‐oxidation enzyme) may represent an adaptive, compensatory response to help ameliorate the increase in TAG. Regardless, the accumulation of hepatic TAG during MetS conditions suggests that synthesis is greater than utilization and is indicative of impaired lipid metabolism characteristic of MetS‐associated MASLD.

## 5 LIMITATIONS

A limitation of this study is not measuring PC and PE levels individually. Current literature has suggested that a PC:PE ratio is indicative of hepatic steatosis (Arendt, 2013). Furthermore, CE concentrations would give further insight into the effects of insulin resistance on HDL dysfunction. Future investigations should explore the detriments of complete body mass regain and the potential of body mass cycling on MASLD.

## 6 CONCLUSIONS

In conclusion, lipogenesis does not appear to contribute significantly to the partial body mass regain and hepatic lipid accumulation following CR, but instead hepatic accumulation is most likely due to downregulated utilization, which can promote further hepatic injury during MetS (Figure 7). This partial regain in BM abolished the CR benefits that included upregulating lipid utilization. Additionally, the increases in HDL, LPC, and reduction in CE in the OLETF PR animals are characteristic of HDL dysfunction, indicating a potential connection between HDL dysfunction and MASLD development. Furthermore, the increase in SFA‐TAGs, which are elevated during MASLD, demonstrates the detriments of partial body mass regain. Overall, these findings demonstrate that 1 week of refeeding is sufficient to abolish the benefits of CR and induce changes in the hepatic lipidome that can promote MASLD and its associated injury during the regain phase. This is critical because it emphasizes the importance of maintaining the CR‐reduction in body mass during MetS.

## AUTHOR CONTRIBUTIONS

**Eira E. Jardines:** Conceptualization; data curation; formal analysis; investigation; methodology; validation; visualization. **Manuel A. Cornejo:** Conceptualization; data curation; formal analysis; methodology; validation; visualization. **John W. Newman:** Formal analysis; methodology; visualization. **Oliver Fiehn:** Methodology. **Akira Nishiyama:** Investigation; resources. **Rudy M. Ortiz:** Funding acquisition; resources; supervision.

## FUNDING INFORMATION

This work was supported by GRISE Bio‐I‐STeP (5T32GM141862‐02); American Heart Association (23DIVSUP1066372); UC MEXUS‐CONACY (T440553); and the National Institute on Minority Health and Health Disparities grant (9T37‐MD001480).

## CONFLICT OF INTEREST STATEMENT

The authors declare no conflicts of interest.

## ETHICS STATEMENT

All experimental procedures conducted were reviewed and approved by the institutional animal care and use committee of Kagawa Medical University (Kagawa, Japan).

## DECLARATION OF GENERATIVE AI AND AI‐ASSISTED TECHNOLOGIES IN THE WRITING PROCESS

During the preparation of this work, the author(s) used chatgpt to concise and improve the readability of the article. After using this tool/service, the author(s) reviewed and edited the content as needed and take full responsibility for the content of the published article.

## MATERIALS AVAILABILITY

This study did not generate new unique reagents.

## Supporting information


Data S1.

**Table S1.** Fold changes were calculated as mean condition B/mean condition A, after mTIC normalization.
**Table S2.** Mean PC score ± SD for PC1 and PC2 for each group comparison and *t*‐test *p* value for each comparison.
**Table S3.** PCA1.
**Table S4.** PCA 2.
**Table S5.** PC3.
**Table S6.** PC4.
**Table S7.** PC5.
**Figure S1.** Study design.
**Figure S2.** PCA for each comparison.
**Figure S3.** Insulin AUC and WAT mass had the most lipid category correlation during PR.

## Data Availability

The data for the hepatic lipidome analysis have been uploaded to figshare: https://doi.org/10.6084/m9.figshare.28765712.v1 with free accessibility. Any additional information needed to reanalyze the data reported in this paper is available from the lead contact. This paper does not report original code.
