# Supplementary material for: Partial body mass regain attenuates lipid utilization and alters the hepatic lipidome linked to HDL dysfunction during metabolic syndrome in male rats
Source: Physiol Rep. 2026 Jun 16;14(12):e70865. doi: 10.14814/phy2.70865 (PMC13273030; doi:10.14814/phy2.70865)
Supplement: Supplementary file 2 — Table S1. Fold changes were calculated as mean condition B/mean condition A, after mTIC normalization. Table S2. Mean PC score ± SD for PC1 and PC2 for each group comparison and t‐test p value for each comparison. Table S3. PCA1. Table S4. PCA 2. Table S5. PC3. Table S6. PC4. Table S7. PC5. Figure S1. Study design. Figure S2. PCA for each comparison. Figure S3. Insulin AUC and WAT mass had the most lipid category correlation during PR. [file PHY2-14-e70865-s002.docx]

**Table S1.** Fold changes were calculated as mean condition B / mean condition A, after mTIC normalization.

| Condition A | | |  | Condition B | |  |  | Comparison |
| --- | --- | --- | --- | --- | --- | --- | --- | --- |
| Strain | | **Treatment** |  | **Strain** | **Treatment** |  |  |  |
|  | *Obesity Effects* | | | | | | | |
| LETO | | CR Ctrl |  | OLETF | CR Ctrl |  |  | A |
| LETO | | PR Ctrl |  | OLETF | PR Ctrl |  |  | B |
| LETO | | CR |  | OLETF | CR |  |  | C |
| LETO | | PR |  | OLETF | PR |  |  | D |
|  | *Treatment Effects* | | | | | | | |
| LETO | | CR Ctrl |  | LETO | CR |  |  | E |
| LETO | | PR Ctrl |  | LETO | PR |  |  | F |
| OLETF | | CR Ctrl |  | OLETF | CR |  |  | G |
| OLETF | | PR Ctrl |  | OLETF | PR |  |  | H |
|  | *Partial Regain Effects* | | | | | | | |
| LETO | | CR |  | LETO | PR |  |  | I |
| OLETF | | CR |  | OLETF | PR |  |  | J |

CR: Caloric Restriction; LETO: Long-Evans Tokushima Otsuka; OLETF: Otsuka Long-Evans Tokushima Fatty; PR: Partial Regain

**Table S2**. Mean PC score ± SD for PC1 and PC2 for each group comparison and T-test P value for each comparison.

| Comparison | Group | PC | Mean PC | *P* value |
| --- | --- | --- | --- | --- |
| A | LETO CR Ctrl | 2 | 7.1 ± 7.9 | 0.0122 |
|  | OLETF CR Ctrl | 2 | -11.1 ± 6.1 |  |
| B | LETO PR Ctrl | 3 | -8.6 ± 9.8 | 0.037 |
|  | OLETF PR Ctrl | 3 | 4.3 ± 5.7 |  |
| C | LETO CR | 2 | 10.6 ± 3.1 | 0.0122 |
|  | OLETF CR | 2 | -4.8 ± 3.9 |  |
| D | LETO PR | 3 | -5.3 ± 3.6 | 0.0122 |
|  | OLETF PR | 3 | 7.7 ± 2.0 |  |
| E | LETO CR Ctrl | 2 | 7.1 ± 7.9 | 0.83 |
|  | LETO CR | 2 | 10.6 ± 3.1 |  |
| F | LETO PR Ctrl | 4 | 4.8 ± 3.8 | 0.0122 |
|  | LETO PR | 4 | -6.3 ± 2.1 |  |
| G | OLETF CR Ctrl | 5 | 5.9 ± 4.9 | 0.0122 |
|  | OLETF CR | 5 | -3.2 ± 3.0 |  |
| H | OLETF PR Ctrl | 3 | 4.3 ± 7.6 | 1 |
|  | OLETF PR | 3 | 7.8 ± 2.0 |  |
| I | LETO CR | 1 | -14.1 ± 16.6 | 0.0122 |
|  | LETO PR | 1 | 14.2 ± 5.5 |  |
| J | OLETF CR | 4 | -8.4 ± 6.7 | 0.0122 |
|  | OLETF PR | 4 | -2.9 ± 2.2 |  |

CR: Caloric Restriction; LETO: Long-Evans Tokushima Otsuka; OLETF: Otsuka Long-Evans Tokushima Fatty; PC: Principal Component; PR: Partial Recovery

**Table S3**. PCA1

| **Lipids** | **PCA 1 Loadings** | **PC1_Zscore** |
| --- | --- | --- |
| PC 38:5 A (2) | 0.945 | 1.234 |
| PC 40:6 A (2) | 0.940 | 1.224 |
| PC 38:3 A | 0.936 | 1.215 |
| PC 38:5 B (2) | 0.926 | 1.193 |
| PC 36:5 C | 0.921 | 1.183 |
| PC 35:3 | 0.920 | 1.181 |
| PC 37:5 | 0.912 | 1.164 |
| PC 36:5 D | 0.911 | 1.163 |
| PC 35:2 B | 0.909 | 1.157 |
| PC 34:3 B | 0.908 | 1.156 |
| BMP 44:12 BMP 22:6-22:6 | 0.907 | 1.153 |
| PC 37:2 (2) | 0.906 | 1.152 |
| PC 40:5 A (2) | 0.906 | 1.152 |
| LPE 22:5 | 0.903 | 1.146 |
| PC 32:2 (2) | 0.903 | 1.146 |
| PC 36:2 (2) | 0.900 | 1.139 |
| PE 38:5 PE 18:1-20:4 | 0.899 | 1.136 |
| PE 36:3 A | 0.898 | 1.134 |
| PC 35:1 (2) | 0.897 | 1.131 |
| PC 36:3 A (2) | 0.896 | 1.129 |
| PC 32:2 | 0.894 | 1.126 |
| PC 34:3 A | 0.893 | 1.123 |
| PE 38:3 PE 18:0-20:3 | 0.892 | 1.121 |
| PC 34:2 (2) | 0.891 | 1.119 |
| PC 36:4 A (2) | 0.890 | 1.118 |
| PC 38:2 | 0.890 | 1.118 |
| PE 36:3 B | 0.890 | 1.118 |
| PE 38:5 A | 0.889 | 1.115 |
| PC 36:5 B | 0.888 | 1.112 |
| PC 40:5 A | 0.886 | 1.109 |
| PC 39:6 | 0.885 | 1.107 |
| PC 33:2 (2) | 0.885 | 1.107 |
| PE 38:4 PE 18:1-20:3 | 0.884 | 1.105 |
| PC 37:4 (2) | 0.884 | 1.105 |
| PE 34:3 PE 16:1-18:2 | 0.883 | 1.102 |
| PE 34:2 (2) | 0.883 | 1.102 |
| PC 38:2 (2) | 0.881 | 1.099 |
| PC p-36:5/PC o-36:6 | 0.880 | 1.097 |
| CL 70:7 CL 34:3-36:4 | 0.880 | 1.095 |
| PC 40:4 (2) | 0.879 | 1.093 |
| PC 36:3 A | 0.878 | 1.091 |
| LPE 18:1 | 0.877 | 1.091 |
| PI 36:3 PI 16:0-20:3 | 0.876 | 1.088 |
| PC 35:2 | 0.876 | 1.088 |
| LPC 22:6 2 | 0.876 | 1.088 |
| PC 38:5 B | 0.875 | 1.085 |
| PC 40:6 B (2) | 0.874 | 1.084 |
| PC 38:6 A | 0.874 | 1.084 |
| LPC 22:6 | 0.873 | 1.082 |
| PC 36:2 | 0.873 | 1.082 |
| PC 42:6 | 0.872 | 1.078 |
| PC 37:2 | 0.871 | 1.078 |
| PI 38:5 | 0.870 | 1.075 |
| PE 34:3 PE 16:0-18:3 | 0.870 | 1.075 |
| PE 34:1 | 0.869 | 1.073 |
| PC 33:1 (2) | 0.868 | 1.071 |
| PC 38:5 A | 0.868 | 1.071 |
| PC 35:2 A | 0.867 | 1.069 |
| PE 36:5 PE 16:1-20:4 | 0.866 | 1.067 |
| PC 34:3 | 0.866 | 1.066 |
| PC 36:3 B (2) | 0.865 | 1.065 |
| LPC 14:0 | 0.865 | 1.065 |
| PC 38:6 B | 0.861 | 1.056 |
| SM d41:2B 2 | 0.860 | 1.053 |
| PE 36:3 | 0.859 | 1.050 |
| PI 36:4 | 0.858 | 1.050 |
| PE 36:5 A | 0.857 | 1.047 |
| PC 40:7 B | 0.857 | 1.047 |
| LPC 22:5 | 0.857 | 1.047 |
| LPC 20:5 | 0.856 | 1.044 |
| LPC 18:3 | 0.855 | 1.044 |
| PE 40:5 PE 18:0-22:5 | 0.855 | 1.044 |
| PC 36:6 | 0.855 | 1.042 |
| LPE 18:2 A | 0.854 | 1.041 |
| LPC 16:1 (2) | 0.852 | 1.037 |
| SM d42:2 B | 0.852 | 1.036 |
| LPC 18:2 A | 0.851 | 1.034 |
| LPC 20:2 | 0.851 | 1.033 |
| PC 34:3 C | 0.849 | 1.031 |
| CL 74:9 | 0.848 | 1.029 |
| LPC 17:1 | 0.848 | 1.028 |
| LPC 18:2 | 0.848 | 1.027 |
| PC 34:1 (2) | 0.847 | 1.027 |
| PC 36:4 A | 0.845 | 1.023 |
| PE 40:7 PE 18:1-22:6 | 0.844 | 1.020 |
| PC 32:1 (2) | 0.842 | 1.016 |
| LPC 16:1 | 0.842 | 1.015 |
| PC 38:4 B | 0.841 | 1.012 |
| PC 36:5 A (2) | 0.840 | 1.010 |
| LPE 20:3 (2) | 0.839 | 1.009 |
| BMP 34:1 BMP 16:0-18:1 | 0.839 | 1.008 |
| PC 33:2 | 0.838 | 1.007 |
| PE 34:2 | 0.836 | 1.003 |
| LPE 20:3 | 0.834 | 0.999 |
| PE 40:6 A | 0.834 | 0.999 |
| SM d32:1 (2) | 0.832 | 0.994 |
| PE 40:8 B | 0.830 | 0.991 |
| PC 34:4 (2) | 0.829 | 0.988 |
| PC 40:6 A | 0.827 | 0.984 |
| PC 38:3 | 0.826 | 0.981 |
| PC 38:3 B | 0.825 | 0.979 |
| PE 40:7 A | 0.824 | 0.978 |
| PC 38:6 | 0.823 | 0.976 |
| LPC 18:2 B | 0.823 | 0.974 |
| PC 40:6 B | 0.820 | 0.969 |
| PE p-36:5 or PE o-36:6 | 0.820 | 0.969 |
| PC 36:4 C (2) | 0.820 | 0.968 |
| PC 37:6 | 0.819 | 0.966 |
| LPC 20:5 (2) | 0.819 | 0.966 |
| PC 36:3 B | 0.819 | 0.966 |
| PC 31:1 | 0.817 | 0.961 |
| LPC 20:2 2 | 0.816 | 0.960 |
| LPC 14:0-SN1 | 0.816 | 0.960 |
| ACar 18:2 | 0.816 | 0.959 |
| LPC 20:3 (2) | 0.814 | 0.955 |
| PC 38:7 | 0.813 | 0.954 |
| PE 33:2 PE 15:0-18:2 | 0.813 | 0.953 |
| PC 36:1 | 0.812 | 0.952 |
| PC 40:4 | 0.810 | 0.947 |
| PC 36:5 A | 0.809 | 0.945 |
| PE 36:2 (2) | 0.803 | 0.933 |
| LPC 22:5 (2) | 0.803 | 0.931 |
| SM d42:2 A | 0.802 | 0.930 |
| PC 38:6 C | 0.801 | 0.929 |
| PC 34:2 | 0.801 | 0.928 |
| PC 34:4 | 0.801 | 0.928 |
| PC 40:7 | 0.800 | 0.926 |
| PC 35:1 | 0.800 | 0.926 |
| SM d41:2 B | 0.800 | 0.925 |
| PC 40:7 A | 0.798 | 0.922 |
| LPC 18:1 | 0.798 | 0.921 |
| PE 36:4 PE 18:2-18:2 | 0.796 | 0.917 |
| PE 38:7 PE 16:1-22:6 | 0.794 | 0.912 |
| LPC 18:1 (2) | 0.793 | 0.912 |
| PC 33:1 | 0.792 | 0.909 |
| PE 36:4 | 0.792 | 0.908 |
| PC 36:1 (2) | 0.791 | 0.907 |
| PE 36:2 | 0.791 | 0.907 |
| PE 38:2 | 0.791 | 0.906 |
| PC p-34:1/PC o-34:2 | 0.790 | 0.904 |
| LPC 20:4 | 0.788 | 0.900 |
| CL 72:7 CL 18:1-18:2-18:2-18:2 | 0.780 | 0.883 |
| PC 35:4 (2) | 0.779 | 0.882 |
| PC 34:1 | 0.777 | 0.877 |
| PC p-34:2/PC o-34:3 | 0.773 | 0.868 |
| SM d42:2 | 0.771 | 0.863 |
| LPE 20:2 | 0.769 | 0.860 |
| LPC 20:3 | 0.768 | 0.858 |
| PE 38:6 (2) | 0.768 | 0.857 |
| PC 40:5 B | 0.767 | 0.857 |
| LPE 16:1 | 0.767 | 0.857 |
| LPE 22:6 (2) | 0.767 | 0.855 |
| CL 74:8 CL 18:1-18:2-18:2-20:3 | 0.766 | 0.854 |
| PC 36:3e | 0.766 | 0.853 |
| LPE 22:6 | 0.758 | 0.837 |
| PE 38:6 | 0.758 | 0.837 |
| LPC 15:0 | 0.754 | 0.829 |
| PC 40:8 (2) | 0.753 | 0.825 |
| LPC 20:1 | 0.749 | 0.816 |
| LPE 20:2 (2) | 0.748 | 0.816 |
| PI 40:6 | 0.747 | 0.813 |
| LPE 20:4 | 0.745 | 0.809 |
| PE 37:6 PE 15:0-22:6 | 0.743 | 0.805 |
| PE 35:2 PE 17:0-18:2 | 0.743 | 0.805 |
| PC p-36:1/PC o-36:2 A | 0.742 | 0.803 |
| ACar 20:4 | 0.740 | 0.798 |
| PC 32:1 | 0.736 | 0.791 |
| PC 33:0 | 0.726 | 0.768 |
| LPE 20:4 A | 0.723 | 0.763 |
| SM d40:2 B (2) | 0.722 | 0.760 |
| PI 40:7 | 0.720 | 0.755 |
| CL 72:6 CL 36:3-36:3 | 0.719 | 0.754 |
| PE 37:4 | 0.717 | 0.749 |
| PC 35:4 | 0.716 | 0.747 |
| SM d32:1 | 0.712 | 0.739 |
| PC 38:4 C | 0.712 | 0.739 |
| SM d40:2 A (2) | 0.698 | 0.709 |
| PC 36:4 B | 0.696 | 0.704 |
| SM d41:2 A (2) | 0.689 | 0.690 |
| CL 70:6 CL 34:3-36:3 | 0.688 | 0.688 |
| PC 39:4 | 0.687 | 0.685 |
| LPE 18:1 (2) | 0.686 | 0.684 |
| PC p-34:2 or PC o-34:3 | 0.686 | 0.682 |
| SM d42:1 (2) | 0.685 | 0.682 |
| PE 37:4 PE 17:0-20:4 | 0.685 | 0.682 |
| PC p-38:4/PC o-38:5 A | 0.683 | 0.676 |
| SM d41:2 A | 0.680 | 0.670 |
| PC 30:0 | 0.678 | 0.667 |
| PE 38:4 B | 0.671 | 0.650 |
| SM d40:1 (2) | 0.669 | 0.647 |
| GlcCer d42:2 | 0.666 | 0.641 |
| LNAPS 36:4 LNAPS 16:0/n-20:4 | 0.662 | 0.633 |
| LPC 22:4 | 0.661 | 0.631 |
| PC p-36:3 or PC o-36:4 2 | 0.651 | 0.609 |
| CL 72:8 CL 18:2-18:2-18:2-18:2 | 0.644 | 0.594 |
| PG 34:2 PG 16:0-18:2 | 0.643 | 0.592 |
| PC 38:4 A | 0.641 | 0.586 |
| LPE 20:4 B | 0.633 | 0.570 |
| SM d44:1 | 0.630 | 0.565 |
| LPE 18:2 B | 0.630 | 0.564 |
| LNAPS 36:2 LNAPS 17:2/n-19:0 | 0.627 | 0.559 |
| SM d40:1 | 0.627 | 0.557 |
| SM d33:1 (2) | 0.623 | 0.549 |
| PC p-34:0 or PC o-34:1 | 0.622 | 0.547 |
| SM d42:3 (2) | 0.620 | 0.542 |
| SM d42:1 | 0.618 | 0.539 |
| SM d43:1 B | 0.610 | 0.521 |
| PE 38:4 A | 0.608 | 0.518 |
| LPE 18:2 | 0.603 | 0.507 |
| GlcCer d42:1 | 0.600 | 0.500 |
| PE 39:4 | 0.599 | 0.497 |
| PE 36:1 | 0.598 | 0.496 |
| PC 31:0 | 0.596 | 0.491 |
| PC p-38:3/PC o-38:4 B | 0.594 | 0.488 |
| PC 38:1 | 0.592 | 0.483 |
| PC 37:4 | 0.589 | 0.478 |
| LPC 16:0 B | 0.588 | 0.474 |
| LPC 16:0 | 0.584 | 0.467 |
| PE 39:6 PE 17:0-22:6 | 0.579 | 0.455 |
| LNAPS 40:6 LNAPS 18:0/n-22:6 | 0.579 | 0.455 |
| SM d42:0 | 0.578 | 0.454 |
| CL 70:5 | 0.578 | 0.453 |
| PC p-38:4 or PC o-38:5 A | 0.578 | 0.452 |
| SM d41:1 (2) | 0.576 | 0.449 |
| LNAPS 38:4 LNAPS 17:2/n-21:2 | 0.575 | 0.448 |
| LNAPS 40:5 LNAPS 18:0/n-22:5 | 0.573 | 0.444 |
| LPC 16:0 A | 0.573 | 0.443 |
| PC p-40:3/PC o-40:4 | 0.573 | 0.442 |
| PE p-38:3 or PE o-38:4 | 0.569 | 0.435 |
| SM d40:2 B | 0.569 | 0.433 |
| PC p-36:4/PC o-36:5 | 0.567 | 0.430 |
| PE 38:5 B | 0.563 | 0.422 |
| GlcCer d41:1 | 0.560 | 0.415 |
| LNAPS 38:5 LNAPS 18:0/n-20:5 | 0.559 | 0.413 |
| PC 36:4 C | 0.556 | 0.407 |
| PC p-38:4/PC o-38:5 B | 0.556 | 0.406 |
| CL 70:4 CL 34:1-36:3 | 0.551 | 0.396 |
| PC p-38:3 or PC o-38:4 | 0.549 | 0.392 |
| PG 36:3 PG 18:1-18:2 | 0.545 | 0.383 |
| PE 38:4e PE 18:0e/20:4 | 0.544 | 0.381 |
| PE p-40:4 or PE o-40:5 | 0.531 | 0.353 |
| CL 68:2 | 0.526 | 0.342 |
| SM d34:2 (2) | 0.525 | 0.340 |
| LPE 20:1 | 0.522 | 0.335 |
| SM d40:2 A | 0.520 | 0.330 |
| PC p-38:5/PC o-38:6 A | 0.509 | 0.306 |
| PC p-36:2 or PC o-36:3 | 0.508 | 0.304 |
| PE 40:4 PE 18:0-22:4 | 0.506 | 0.300 |
| LNAPS 38:6 | 0.494 | 0.274 |
| GlcCer d40:1 | 0.493 | 0.272 |
| GlcCer d42:2 (2) | 0.492 | 0.270 |
| PC p-36:3 or PC o-36:4 | 0.490 | 0.267 |
| SM d44:2 | 0.490 | 0.267 |
| PC p-32:0 or PC o-32:1 2 | 0.488 | 0.263 |
| PC p-34:1 or PC o-34:2 A | 0.487 | 0.261 |
| SM d34:2 | 0.479 | 0.243 |
| PE p-38:5 or PE o-38:6 | 0.464 | 0.212 |
| SM d42:3 | 0.452 | 0.185 |
| SM d43:1 A | 0.433 | 0.146 |
| PC p-38:4 or PC o-38:5 B | 0.424 | 0.126 |
| PG 36:4 PG 18:2-18:2 | 0.412 | 0.099 |
| PC o-32:0 (2) | 0.407 | 0.089 |
| SM d34:0 (2) | 0.398 | 0.071 |
| PI 40:8 PI 20:4-20:4 | 0.397 | 0.069 |
| SM d43:1 | 0.396 | 0.066 |
| DAG 40:6 DAG 18:2-22:4 | 0.374 | 0.018 |
| DG 34:2 | 0.370 | 0.010 |
| DAG 38:4 DAG 18:2-20:2 | 0.367 | 0.005 |
| SM d34:1 (2) | 0.367 | 0.004 |
| PC 40:8 | 0.363 | -0.003 |
| DAG 38:3 DAG 20:1-18:2 | 0.336 | -0.061 |
| DAG 32:1 DAG 14:0-18:1 | 0.331 | -0.072 |
| SM d40:0 | 0.329 | -0.077 |
| FA 30:1 | 0.328 | -0.079 |
| PE p-38:4 or PE o-38:5 | 0.327 | -0.082 |
| GlcCer d40:1 (2) | 0.319 | -0.099 |
| PC p-42:3 or PC o-42:4 | 0.318 | -0.100 |
| DG 34:1 | 0.314 | -0.108 |
| DG 38:6 | 0.311 | -0.114 |
| DG 38:5 | 0.310 | -0.116 |
| PE 40:8 A | 0.309 | -0.118 |
| DAG 32:2 DAG 16:1-16:1 | 0.300 | -0.138 |
| PE p-36:2 or PE o-36:3 | 0.300 | -0.138 |
| Cer-NS d42:1 Cer-NS d18:1/24:0 A | 0.287 | -0.166 |
| PE p-36:4 or PE o-36:5 | 0.283 | -0.174 |
| DG 36:4 B | 0.281 | -0.178 |
| SM d39:1 (2) | 0.281 | -0.179 |
| DAG 35:2 DAG 17:1-18:1 | 0.279 | -0.184 |
| SM d39:1 | 0.273 | -0.195 |
| LPC 18:0 | 0.268 | -0.206 |
| Cer-NS d43:1 Cer-NS d18:1/25:0 | 0.267 | -0.208 |
| DG 36:2 | 0.266 | -0.210 |
| SM d33:1 | 0.265 | -0.213 |
| DG 36:4 A | 0.265 | -0.214 |
| PC o-34:0 | 0.264 | -0.216 |
| FA 34:1 | 0.263 | -0.218 |
| PE p-34:2 or PE o-34:3 | 0.262 | -0.219 |
| SM d38:2 (2) | 0.253 | -0.239 |
| FA 32:1 | 0.252 | -0.241 |
| DG 36:3 | 0.248 | -0.248 |
| SMd30:1 | 0.227 | -0.293 |
| LPE 16:0 B | 0.223 | -0.302 |
| DG 36:5 | 0.223 | -0.302 |
| DG 34:3 | 0.220 | -0.309 |
| DAG 32:0 DAG 16:0-16:0 | 0.219 | -0.310 |
| FA 20:5 (eicosapentaenoic acid) | 0.211 | -0.329 |
| LPC 18:0 B | 0.207 | -0.336 |
| DG 36:1 | 0.203 | -0.345 |
| PE 36:5 B | 0.202 | -0.348 |
| PC 18:0e | 0.196 | -0.359 |
| PE p-34:1 or PE o-34:2 | 0.195 | -0.362 |
| TAG 56:6 TAG 18:1-18:1-20:4 | 0.192 | -0.368 |
| CholesterolSulfate | 0.192 | -0.368 |
| FA 14:1 (physeteric acid) | 0.185 | -0.383 |
| LPE 16:0 | 0.185 | -0.383 |
| TAG 48:0 | 0.184 | -0.385 |
| SM d38:0 | 0.177 | -0.400 |
| TAG 52:2 TAG 16:0-18:1-18:1 | 0.170 | -0.414 |
| FA 12:0 (lauric acid) | 0.169 | -0.417 |
| FA 26:1 | 0.168 | -0.419 |
| SM d36:1 (2) | 0.164 | -0.427 |
| Cer-NS d42:1 Cer-NS d18:1/24:0 B | 0.150 | -0.457 |
| FA 20:5 | 0.143 | -0.473 |
| Ceramide d42:1 | 0.135 | -0.489 |
| PC o-32:0 | 0.134 | -0.492 |
| SM d36:2 (2) | 0.130 | -0.501 |
| FA 18:4 | 0.128 | -0.504 |
| FA 15:1 A | 0.118 | -0.525 |
| TAG 50:2 | 0.111 | -0.541 |
| Cer-NS d43:1 Cer-NS d18:1/25:0 (2) | 0.103 | -0.558 |
| TAG 53:2 | 0.091 | -0.583 |
| Ceramide d40:0 | 0.088 | -0.589 |
| TAG 53:3 TAG 17:1-17:1-19:1 | 0.085 | -0.596 |
| SM d38:1 (2) | 0.083 | -0.601 |
| CE 18:3 | 0.081 | -0.604 |
| TAG 56:9 TAG 18:2-18:2-20:5 | 0.081 | -0.605 |
| TAG 54:2 | 0.079 | -0.609 |
| TAG 52:1 | 0.071 | -0.626 |
| LPC 20:0 | 0.069 | -0.629 |
| SM d34:1 | 0.068 | -0.632 |
| DG 36:6 | 0.067 | -0.634 |
| AC 18:0 | 0.064 | -0.641 |
| FA 16:1 (palmitoleic acid) | 0.059 | -0.651 |
| FA 14:0 (myristic acid) | 0.059 | -0.652 |
| TAG 51:3 | 0.047 | -0.678 |
| CE 18:1 | 0.044 | -0.684 |
| TAG 52:5 TAG 16:0-16:0-20:5 | 0.043 | -0.685 |
| FA 17:2 | 0.042 | -0.688 |
| Ceramide d43:1 | 0.041 | -0.689 |
| TAG 54:3 TAG 16:0-18:0-20:3 | 0.039 | -0.695 |
| Ceramide d41:1 | 0.038 | -0.695 |
| TAG 56:3 TAG 18:1-18:1-20:1 | 0.034 | -0.703 |
| SM d38:1 | 0.034 | -0.704 |
| FA 15:1 B | 0.032 | -0.710 |
| FA 18:3 (linolenic acid) | 0.031 | -0.711 |
| Cer-NS d41:1 Cer-NS d18:1/23:0 A | 0.022 | -0.729 |
| FA 16:3 | 0.018 | -0.738 |
| TAG 56:8 TAG 16:0-18:2-22:6 | 0.018 | -0.739 |
| DAG 36:4e DAG 19:2e/17:2 | 0.014 | -0.747 |
| TAG 54:3 TAG 16:0-18:1-20:2 | 0.008 | -0.761 |
| TAG 48:3 | 0.006 | -0.764 |
| Cer d42:1 | -0.006 | -0.789 |
| Cer-NS d44:2 Cer-NS d18:1/26:1 | -0.007 | -0.791 |
| FA 17:1 | -0.013 | -0.805 |
| TAG 52:4 TAG 16:0-18:2-18:2 | -0.014 | -0.808 |
| Cer d41:1 | -0.022 | -0.823 |
| PE p-36:1 or PE o-36:2 | -0.026 | -0.832 |
| LPC 19:0-SN1 | -0.029 | -0.839 |
| CE 22:6 | -0.034 | -0.849 |
| TAG 50:4 TAG 16:1-16:1-18:2 | -0.036 | -0.853 |
| FA 16:2 | -0.042 | -0.866 |
| TAG 60:7 | -0.049 | -0.881 |
| LPE 17:0 | -0.055 | -0.894 |
| FAHFA 32:0 FAHFA 16:0/16:0 | -0.061 | -0.907 |
| CE 18:2 | -0.065 | -0.916 |
| FA 15:0 (pentadecylic acid) | -0.066 | -0.918 |
| PC 32:0 (2) | -0.067 | -0.919 |
| DAG 34:3e DAG 19:2e/15:1 | -0.070 | -0.926 |
| TAG 52:7 TAG 16:1-16:1-20:5 | -0.071 | -0.928 |
| CE 20:5 | -0.074 | -0.935 |
| PC p-38:6/PC o-38:7 | -0.076 | -0.938 |
| Ceramide d39:1 | -0.078 | -0.943 |
| FA 22:3 | -0.081 | -0.949 |
| Cer d40:1 | -0.091 | -0.970 |
| Cer-NS d41:2 Cer-NS d18:2/23:0 | -0.091 | -0.971 |
| FA 20:2 | -0.104 | -0.997 |
| TAG 55:3 | -0.106 | -1.003 |
| FA 24:5 | -0.109 | -1.008 |
| LPE 17:0 (2) | -0.110 | -1.011 |
| TAG 60:12 TAG 20:4-20:4-20:4 | -0.118 | -1.027 |
| Cer-NS d41:2 Cer-NS d26:2/15:0 | -0.118 | -1.028 |
| FA 18:1 (oleic acid) | -0.119 | -1.031 |
| LPE 19:0 | -0.122 | -1.036 |
| Ceramide d42:2 B | -0.123 | -1.039 |
| FA 20:3 (eicosatrienoic acid) | -0.124 | -1.041 |
| Ceramide d40:1 | -0.126 | -1.044 |
| Cer-NS d42:2 Cer-NS d18:1/24:1 | -0.128 | -1.049 |
| TAG 53:4 | -0.135 | -1.065 |
| TAG 50:5 | -0.140 | -1.075 |
| TAG 54:6 | -0.143 | -1.082 |
| Cer-NS d40:1 Cer-NS d18:1/22:0 | -0.145 | -1.086 |
| Ceramide d42:2 A | -0.151 | -1.098 |
| FA 22:5 | -0.152 | -1.101 |
| PC 32:0 | -0.161 | -1.118 |
| FA 22:2 | -0.161 | -1.120 |
| FA 23:0 | -0.162 | -1.121 |
| Ceramide d44:1 | -0.168 | -1.133 |
| Cer d42:2 B | -0.171 | -1.142 |
| PE p-40:7 or PE o-40:8 | -0.183 | -1.166 |
| FA 24:6 | -0.183 | -1.167 |
| Cer d42:2 A | -0.189 | -1.180 |
| Cer-NS d41:1 Cer-NS d18:1/23:0 B | -0.190 | -1.181 |
| TAG 48:4 B | -0.201 | -1.204 |
| Cer-NS d43:2 Cer-NS d20:2/23:0 | -0.220 | -1.245 |
| Cer-NS d42:3 Cer-NS d18:1/24:2 | -0.230 | -1.267 |
| LPC 18:0 A | -0.250 | -1.309 |
| LPE 16:0 A | -0.253 | -1.314 |
| DAG 36:0 DAG 18:0-18:0 | -0.254 | -1.316 |
| FA 24:1 | -0.254 | -1.318 |
| Cer-NS d42:3 Cer-NS d18:2/24:1 | -0.258 | -1.326 |
| FA 20:3 (homo-gamma-linolenic acid) | -0.264 | -1.338 |
| FAHFA 18:0 FAHFA 7:0/11:0 | -0.268 | -1.347 |
| FA 21:1 | -0.275 | -1.362 |
| PC p-32:0 or PC o-32:1 | -0.275 | -1.362 |
| FA 18:1 | -0.285 | -1.383 |
| FA 18:2 (linoleic acid) | -0.289 | -1.392 |
| SM d36:0 | -0.297 | -1.408 |
| FA 24:2 | -0.298 | -1.411 |
| Cer-NS d40:2 Cer-NS d18:1/22:1 | -0.299 | -1.412 |
| PE 40:9 | -0.302 | -1.420 |
| Ceramide d33:1 | -0.303 | -1.421 |
| FA 16:0 (palmitic acid) | -0.303 | -1.422 |
| FA 22:1 | -0.304 | -1.425 |
| FA 19:1 | -0.305 | -1.426 |
| FA 21:0 | -0.309 | -1.435 |
| FA 20:2 (eicosadienoic acid) | -0.310 | -1.437 |
| Ceramide d34:0 | -0.312 | -1.442 |
| SM d36:1 | -0.319 | -1.456 |
| Ceramide d40:2 | -0.320 | -1.457 |
| FA 22:6 (docosahexaenoic acid) | -0.321 | -1.459 |
| Ceramide d32:1 | -0.338 | -1.497 |
| LPE 18:0 (2) | -0.340 | -1.501 |
| Ceramide d38:1 | -0.345 | -1.511 |
| Cer d38:1 | -0.357 | -1.537 |
| Cer-NS d38:2 Cer-NS d18:2/20:0 | -0.376 | -1.577 |
| Gal-Gal-Cer d18:1/16:0 or Lactosylceramide d18:1/16:0 | -0.377 | -1.579 |
| FA 20:0 | -0.385 | -1.597 |
| PE 40:8 PE 20:4-20:4 | -0.389 | -1.604 |
| FA 20:1 | -0.403 | -1.635 |
| Cer-NS d34:1 Cer-NS d18:1/16:0 | -0.412 | -1.654 |
| PE 38:7 | -0.413 | -1.656 |
| PE p-38:6 or PE o-38:7 | -0.415 | -1.660 |
| Ceramide d36:1 | -0.421 | -1.673 |
| PE p-40:6 or PE o-40:7 | -0.422 | -1.676 |
| PC p-36:2/PC o-36:3 | -0.426 | -1.684 |
| LPE 20:0 | -0.429 | -1.690 |
| Cer d34:1 | -0.433 | -1.698 |
| Cer d36:1 | -0.434 | -1.701 |
| Ceramide d34:1 | -0.436 | -1.705 |
| Cer-NS d38:1 Cer-NS d18:1/20:0 | -0.437 | -1.707 |
| FA 17:0 (margaric acid) | -0.446 | -1.727 |
| PI 38:4 | -0.447 | -1.728 |
| PC p-38:5 or PC o-38:6 | -0.457 | -1.750 |
| PC 34:0 (2) | -0.478 | -1.795 |
| CE 20:4 | -0.480 | -1.799 |
| PG 40:8 PG 18:2-22:6 | -0.491 | -1.821 |
| PE 40:6 B | -0.495 | -1.831 |
| LPE 19:0 (2) | -0.503 | -1.847 |
| PC 34:0 | -0.504 | -1.849 |
| FA 20:4 (arachidonic acid) | -0.519 | -1.882 |
| FA 20:0 (arachidic acid) | -0.537 | -1.920 |
| FA 19:0 | -0.537 | -1.920 |
| LPC 14:0 (2) | -0.543 | -1.933 |
| LPE 18:2e | -0.549 | -1.946 |
| LPC p-18:0 or LPC o-18:1 | -0.552 | -1.952 |
| FA 24:4 | -0.570 | -1.991 |
| LPE 18:0 | -0.575 | -2.000 |
| Cer-NS d36:2 Cer-NS d20:1/16:1 | -0.591 | -2.035 |
| LPC o-16:0 | -0.606 | -2.066 |
| LPC p-16:0 or LPC o-16:1 | -0.621 | -2.098 |
| SM d34:0 | -0.623 | -2.103 |
| LPE 16:1e | -0.643 | -2.146 |
| LPE 18:1e (2) | -0.670 | -2.204 |
| LPE 18:1e | -0.674 | -2.210 |
| Ceramide d34:2 | -0.697 | -2.260 |
| Cholesterol | -0.751 | -2.374 |
| PE 40:7 B | -0.770 | -2.416 |
| PE 35:0 PE 17:0-18:0 | -0.775 | -2.427 |

**Table S4**. PCA 2

| **Lipids** | **PC2 Loadings** | **PC2_Zscore** |
| --- | --- | --- |
| Ceramide d39:1 | 0.899 | 2.066 |
| Cer-NS d41:2 Cer-NS d26:2/15:0 | 0.898 | 2.061 |
| Ceramide d42:2 B | 0.885 | 2.030 |
| Cer-NS d42:3 Cer-NS d18:2/24:1 | 0.879 | 2.014 |
| Cer-NS d41:2 Cer-NS d18:2/23:0 | 0.860 | 1.968 |
| Cer-NS d42:3 Cer-NS d18:1/24:2 | 0.856 | 1.957 |
| Ceramide d43:1 | 0.853 | 1.951 |
| Cer d42:2 B | 0.845 | 1.932 |
| Ceramide d41:1 | 0.842 | 1.924 |
| SM d39:1 (2) | 0.822 | 1.874 |
| Cer d42:2 A | 0.820 | 1.869 |
| Ceramide d42:2 A | 0.817 | 1.863 |
| Cer-NS d41:1 Cer-NS d18:1/23:0 A | 0.811 | 1.848 |
| Ceramide d40:2 | 0.808 | 1.839 |
| Cer d41:1 | 0.806 | 1.836 |
| Cer-NS d42:2 Cer-NS d18:1/24:1 | 0.787 | 1.787 |
| FA 24:1 | 0.777 | 1.765 |
| Cer-NS d44:2 Cer-NS d18:1/26:1 | 0.771 | 1.750 |
| Cer d40:1 | 0.769 | 1.743 |
| FA 24:2 | 0.767 | 1.739 |
| Cer d42:1 | 0.758 | 1.717 |
| FA 23:0 | 0.755 | 1.710 |
| Ceramide d42:1 | 0.753 | 1.704 |
| Cer-NS d40:1 Cer-NS d18:1/22:0 | 0.749 | 1.695 |
| SM d39:1 | 0.740 | 1.672 |
| Ceramide d40:1 | 0.737 | 1.665 |
| Ceramide d33:1 | 0.730 | 1.648 |
| Cer-NS d42:1 Cer-NS d18:1/24:0 B | 0.725 | 1.635 |
| FA 21:0 | 0.703 | 1.581 |
| Gal-Gal-Cer d18:1/16:0 or Lactosylceramide d18:1/16:0 | 0.701 | 1.576 |
| LPC 14:0 (2) | 0.688 | 1.543 |
| Cer d38:1 | 0.686 | 1.540 |
| SM d43:1 B | 0.672 | 1.504 |
| SM d41:1 (2) | 0.670 | 1.499 |
| Ceramide d32:1 | 0.669 | 1.498 |
| PE 40:8 PE 20:4-20:4 | 0.650 | 1.451 |
| Ceramide d38:1 | 0.650 | 1.450 |
| PC 40:8 | 0.621 | 1.380 |
| Cer d36:1 | 0.619 | 1.375 |
| PG 40:8 PG 18:2-22:6 | 0.617 | 1.370 |
| CholesterolSulfate | 0.604 | 1.338 |
| FA 26:1 | 0.602 | 1.332 |
| Cer-NS d34:1 Cer-NS d18:1/16:0 | 0.599 | 1.325 |
| FA 20:0 | 0.599 | 1.325 |
| Ceramide d34:1 | 0.592 | 1.308 |
| Cer-NS d43:2 Cer-NS d20:2/23:0 | 0.583 | 1.284 |
| SM d42:1 | 0.560 | 1.228 |
| PE 36:1 | 0.553 | 1.212 |
| LPE 20:0 | 0.552 | 1.210 |
| SM d42:1 (2) | 0.549 | 1.202 |
| Cer d34:1 | 0.549 | 1.200 |
| Ceramide d36:1 | 0.548 | 1.198 |
| FA 17:0 (margaric acid) | 0.546 | 1.193 |
| SM d38:1 | 0.534 | 1.164 |
| SM d41:2 A | 0.525 | 1.143 |
| FAHFA 32:0 FAHFA 16:0/16:0 | 0.513 | 1.112 |
| PE 40:6 B | 0.510 | 1.105 |
| LPE 19:0 (2) | 0.509 | 1.103 |
| FA 24:4 | 0.506 | 1.096 |
| SM d40:1 (2) | 0.497 | 1.074 |
| SM d41:2 A (2) | 0.497 | 1.072 |
| PI 40:8 PI 20:4-20:4 | 0.491 | 1.058 |
| FA 22:1 | 0.486 | 1.045 |
| FA 24:5 | 0.485 | 1.044 |
| PC 34:0 (2) | 0.482 | 1.037 |
| PC 31:0 | 0.477 | 1.024 |
| PG 36:4 PG 18:2-18:2 | 0.473 | 1.013 |
| PE 39:6 PE 17:0-22:6 | 0.467 | 1.000 |
| PC 34:0 | 0.464 | 0.992 |
| FA 20:4 (arachidonic acid) | 0.464 | 0.991 |
| Ceramide d44:1 | 0.462 | 0.988 |
| Cer-NS d41:1 Cer-NS d18:1/23:0 B | 0.454 | 0.967 |
| SM d38:1 (2) | 0.453 | 0.966 |
| Cer-NS d40:2 Cer-NS d18:1/22:1 | 0.447 | 0.949 |
| Cer-NS d38:2 Cer-NS d18:2/20:0 | 0.440 | 0.932 |
| Cer-NS d36:2 Cer-NS d20:1/16:1 | 0.437 | 0.926 |
| SM d41:2 B | 0.435 | 0.921 |
| SMd30:1 | 0.431 | 0.912 |
| FA 20:2 (eicosadienoic acid) | 0.424 | 0.894 |
| LPC 19:0-SN1 | 0.422 | 0.888 |
| Ceramide d34:0 | 0.409 | 0.856 |
| PE 36:4 PE 18:2-18:2 | 0.408 | 0.853 |
| FA 30:1 | 0.406 | 0.849 |
| FAHFA 18:0 FAHFA 7:0/11:0 | 0.399 | 0.832 |
| Cer-NS d38:1 Cer-NS d18:1/20:0 | 0.396 | 0.823 |
| SM d40:1 | 0.395 | 0.823 |
| PE 35:0 PE 17:0-18:0 | 0.393 | 0.817 |
| FA 20:0 (arachidic acid) | 0.391 | 0.812 |
| SM d44:2 | 0.388 | 0.805 |
| PC p-32:0 or PC o-32:1 | 0.380 | 0.785 |
| PC 40:8 (2) | 0.377 | 0.778 |
| PC 37:6 | 0.373 | 0.768 |
| LPC 20:0 | 0.370 | 0.759 |
| FA 22:6 (docosahexaenoic acid) | 0.365 | 0.749 |
| SM d42:2 B | 0.363 | 0.743 |
| SM d44:1 | 0.361 | 0.738 |
| SM d42:2 | 0.356 | 0.727 |
| PG 36:3 PG 18:1-18:2 | 0.354 | 0.720 |
| LPE 18:0 | 0.349 | 0.708 |
| PC p-36:2 or PC o-36:3 | 0.346 | 0.702 |
| PC p-34:1 or PC o-34:2 A | 0.337 | 0.679 |
| PC o-32:0 | 0.336 | 0.677 |
| SM d41:2B 2 | 0.333 | 0.670 |
| PE 40:5 PE 18:0-22:5 | 0.331 | 0.663 |
| PC 38:1 | 0.326 | 0.651 |
| SM d33:1 | 0.324 | 0.648 |
| PC 36:3e | 0.324 | 0.647 |
| PC 34:4 (2) | 0.324 | 0.646 |
| PE 35:2 PE 17:0-18:2 | 0.324 | 0.646 |
| FA 32:1 | 0.318 | 0.632 |
| LPE 17:0 | 0.316 | 0.626 |
| LPC p-18:0 or LPC o-18:1 | 0.311 | 0.615 |
| PE 33:2 PE 15:0-18:2 | 0.310 | 0.612 |
| FA 20:1 | 0.309 | 0.610 |
| SM d42:3 (2) | 0.309 | 0.609 |
| Ceramide d34:2 | 0.308 | 0.608 |
| SM d42:3 | 0.308 | 0.607 |
| FA 34:1 | 0.301 | 0.589 |
| PE 38:6 (2) | 0.295 | 0.575 |
| PE 36:2 (2) | 0.295 | 0.574 |
| PC 34:4 | 0.290 | 0.564 |
| FA 20:3 (homo-gamma-linolenic acid) | 0.290 | 0.563 |
| PG 34:2 PG 16:0-18:2 | 0.290 | 0.562 |
| PE 36:2 | 0.287 | 0.554 |
| SM d36:1 (2) | 0.286 | 0.553 |
| LPE 17:0 (2) | 0.278 | 0.533 |
| PE 38:5 B | 0.277 | 0.530 |
| Ceramide d40:0 | 0.272 | 0.519 |
| PC 36:6 | 0.270 | 0.515 |
| PC 35:4 (2) | 0.270 | 0.513 |
| PE 34:1 | 0.268 | 0.510 |
| PE 40:7 PE 18:1-22:6 | 0.267 | 0.507 |
| PC p-36:1/PC o-36:2 A | 0.265 | 0.501 |
| PC 30:0 | 0.264 | 0.500 |
| PC 35:4 | 0.264 | 0.498 |
| LPE 22:6 | 0.262 | 0.494 |
| FA 22:2 | 0.261 | 0.492 |
| PE 40:7 A | 0.261 | 0.492 |
| PC 37:2 | 0.261 | 0.491 |
| SM d42:2 A | 0.260 | 0.490 |
| PC 37:2 (2) | 0.260 | 0.489 |
| PE 36:3 A | 0.258 | 0.485 |
| PC 42:6 | 0.258 | 0.484 |
| PC 32:0 | 0.254 | 0.473 |
| PC 38:6 A | 0.251 | 0.466 |
| PC p-38:5/PC o-38:6 A | 0.249 | 0.461 |
| LPC 20:5 | 0.248 | 0.460 |
| LPE 16:0 A | 0.246 | 0.454 |
| PC 36:4 A | 0.245 | 0.451 |
| PE 36:3 | 0.244 | 0.448 |
| PE 37:6 PE 15:0-22:6 | 0.242 | 0.446 |
| LPE 18:2 | 0.240 | 0.440 |
| FA 19:0 | 0.238 | 0.436 |
| FA 21:1 | 0.237 | 0.433 |
| LPE 22:6 (2) | 0.234 | 0.424 |
| PC p-36:2/PC o-36:3 | 0.228 | 0.411 |
| LPC 15:0 | 0.223 | 0.398 |
| DAG 36:0 DAG 18:0-18:0 | 0.222 | 0.396 |
| PC 33:2 | 0.222 | 0.395 |
| PC 33:2 (2) | 0.221 | 0.392 |
| PE 40:9 | 0.219 | 0.388 |
| PC o-32:0 (2) | 0.219 | 0.388 |
| LPC 16:0 A | 0.219 | 0.387 |
| LPC 18:0 A | 0.217 | 0.384 |
| LPC o-16:0 | 0.213 | 0.374 |
| LPE 18:0 (2) | 0.210 | 0.365 |
| LPE 18:2 B | 0.207 | 0.357 |
| GlcCer d41:1 | 0.206 | 0.355 |
| PE 40:4 PE 18:0-22:4 | 0.205 | 0.353 |
| PC 36:4 A (2) | 0.202 | 0.347 |
| PC 38:6 B | 0.198 | 0.337 |
| PI 36:4 | 0.197 | 0.334 |
| Cholesterol | 0.197 | 0.333 |
| PC 35:2 B | 0.195 | 0.328 |
| FA 16:3 | 0.194 | 0.326 |
| PE 38:7 | 0.186 | 0.307 |
| LPC 22:6 | 0.185 | 0.304 |
| LPC 22:6 2 | 0.183 | 0.299 |
| PC 38:7 | 0.182 | 0.297 |
| PC 32:0 (2) | 0.176 | 0.282 |
| PC 36:5 A (2) | 0.174 | 0.276 |
| LPC 14:0-SN1 | 0.174 | 0.276 |
| PC 36:5 A | 0.172 | 0.272 |
| FA 16:0 (palmitic acid) | 0.171 | 0.269 |
| SM d42:0 | 0.166 | 0.256 |
| PE 37:4 | 0.165 | 0.254 |
| SM d36:1 | 0.163 | 0.251 |
| PC 39:4 | 0.162 | 0.248 |
| LPC 20:4 | 0.161 | 0.244 |
| PC p-34:1/PC o-34:2 | 0.159 | 0.239 |
| PC 34:3 | 0.157 | 0.236 |
| PC 38:3 A | 0.157 | 0.234 |
| PC 40:7 B | 0.155 | 0.230 |
| FA 16:2 | 0.155 | 0.229 |
| PC p-38:4 or PC o-38:5 B | 0.155 | 0.229 |
| LPE 18:1 | 0.153 | 0.225 |
| LPE 22:5 | 0.153 | 0.225 |
| PE 40:6 A | 0.153 | 0.225 |
| LPC 18:2 A | 0.152 | 0.222 |
| GlcCer d40:1 (2) | 0.150 | 0.219 |
| FA 19:1 | 0.147 | 0.210 |
| LPE 18:2 A | 0.147 | 0.209 |
| LPC 18:2 | 0.147 | 0.209 |
| LPC 18:3 | 0.145 | 0.205 |
| PC p-34:2/PC o-34:3 | 0.145 | 0.205 |
| PC 40:5 B | 0.143 | 0.200 |
| PC 40:7 | 0.142 | 0.197 |
| LPC 22:5 (2) | 0.137 | 0.185 |
| PC 32:2 (2) | 0.136 | 0.184 |
| CL 72:8 CL 18:2-18:2-18:2-18:2 | 0.136 | 0.182 |
| PC 32:2 | 0.133 | 0.177 |
| CL 74:8 CL 18:1-18:2-18:2-20:3 | 0.132 | 0.173 |
| FA 22:5 | 0.130 | 0.169 |
| SM d40:2 B | 0.130 | 0.169 |
| PC 37:5 | 0.129 | 0.165 |
| LPE 20:2 (2) | 0.127 | 0.162 |
| PC p-32:0 or PC o-32:1 2 | 0.126 | 0.158 |
| LPC 14:0 | 0.124 | 0.155 |
| GlcCer d42:2 (2) | 0.123 | 0.152 |
| PC p-34:2 or PC o-34:3 | 0.120 | 0.144 |
| FA 22:3 | 0.118 | 0.138 |
| LPC 22:4 | 0.116 | 0.135 |
| PC 40:5 A | 0.116 | 0.133 |
| PC 40:6 A | 0.115 | 0.132 |
| PI 40:7 | 0.114 | 0.130 |
| PC 35:3 | 0.114 | 0.128 |
| PC p-38:5 or PC o-38:6 | 0.113 | 0.127 |
| LPC 18:2 B | 0.112 | 0.124 |
| LPE 18:1e | 0.107 | 0.111 |
| LPE 18:1e (2) | 0.106 | 0.110 |
| PE 38:5 PE 18:1-20:4 | 0.106 | 0.108 |
| LPE 20:1 | 0.104 | 0.104 |
| PC 36:5 C | 0.102 | 0.098 |
| PC 40:6 A (2) | 0.100 | 0.095 |
| FA 24:6 | 0.097 | 0.088 |
| LPC 22:5 | 0.093 | 0.077 |
| PC p-38:4/PC o-38:5 B | 0.093 | 0.076 |
| LPC 20:2 | 0.093 | 0.076 |
| PC 40:7 A | 0.085 | 0.058 |
| PE 37:4 PE 17:0-20:4 | 0.084 | 0.055 |
| PC 35:2 | 0.082 | 0.049 |
| FA 18:2 (linoleic acid) | 0.079 | 0.042 |
| Cer-NS d42:1 Cer-NS d18:1/24:0 A | 0.078 | 0.039 |
| PE 34:2 (2) | 0.077 | 0.038 |
| LPE 20:4 A | 0.076 | 0.035 |
| PC 38:4 B | 0.075 | 0.034 |
| FA 18:1 | 0.074 | 0.031 |
| LPE 20:2 | 0.074 | 0.029 |
| PI 38:5 | 0.073 | 0.028 |
| LPE 16:0 B | 0.073 | 0.027 |
| SM d33:1 (2) | 0.073 | 0.027 |
| PC 34:2 (2) | 0.073 | 0.027 |
| SM d40:2 A | 0.072 | 0.026 |
| PE 34:2 | 0.071 | 0.023 |
| PC 34:3 B | 0.071 | 0.023 |
| SM d36:2 (2) | 0.070 | 0.019 |
| SM d32:1 | 0.066 | 0.010 |
| LPC 20:3 | 0.065 | 0.009 |
| PE 40:7 B | 0.058 | -0.009 |
| PC 34:3 C | 0.058 | -0.010 |
| LPC 20:2 2 | 0.057 | -0.011 |
| PE p-40:4 or PE o-40:5 | 0.057 | -0.012 |
| PC p-38:4/PC o-38:5 A | 0.057 | -0.013 |
| LPC 18:0 B | 0.057 | -0.013 |
| LPE 20:4 B | 0.054 | -0.018 |
| PE 38:5 A | 0.054 | -0.018 |
| PC 38:2 | 0.054 | -0.018 |
| PC 36:4 C (2) | 0.053 | -0.021 |
| SM d40:2 A (2) | 0.053 | -0.022 |
| PC 39:6 | 0.052 | -0.023 |
| SM d40:2 B (2) | 0.051 | -0.027 |
| PC 40:5 A (2) | 0.050 | -0.028 |
| FA 20:5 | 0.050 | -0.028 |
| PE 34:3 PE 16:0-18:3 | 0.047 | -0.035 |
| PC 36:3 A (2) | 0.047 | -0.036 |
| LPC p-16:0 or LPC o-16:1 | 0.046 | -0.039 |
| PC 36:4 B | 0.045 | -0.040 |
| GlcCer d42:2 | 0.045 | -0.041 |
| CL 70:6 CL 34:3-36:3 | 0.041 | -0.051 |
| PC p-34:0 or PC o-34:1 | 0.040 | -0.053 |
| FA 20:3 (eicosatrienoic acid) | 0.039 | -0.055 |
| PC 38:2 (2) | 0.039 | -0.057 |
| PC 37:4 (2) | 0.037 | -0.061 |
| PC 36:5 D | 0.036 | -0.063 |
| PE 38:3 PE 18:0-20:3 | 0.035 | -0.066 |
| LPE 20:4 | 0.031 | -0.075 |
| PE 38:7 PE 16:1-22:6 | 0.031 | -0.075 |
| PC 18:0e | 0.030 | -0.079 |
| PI 36:3 PI 16:0-20:3 | 0.028 | -0.083 |
| SM d32:1 (2) | 0.027 | -0.086 |
| PE 40:8 B | 0.026 | -0.087 |
| PC p-40:3/PC o-40:4 | 0.026 | -0.088 |
| PC p-36:5/PC o-36:6 | 0.026 | -0.088 |
| PE 36:3 B | 0.025 | -0.091 |
| PC 40:4 (2) | 0.025 | -0.091 |
| LPE 20:3 | 0.023 | -0.095 |
| LPE 20:3 (2) | 0.021 | -0.100 |
| FA 12:0 (lauric acid) | 0.020 | -0.102 |
| LPE 16:1e | 0.017 | -0.110 |
| LPC 20:1 | 0.017 | -0.110 |
| LPE 16:0 | 0.017 | -0.111 |
| PC 35:2 A | 0.016 | -0.112 |
| PC 37:4 | 0.015 | -0.116 |
| FA 15:0 (pentadecylic acid) | 0.015 | -0.116 |
| PC 40:4 | 0.013 | -0.120 |
| PC 38:5 A (2) | 0.012 | -0.123 |
| PC 35:1 | 0.010 | -0.127 |
| PE 38:4 B | 0.010 | -0.128 |
| SM d38:2 (2) | 0.009 | -0.131 |
| PC p-42:3 or PC o-42:4 | 0.005 | -0.140 |
| PC 36:5 B | 0.004 | -0.143 |
| SM d34:1 | -0.001 | -0.154 |
| PE 38:4 A | -0.001 | -0.155 |
| GlcCer d40:1 | -0.002 | -0.158 |
| LPC 16:0 B | -0.006 | -0.167 |
| PC p-36:4/PC o-36:5 | -0.006 | -0.168 |
| CL 70:7 CL 34:3-36:4 | -0.008 | -0.173 |
| LPC 18:0 | -0.009 | -0.175 |
| FA 20:5 (eicosapentaenoic acid) | -0.011 | -0.180 |
| PC 38:5 B (2) | -0.012 | -0.182 |
| PE 38:4 PE 18:1-20:3 | -0.012 | -0.182 |
| BMP 34:1 BMP 16:0-18:1 | -0.013 | -0.184 |
| LPC 16:0 | -0.016 | -0.192 |
| CE 20:4 | -0.016 | -0.192 |
| LPC 16:1 (2) | -0.017 | -0.195 |
| LPC 16:1 | -0.019 | -0.199 |
| PC 36:2 | -0.025 | -0.213 |
| PC p-36:3 or PC o-36:4 | -0.027 | -0.218 |
| GlcCer d42:1 | -0.028 | -0.221 |
| SM d36:0 | -0.029 | -0.223 |
| PC 35:1 (2) | -0.029 | -0.224 |
| PE 34:3 PE 16:1-18:2 | -0.030 | -0.225 |
| PE 36:5 A | -0.030 | -0.226 |
| PC p-38:3 or PC o-38:4 | -0.031 | -0.228 |
| SM d38:0 | -0.032 | -0.230 |
| LPC 18:1 | -0.032 | -0.232 |
| PC 38:6 | -0.034 | -0.235 |
| PE 38:4e PE 18:0e/20:4 | -0.034 | -0.236 |
| PC 34:3 A | -0.037 | -0.242 |
| PC 36:4 C | -0.037 | -0.243 |
| PE 40:8 A | -0.039 | -0.248 |
| PC 38:5 A | -0.039 | -0.249 |
| SM d34:1 (2) | -0.039 | -0.249 |
| PC 36:3 B (2) | -0.039 | -0.249 |
| PC 36:3 A | -0.040 | -0.252 |
| PC p-36:3 or PC o-36:4 2 | -0.041 | -0.252 |
| LPE 18:2e | -0.041 | -0.254 |
| LPC 20:3 (2) | -0.045 | -0.262 |
| PE p-36:5 or PE o-36:6 | -0.045 | -0.263 |
| LPE 19:0 | -0.046 | -0.266 |
| PC 36:2 (2) | -0.047 | -0.268 |
| PE 36:5 PE 16:1-20:4 | -0.048 | -0.271 |
| AC 18:0 | -0.050 | -0.276 |
| PC 38:5 B | -0.054 | -0.286 |
| LPC 17:1 | -0.055 | -0.289 |
| Cer-NS d43:1 Cer-NS d18:1/25:0 | -0.056 | -0.291 |
| FA 20:2 | -0.057 | -0.294 |
| SM d40:0 | -0.058 | -0.295 |
| PE 36:4 | -0.063 | -0.306 |
| BMP 44:12 BMP 22:6-22:6 | -0.063 | -0.307 |
| LPC 18:1 (2) | -0.064 | -0.311 |
| PC p-38:4 or PC o-38:5 A | -0.068 | -0.320 |
| PC 40:6 B | -0.069 | -0.322 |
| PC 34:2 | -0.069 | -0.322 |
| PC o-34:0 | -0.070 | -0.325 |
| PE p-38:6 or PE o-38:7 | -0.070 | -0.325 |
| PC 40:6 B (2) | -0.071 | -0.328 |
| PC 33:1 (2) | -0.073 | -0.333 |
| PC p-38:3/PC o-38:4 B | -0.081 | -0.351 |
| PE 38:6 | -0.082 | -0.355 |
| LPE 18:1 (2) | -0.083 | -0.357 |
| Cer-NS d43:1 Cer-NS d18:1/25:0 (2) | -0.096 | -0.388 |
| FA 15:1 B | -0.101 | -0.401 |
| SM d43:1 A | -0.101 | -0.402 |
| PC 36:3 B | -0.111 | -0.426 |
| CL 72:7 CL 18:1-18:2-18:2-18:2 | -0.118 | -0.443 |
| PE 38:2 | -0.121 | -0.451 |
| PE p-38:4 or PE o-38:5 | -0.124 | -0.459 |
| CL 74:9 | -0.125 | -0.461 |
| PE p-36:4 or PE o-36:5 | -0.126 | -0.462 |
| PC 38:6 C | -0.126 | -0.463 |
| CL 70:5 | -0.126 | -0.464 |
| PC 31:1 | -0.128 | -0.467 |
| LPC 20:5 (2) | -0.132 | -0.478 |
| PC 33:0 | -0.135 | -0.484 |
| PE p-40:6 or PE o-40:7 | -0.135 | -0.485 |
| LPE 16:1 | -0.149 | -0.519 |
| PC 36:1 (2) | -0.152 | -0.527 |
| PC 38:3 B | -0.171 | -0.575 |
| PI 38:4 | -0.178 | -0.590 |
| PC 32:1 (2) | -0.179 | -0.593 |
| PC 38:4 A | -0.184 | -0.607 |
| FA 18:1 (oleic acid) | -0.185 | -0.609 |
| SM d34:0 | -0.186 | -0.610 |
| FA 17:2 | -0.192 | -0.626 |
| ACar 18:2 | -0.192 | -0.627 |
| PC 38:3 | -0.194 | -0.631 |
| PC 38:4 C | -0.196 | -0.636 |
| SM d34:0 (2) | -0.197 | -0.638 |
| PC 33:1 | -0.198 | -0.641 |
| PE p-38:3 or PE o-38:4 | -0.203 | -0.653 |
| CL 68:2 | -0.205 | -0.658 |
| PC 34:1 (2) | -0.218 | -0.691 |
| PE 36:5 B | -0.238 | -0.739 |
| FA 18:4 | -0.245 | -0.756 |
| PC 36:1 | -0.259 | -0.791 |
| SM d43:1 | -0.262 | -0.798 |
| PC p-38:6/PC o-38:7 | -0.270 | -0.818 |
| ACar 20:4 | -0.274 | -0.827 |
| CE 20:5 | -0.279 | -0.839 |
| PC 34:1 | -0.281 | -0.845 |
| CE 18:1 | -0.282 | -0.846 |
| FA 18:3 (linolenic acid) | -0.284 | -0.853 |
| FA 17:1 | -0.284 | -0.853 |
| FA 14:1 (physeteric acid) | -0.285 | -0.854 |
| PE 39:4 | -0.293 | -0.874 |
| FA 15:1 A | -0.295 | -0.879 |
| PI 40:6 | -0.297 | -0.883 |
| CL 72:6 CL 36:3-36:3 | -0.299 | -0.890 |
| PC 32:1 | -0.300 | -0.893 |
| SM d34:2 (2) | -0.306 | -0.907 |
| CL 70:4 CL 34:1-36:3 | -0.326 | -0.957 |
| CE 18:2 | -0.332 | -0.972 |
| SM d34:2 | -0.339 | -0.989 |
| LNAPS 38:5 LNAPS 18:0/n-20:5 | -0.342 | -0.995 |
| LNAPS 38:6 | -0.344 | -1.001 |
| FA 14:0 (myristic acid) | -0.351 | -1.018 |
| PE p-38:5 or PE o-38:6 | -0.384 | -1.099 |
| FA 16:1 (palmitoleic acid) | -0.385 | -1.101 |
| LNAPS 36:4 LNAPS 16:0/n-20:4 | -0.401 | -1.140 |
| CE 18:3 | -0.415 | -1.176 |
| PE p-36:1 or PE o-36:2 | -0.431 | -1.216 |
| CE 22:6 | -0.436 | -1.227 |
| LNAPS 40:5 LNAPS 18:0/n-22:5 | -0.459 | -1.284 |
| LNAPS 36:2 LNAPS 17:2/n-19:0 | -0.468 | -1.305 |
| LNAPS 38:4 LNAPS 17:2/n-21:2 | -0.505 | -1.396 |
| PE p-40:7 or PE o-40:8 | -0.507 | -1.403 |
| LNAPS 40:6 LNAPS 18:0/n-22:6 | -0.511 | -1.412 |
| DAG 34:3e DAG 19:2e/15:1 | -0.531 | -1.461 |
| DAG 36:4e DAG 19:2e/17:2 | -0.563 | -1.539 |
| PE p-36:2 or PE o-36:3 | -0.644 | -1.739 |
| TAG 60:12 TAG 20:4-20:4-20:4 | -0.650 | -1.755 |
| TAG 48:0 | -0.654 | -1.765 |
| PE p-34:2 or PE o-34:3 | -0.665 | -1.793 |
| DG 38:6 | -0.695 | -1.865 |
| DAG 32:0 DAG 16:0-16:0 | -0.740 | -1.977 |
| DAG 40:6 DAG 18:2-22:4 | -0.755 | -2.014 |
| TAG 48:4 B | -0.768 | -2.047 |
| DG 36:5 | -0.772 | -2.057 |
| TAG 56:8 TAG 16:0-18:2-22:6 | -0.776 | -2.066 |
| DAG 38:4 DAG 18:2-20:2 | -0.800 | -2.125 |
| TAG 55:3 | -0.805 | -2.138 |
| DAG 32:1 DAG 14:0-18:1 | -0.812 | -2.154 |
| PE p-34:1 or PE o-34:2 | -0.816 | -2.164 |
| TAG 53:4 | -0.819 | -2.172 |
| TAG 50:2 | -0.820 | -2.174 |
| TAG 52:2 TAG 16:0-18:1-18:1 | -0.821 | -2.176 |
| TAG 60:7 | -0.827 | -2.191 |
| DAG 38:3 DAG 20:1-18:2 | -0.830 | -2.200 |
| DG 38:5 | -0.833 | -2.206 |
| DG 36:6 | -0.834 | -2.209 |
| DG 36:1 | -0.836 | -2.213 |
| DG 36:4 A | -0.839 | -2.222 |
| DG 36:4 B | -0.840 | -2.224 |
| TAG 52:1 | -0.841 | -2.226 |
| TAG 56:6 TAG 18:1-18:1-20:4 | -0.844 | -2.233 |
| TAG 48:3 | -0.846 | -2.239 |
| DG 34:2 | -0.847 | -2.240 |
| DG 34:1 | -0.850 | -2.248 |
| DAG 35:2 DAG 17:1-18:1 | -0.850 | -2.249 |
| TAG 53:2 | -0.851 | -2.251 |
| DG 36:2 | -0.858 | -2.268 |
| TAG 54:3 TAG 16:0-18:0-20:3 | -0.860 | -2.272 |
| TAG 54:2 | -0.860 | -2.273 |
| TAG 52:4 TAG 16:0-18:2-18:2 | -0.862 | -2.277 |
| DAG 32:2 DAG 16:1-16:1 | -0.864 | -2.282 |
| TAG 56:9 TAG 18:2-18:2-20:5 | -0.868 | -2.291 |
| DG 36:3 | -0.871 | -2.301 |
| TAG 56:3 TAG 18:1-18:1-20:1 | -0.879 | -2.319 |
| TAG 50:5 | -0.879 | -2.320 |
| TAG 53:3 TAG 17:1-17:1-19:1 | -0.880 | -2.323 |
| TAG 52:7 TAG 16:1-16:1-20:5 | -0.883 | -2.329 |
| TAG 51:3 | -0.885 | -2.333 |
| TAG 54:6 | -0.885 | -2.335 |
| TAG 54:3 TAG 16:0-18:1-20:2 | -0.888 | -2.341 |
| TAG 52:5 TAG 16:0-16:0-20:5 | -0.892 | -2.352 |
| TAG 50:4 TAG 16:1-16:1-18:2 | -0.894 | -2.357 |
| DG 34:3 | -0.895 | -2.359 |

**Table S5**. PC3

| **Lipids** | **PC3 Loadings** | **PC3_Zscore** |
| --- | --- | --- |
| FA 20:3 (eicosatrienoic acid) | 0.935 | 2.231 |
| FA 20:2 | 0.922 | 2.192 |
| FA 22:3 | 0.920 | 2.183 |
| FA 18:1 (oleic acid) | 0.906 | 2.140 |
| FA 18:2 (linoleic acid) | 0.897 | 2.113 |
| FA 15:0 (pentadecylic acid) | 0.891 | 2.093 |
| FA 16:0 (palmitic acid) | 0.888 | 2.085 |
| LPE 19:0 | 0.882 | 2.065 |
| FA 18:1 | 0.876 | 2.046 |
| FA 15:1 B | 0.873 | 2.035 |
| LPE 16:0 | 0.863 | 2.006 |
| FA 22:5 | 0.857 | 1.986 |
| FA 17:2 | 0.854 | 1.979 |
| FA 22:2 | 0.848 | 1.959 |
| FA 17:1 | 0.848 | 1.959 |
| LPE 18:0 (2) | 0.846 | 1.950 |
| PC 18:0e | 0.843 | 1.944 |
| LPE 16:0 B | 0.843 | 1.943 |
| LPE 16:0 A | 0.842 | 1.940 |
| LPC 18:0 B | 0.835 | 1.918 |
| FA 19:1 | 0.833 | 1.912 |
| FA 14:0 (myristic acid) | 0.831 | 1.905 |
| FA 21:1 | 0.829 | 1.897 |
| LPC 18:0 | 0.819 | 1.868 |
| FA 20:1 | 0.810 | 1.838 |
| FA 24:6 | 0.810 | 1.838 |
| FA 20:5 (eicosapentaenoic acid) | 0.808 | 1.831 |
| LPE 17:0 | 0.806 | 1.827 |
| LPE 17:0 (2) | 0.804 | 1.819 |
| FA 18:3 (linolenic acid) | 0.786 | 1.763 |
| FA 22:6 (docosahexaenoic acid) | 0.782 | 1.751 |
| FA 20:3 (homo-gamma-linolenic acid) | 0.772 | 1.720 |
| FAHFA 32:0 FAHFA 16:0/16:0 | 0.769 | 1.712 |
| CE 20:5 | 0.766 | 1.702 |
| FA 19:0 | 0.760 | 1.684 |
| FA 20:2 (eicosadienoic acid) | 0.759 | 1.680 |
| FA 16:1 (palmitoleic acid) | 0.751 | 1.654 |
| Cer-NS d43:1 Cer-NS d18:1/25:0 (2) | 0.748 | 1.646 |
| CE 22:6 | 0.741 | 1.623 |
| DAG 36:4e DAG 19:2e/17:2 | 0.735 | 1.605 |
| CE 18:2 | 0.734 | 1.600 |
| CE 18:1 | 0.733 | 1.597 |
| FA 22:1 | 0.727 | 1.580 |
| PC o-34:0 | 0.722 | 1.562 |
| SM d43:1 A | 0.720 | 1.557 |
| LPE 18:0 | 0.708 | 1.520 |
| FA 16:2 | 0.707 | 1.517 |
| LPC 20:0 | 0.706 | 1.513 |
| CE 18:3 | 0.705 | 1.510 |
| PE p-40:7 or PE o-40:8 | 0.695 | 1.477 |
| FA 15:1 A | 0.693 | 1.472 |
| Cer-NS d43:1 Cer-NS d18:1/25:0 | 0.691 | 1.467 |
| FA 14:1 (physeteric acid) | 0.687 | 1.454 |
| GlcCer d40:1 (2) | 0.687 | 1.453 |
| DAG 34:3e DAG 19:2e/15:1 | 0.682 | 1.438 |
| FA 20:0 (arachidic acid) | 0.678 | 1.426 |
| FA 34:1 | 0.674 | 1.413 |
| FA 20:4 (arachidonic acid) | 0.673 | 1.410 |
| SM d43:1 | 0.671 | 1.401 |
| FA 17:0 (margaric acid) | 0.666 | 1.388 |
| LPC 19:0-SN1 | 0.664 | 1.381 |
| CE 20:4 | 0.660 | 1.368 |
| FA 18:4 | 0.659 | 1.364 |
| FA 20:5 | 0.636 | 1.292 |
| LPE 20:0 | 0.632 | 1.279 |
| FA 32:1 | 0.630 | 1.275 |
| PC 34:0 (2) | 0.615 | 1.227 |
| FA 16:3 | 0.613 | 1.220 |
| Cer-NS d40:2 Cer-NS d18:1/22:1 | 0.603 | 1.191 |
| GlcCer d40:1 | 0.601 | 1.183 |
| SM d34:0 (2) | 0.599 | 1.176 |
| Cer-NS d42:1 Cer-NS d18:1/24:0 A | 0.597 | 1.170 |
| LPC p-18:0 or LPC o-18:1 | 0.595 | 1.166 |
| FA 20:0 | 0.584 | 1.131 |
| FA 30:1 | 0.583 | 1.127 |
| LPE 19:0 (2) | 0.583 | 1.127 |
| TAG 60:12 TAG 20:4-20:4-20:4 | 0.578 | 1.110 |
| SM d34:0 | 0.576 | 1.103 |
| LPC o-16:0 | 0.566 | 1.074 |
| Ceramide d34:0 | 0.566 | 1.072 |
| LPC 16:0 A | 0.564 | 1.066 |
| SM d34:1 (2) | 0.562 | 1.060 |
| LPC 16:0 | 0.557 | 1.046 |
| LPE 18:1e | 0.553 | 1.032 |
| LPE 18:1e (2) | 0.548 | 1.018 |
| LPE 18:2 B | 0.544 | 1.006 |
| LPE 18:2 | 0.543 | 1.002 |
| PE 40:8 PE 20:4-20:4 | 0.538 | 0.985 |
| PG 40:8 PG 18:2-22:6 | 0.534 | 0.973 |
| SM d40:0 | 0.526 | 0.948 |
| PE p-36:2 or PE o-36:3 | 0.524 | 0.943 |
| PC 34:0 | 0.522 | 0.935 |
| LPE 20:1 | 0.521 | 0.932 |
| Cer-NS d41:1 Cer-NS d18:1/23:0 B | 0.518 | 0.924 |
| Ceramide d34:1 | 0.518 | 0.923 |
| GlcCer d42:1 | 0.515 | 0.915 |
| CL 68:2 | 0.514 | 0.912 |
| Cer-NS d34:1 Cer-NS d18:1/16:0 | 0.514 | 0.909 |
| LPC 16:0 B | 0.512 | 0.904 |
| SM d34:1 | 0.511 | 0.900 |
| FA 24:5 | 0.510 | 0.898 |
| Cer d34:1 | 0.508 | 0.890 |
| LPE 16:1e | 0.504 | 0.880 |
| FAHFA 18:0 FAHFA 7:0/11:0 | 0.500 | 0.866 |
| Ceramide d40:0 | 0.497 | 0.856 |
| LPE 20:4 B | 0.495 | 0.850 |
| FA 21:0 | 0.492 | 0.842 |
| CL 70:5 | 0.491 | 0.839 |
| CL 70:4 CL 34:1-36:3 | 0.490 | 0.836 |
| Ceramide d32:1 | 0.487 | 0.825 |
| LPE 18:1 (2) | 0.483 | 0.814 |
| PC 38:1 | 0.467 | 0.763 |
| LPC 18:0 A | 0.465 | 0.756 |
| PC p-38:5/PC o-38:6 A | 0.461 | 0.745 |
| TAG 56:8 TAG 16:0-18:2-22:6 | 0.460 | 0.742 |
| PE p-34:2 or PE o-34:3 | 0.458 | 0.733 |
| FA 24:4 | 0.455 | 0.725 |
| Ceramide d44:1 | 0.455 | 0.724 |
| PC 32:0 (2) | 0.453 | 0.719 |
| PC p-32:0 or PC o-32:1 2 | 0.453 | 0.717 |
| TAG 60:7 | 0.450 | 0.710 |
| SM d36:0 | 0.450 | 0.708 |
| GlcCer d41:1 | 0.431 | 0.650 |
| Ceramide d34:2 | 0.428 | 0.641 |
| PC p-32:0 or PC o-32:1 | 0.427 | 0.637 |
| TAG 53:4 | 0.425 | 0.630 |
| DAG 32:0 DAG 16:0-16:0 | 0.424 | 0.628 |
| LPC 20:1 | 0.419 | 0.613 |
| SM d42:0 | 0.419 | 0.612 |
| SM d38:0 | 0.412 | 0.590 |
| TAG 48:0 | 0.411 | 0.587 |
| LPC 22:4 | 0.409 | 0.582 |
| TAG 56:6 TAG 18:1-18:1-20:4 | 0.408 | 0.579 |
| Ceramide d33:1 | 0.406 | 0.572 |
| LPC 20:3 | 0.405 | 0.570 |
| LPE 18:2e | 0.405 | 0.567 |
| PC 33:0 | 0.403 | 0.562 |
| TAG 54:3 TAG 16:0-18:0-20:3 | 0.401 | 0.556 |
| TAG 52:2 TAG 16:0-18:1-18:1 | 0.397 | 0.544 |
| PC o-32:0 (2) | 0.396 | 0.540 |
| LPE 22:6 (2) | 0.392 | 0.526 |
| FA 24:1 | 0.383 | 0.498 |
| PC p-34:0 or PC o-34:1 | 0.382 | 0.495 |
| SM d32:1 | 0.382 | 0.495 |
| TAG 52:4 TAG 16:0-18:2-18:2 | 0.379 | 0.488 |
| PE p-40:6 or PE o-40:7 | 0.379 | 0.488 |
| PE p-36:1 or PE o-36:2 | 0.377 | 0.481 |
| LPE 20:2 (2) | 0.371 | 0.461 |
| PE 39:4 | 0.370 | 0.459 |
| PC p-38:5 or PC o-38:6 | 0.366 | 0.446 |
| PC o-32:0 | 0.362 | 0.434 |
| LPE 20:4 | 0.362 | 0.433 |
| AC 18:0 | 0.360 | 0.428 |
| LPE 16:1 | 0.357 | 0.418 |
| CL 72:6 CL 36:3-36:3 | 0.357 | 0.418 |
| TAG 56:9 TAG 18:2-18:2-20:5 | 0.356 | 0.416 |
| DG 36:4 B | 0.355 | 0.410 |
| TAG 53:2 | 0.354 | 0.409 |
| LPC 18:1 (2) | 0.353 | 0.405 |
| Cer-NS d42:2 Cer-NS d18:1/24:1 | 0.353 | 0.404 |
| LPC 14:0 (2) | 0.352 | 0.401 |
| LPE 22:6 | 0.349 | 0.393 |
| PC 36:1 (2) | 0.348 | 0.388 |
| TAG 52:7 TAG 16:1-16:1-20:5 | 0.345 | 0.381 |
| PG 36:4 PG 18:2-18:2 | 0.344 | 0.377 |
| LPC p-16:0 or LPC o-16:1 | 0.343 | 0.375 |
| PE 38:7 | 0.342 | 0.372 |
| TAG 50:5 | 0.339 | 0.362 |
| PC 36:1 | 0.334 | 0.345 |
| DG 36:5 | 0.334 | 0.344 |
| TAG 51:3 | 0.332 | 0.340 |
| CholesterolSulfate | 0.332 | 0.340 |
| SM d40:2 A | 0.332 | 0.339 |
| TAG 52:1 | 0.331 | 0.336 |
| LPC 18:1 | 0.328 | 0.328 |
| FA 12:0 (lauric acid) | 0.324 | 0.313 |
| TAG 54:3 TAG 16:0-18:1-20:2 | 0.324 | 0.313 |
| LPE 20:2 | 0.322 | 0.308 |
| TAG 56:3 TAG 18:1-18:1-20:1 | 0.322 | 0.308 |
| TAG 54:2 | 0.320 | 0.303 |
| TAG 53:3 TAG 17:1-17:1-19:1 | 0.319 | 0.297 |
| LPC 20:3 (2) | 0.318 | 0.295 |
| DG 36:2 | 0.317 | 0.291 |
| SM d40:2 A (2) | 0.316 | 0.290 |
| TAG 54:6 | 0.315 | 0.286 |
| FA 23:0 | 0.311 | 0.274 |
| FA 26:1 | 0.308 | 0.264 |
| LPE 20:3 (2) | 0.305 | 0.255 |
| LPC 22:5 (2) | 0.303 | 0.249 |
| DG 36:3 | 0.301 | 0.242 |
| TAG 52:5 TAG 16:0-16:0-20:5 | 0.296 | 0.227 |
| PC 40:8 | 0.293 | 0.218 |
| LPE 20:4 A | 0.292 | 0.215 |
| PE 35:0 PE 17:0-18:0 | 0.286 | 0.195 |
| LPE 20:3 | 0.284 | 0.188 |
| TAG 48:3 | 0.279 | 0.173 |
| PC 32:0 | 0.276 | 0.164 |
| SM d32:1 (2) | 0.273 | 0.155 |
| DAG 38:4 DAG 18:2-20:2 | 0.273 | 0.154 |
| PC 34:1 (2) | 0.273 | 0.153 |
| TAG 50:4 TAG 16:1-16:1-18:2 | 0.271 | 0.148 |
| LPC 22:5 | 0.270 | 0.146 |
| DAG 40:6 DAG 18:2-22:4 | 0.269 | 0.142 |
| LPC 18:2 B | 0.268 | 0.137 |
| PE p-34:1 or PE o-34:2 | 0.267 | 0.135 |
| BMP 34:1 BMP 16:0-18:1 | 0.266 | 0.133 |
| Ceramide d42:2 A | 0.263 | 0.124 |
| DAG 35:2 DAG 17:1-18:1 | 0.263 | 0.122 |
| TAG 50:2 | 0.258 | 0.107 |
| GlcCer d42:2 (2) | 0.257 | 0.104 |
| GlcCer d42:2 | 0.254 | 0.094 |
| SM d40:1 (2) | 0.252 | 0.089 |
| SMd30:1 | 0.252 | 0.089 |
| TAG 48:4 B | 0.252 | 0.087 |
| PC p-38:3/PC o-38:4 B | 0.250 | 0.083 |
| PC 40:8 (2) | 0.248 | 0.075 |
| LPC 17:1 | 0.247 | 0.073 |
| PC 40:6 B (2) | 0.246 | 0.070 |
| PE 40:9 | 0.245 | 0.065 |
| PC p-40:3/PC o-40:4 | 0.244 | 0.063 |
| Cer d42:2 A | 0.242 | 0.058 |
| TAG 55:3 | 0.240 | 0.051 |
| PI 40:8 PI 20:4-20:4 | 0.234 | 0.032 |
| LPC 16:1 (2) | 0.234 | 0.030 |
| DAG 36:0 DAG 18:0-18:0 | 0.232 | 0.027 |
| DG 36:6 | 0.230 | 0.020 |
| SM d44:1 | 0.224 | 0.000 |
| PE p-38:6 or PE o-38:7 | 0.220 | -0.013 |
| PG 36:3 PG 18:1-18:2 | 0.216 | -0.024 |
| DAG 38:3 DAG 20:1-18:2 | 0.216 | -0.024 |
| LPE 18:1 | 0.213 | -0.033 |
| PC p-42:3 or PC o-42:4 | 0.213 | -0.035 |
| LPC 20:2 2 | 0.212 | -0.038 |
| PC 40:7 B | 0.210 | -0.042 |
| DG 36:4 A | 0.207 | -0.053 |
| SM d36:1 (2) | 0.206 | -0.055 |
| LPE 18:2 A | 0.200 | -0.074 |
| DG 34:1 | 0.200 | -0.075 |
| Cer-NS d40:1 Cer-NS d18:1/22:0 | 0.198 | -0.082 |
| SM d40:1 | 0.195 | -0.090 |
| PC 34:1 | 0.192 | -0.099 |
| PI 40:6 | 0.192 | -0.101 |
| FA 24:2 | 0.189 | -0.109 |
| SM d42:1 (2) | 0.188 | -0.112 |
| PC 40:6 B | 0.188 | -0.113 |
| PC p-38:3 or PC o-38:4 | 0.186 | -0.120 |
| LPC 16:1 | 0.181 | -0.135 |
| Cer-NS d36:2 Cer-NS d20:1/16:1 | 0.180 | -0.137 |
| DG 38:5 | 0.174 | -0.155 |
| Cer-NS d38:1 Cer-NS d18:1/20:0 | 0.171 | -0.167 |
| LPC 15:0 | 0.170 | -0.170 |
| LPE 22:5 | 0.167 | -0.177 |
| PC 40:7 A | 0.167 | -0.178 |
| PC 32:1 (2) | 0.163 | -0.191 |
| PC 35:1 (2) | 0.161 | -0.198 |
| PC 32:1 | 0.161 | -0.198 |
| PC 38:3 B | 0.160 | -0.200 |
| PC 38:4 A | 0.158 | -0.205 |
| Cer d42:1 | 0.157 | -0.209 |
| Cer d40:1 | 0.156 | -0.214 |
| DG 34:3 | 0.155 | -0.215 |
| Ceramide d40:1 | 0.153 | -0.222 |
| SM d33:1 (2) | 0.150 | -0.232 |
| DG 36:1 | 0.150 | -0.232 |
| PE 38:4e PE 18:0e/20:4 | 0.146 | -0.245 |
| Cer-NS d43:2 Cer-NS d20:2/23:0 | 0.146 | -0.245 |
| PE 38:7 PE 16:1-22:6 | 0.145 | -0.247 |
| PC 33:1 | 0.144 | -0.249 |
| PE p-38:3 or PE o-38:4 | 0.143 | -0.255 |
| PC 40:7 | 0.142 | -0.255 |
| LPC 14:0-SN1 | 0.140 | -0.262 |
| PC 38:3 | 0.140 | -0.262 |
| PC p-38:4/PC o-38:5 A | 0.140 | -0.263 |
| CL 72:7 CL 18:1-18:2-18:2-18:2 | 0.140 | -0.263 |
| PC 38:4 C | 0.138 | -0.271 |
| SM d42:1 | 0.137 | -0.273 |
| LPC 22:6 | 0.135 | -0.280 |
| Cer-NS d42:1 Cer-NS d18:1/24:0 B | 0.131 | -0.290 |
| PC p-36:3 or PC o-36:4 2 | 0.128 | -0.302 |
| LPC 18:2 | 0.123 | -0.316 |
| DAG 32:1 DAG 14:0-18:1 | 0.123 | -0.317 |
| PE p-40:4 or PE o-40:5 | 0.120 | -0.325 |
| Ceramide d42:1 | 0.118 | -0.332 |
| PC p-36:1/PC o-36:2 A | 0.113 | -0.348 |
| LPC 18:3 | 0.112 | -0.350 |
| Cholesterol | 0.110 | -0.357 |
| PE 36:1 | 0.109 | -0.360 |
| PC 30:0 | 0.108 | -0.363 |
| CL 70:6 CL 34:3-36:3 | 0.107 | -0.366 |
| PE 38:4 B | 0.105 | -0.372 |
| PC 36:2 (2) | 0.105 | -0.372 |
| PE 36:4 | 0.105 | -0.374 |
| PC p-36:3 or PC o-36:4 | 0.104 | -0.375 |
| PG 34:2 PG 16:0-18:2 | 0.102 | -0.381 |
| LPC 22:6 2 | 0.102 | -0.384 |
| Gal-Gal-Cer d18:1/16:0 or Lactosylceramide d18:1/16:0 | 0.101 | -0.384 |
| Cer-NS d44:2 Cer-NS d18:1/26:1 | 0.101 | -0.384 |
| PE 36:5 B | 0.101 | -0.387 |
| PC 33:1 (2) | 0.100 | -0.387 |
| PC 40:5 B | 0.099 | -0.392 |
| CL 74:9 | 0.098 | -0.394 |
| PC 31:1 | 0.097 | -0.398 |
| PC 35:1 | 0.097 | -0.399 |
| PC 38:2 | 0.094 | -0.408 |
| PE 36:5 A | 0.091 | -0.417 |
| DG 38:6 | 0.090 | -0.420 |
| LPC 20:2 | 0.089 | -0.424 |
| PE p-38:5 or PE o-38:6 | 0.089 | -0.424 |
| PC p-38:4 or PC o-38:5 A | 0.088 | -0.425 |
| Cer-NS d41:2 Cer-NS d26:2/15:0 | 0.088 | -0.428 |
| Ceramide d36:1 | 0.082 | -0.446 |
| LPC 18:2 A | 0.081 | -0.448 |
| PC 38:2 (2) | 0.081 | -0.449 |
| PC 36:3 A | 0.079 | -0.453 |
| PC 40:4 | 0.079 | -0.455 |
| PE 36:5 PE 16:1-20:4 | 0.078 | -0.458 |
| PE 40:7 B | 0.076 | -0.463 |
| LPC 14:0 | 0.071 | -0.479 |
| SM d42:2 | 0.069 | -0.486 |
| ACar 20:4 | 0.064 | -0.503 |
| PE 34:3 PE 16:1-18:2 | 0.063 | -0.506 |
| PE 38:6 | 0.062 | -0.507 |
| ACar 18:2 | 0.058 | -0.519 |
| SM d44:2 | 0.058 | -0.519 |
| PC 38:6 C | 0.053 | -0.535 |
| PC 40:4 (2) | 0.053 | -0.535 |
| PC 36:3 A (2) | 0.052 | -0.538 |
| PC 35:2 A | 0.046 | -0.557 |
| PC 39:6 | 0.045 | -0.563 |
| PC p-34:1/PC o-34:2 | 0.042 | -0.570 |
| Ceramide d43:1 | 0.041 | -0.573 |
| SM d33:1 | 0.041 | -0.574 |
| PI 40:7 | 0.039 | -0.580 |
| PC 34:3 C | 0.039 | -0.581 |
| LPC 20:4 | 0.038 | -0.584 |
| SM d36:1 | 0.037 | -0.587 |
| PE 40:8 B | 0.036 | -0.588 |
| SM d42:2 A | 0.036 | -0.589 |
| PE p-38:4 or PE o-38:5 | 0.036 | -0.590 |
| Cer d41:1 | 0.033 | -0.597 |
| PE 38:4 A | 0.033 | -0.598 |
| CL 74:8 CL 18:1-18:2-18:2-20:3 | 0.026 | -0.622 |
| PE 38:3 PE 18:0-20:3 | 0.023 | -0.632 |
| PC 36:3 B | 0.022 | -0.634 |
| PC 38:6 A | 0.022 | -0.634 |
| PC p-36:5/PC o-36:6 | 0.020 | -0.640 |
| Cer-NS d41:1 Cer-NS d18:1/23:0 A | 0.017 | -0.649 |
| PE 34:2 (2) | 0.017 | -0.650 |
| Ceramide d41:1 | 0.016 | -0.652 |
| PC 38:5 A | 0.016 | -0.652 |
| PC 38:5 A (2) | 0.015 | -0.654 |
| Cer-NS d42:3 Cer-NS d18:2/24:1 | 0.012 | -0.664 |
| DG 34:2 | 0.010 | -0.670 |
| PC 38:5 B (2) | 0.008 | -0.677 |
| PE 38:5 PE 18:1-20:4 | 0.006 | -0.683 |
| PC 37:4 (2) | 0.006 | -0.684 |
| Cer d36:1 | 0.004 | -0.688 |
| PC 38:3 A | 0.004 | -0.690 |
| PC 36:2 | 0.002 | -0.697 |
| PE 38:5 A | 0.000 | -0.702 |
| BMP 44:12 BMP 22:6-22:6 | -0.001 | -0.705 |
| PC 36:3 B (2) | -0.002 | -0.708 |
| SM d41:2 A (2) | -0.003 | -0.711 |
| SM d38:1 (2) | -0.003 | -0.712 |
| PC 40:5 A (2) | -0.004 | -0.714 |
| SM d34:2 | -0.006 | -0.721 |
| SM d34:2 (2) | -0.007 | -0.724 |
| Cer-NS d38:2 Cer-NS d18:2/20:0 | -0.009 | -0.731 |
| PE 38:2 | -0.010 | -0.733 |
| PE 40:7 A | -0.012 | -0.740 |
| PE 38:4 PE 18:1-20:3 | -0.012 | -0.740 |
| PC p-34:2/PC o-34:3 | -0.013 | -0.743 |
| PE 40:4 PE 18:0-22:4 | -0.014 | -0.748 |
| Ceramide d40:2 | -0.014 | -0.748 |
| Cer-NS d42:3 Cer-NS d18:1/24:2 | -0.016 | -0.753 |
| PC 34:3 A | -0.019 | -0.762 |
| PC 34:2 (2) | -0.020 | -0.764 |
| PE 40:8 A | -0.022 | -0.770 |
| PE 40:7 PE 18:1-22:6 | -0.022 | -0.772 |
| PC 38:5 B | -0.023 | -0.774 |
| PE 36:3 B | -0.023 | -0.775 |
| PI 36:3 PI 16:0-20:3 | -0.024 | -0.779 |
| PC 40:5 A | -0.025 | -0.782 |
| SM d36:2 (2) | -0.028 | -0.791 |
| SM d43:1 B | -0.029 | -0.794 |
| Cer d38:1 | -0.034 | -0.809 |
| PC 36:4 A (2) | -0.035 | -0.813 |
| PE 34:1 | -0.038 | -0.821 |
| SM d39:1 (2) | -0.040 | -0.828 |
| SM d41:1 (2) | -0.050 | -0.858 |
| PE 34:3 PE 16:0-18:3 | -0.052 | -0.864 |
| LPC 20:5 (2) | -0.053 | -0.867 |
| CL 70:7 CL 34:3-36:4 | -0.054 | -0.871 |
| PE 40:6 A | -0.056 | -0.877 |
| PC 35:3 | -0.056 | -0.877 |
| PE 34:2 | -0.057 | -0.882 |
| PC 40:6 A (2) | -0.057 | -0.882 |
| Ceramide d39:1 | -0.060 | -0.890 |
| Ceramide d38:1 | -0.063 | -0.901 |
| SM d41:2 A | -0.063 | -0.902 |
| Cer-NS d41:2 Cer-NS d18:2/23:0 | -0.064 | -0.903 |
| PC 36:4 A | -0.065 | -0.905 |
| PC 37:5 | -0.065 | -0.905 |
| PC p-36:2/PC o-36:3 | -0.065 | -0.907 |
| PE p-36:4 or PE o-36:5 | -0.069 | -0.918 |
| Cer d42:2 B | -0.069 | -0.919 |
| PC 40:6 A | -0.071 | -0.924 |
| PE 37:6 PE 15:0-22:6 | -0.078 | -0.947 |
| PC 38:7 | -0.080 | -0.952 |
| PE p-36:5 or PE o-36:6 | -0.086 | -0.972 |
| Ceramide d42:2 B | -0.086 | -0.972 |
| PC p-34:2 or PC o-34:3 | -0.096 | -1.003 |
| PI 38:4 | -0.098 | -1.008 |
| PC 38:6 | -0.098 | -1.011 |
| PE 40:6 B | -0.099 | -1.013 |
| PC p-36:4/PC o-36:5 | -0.103 | -1.025 |
| DAG 32:2 DAG 16:1-16:1 | -0.104 | -1.028 |
| PC 34:2 | -0.109 | -1.043 |
| PC p-38:4/PC o-38:5 B | -0.113 | -1.058 |
| PC 36:5 D | -0.115 | -1.064 |
| PC 42:6 | -0.118 | -1.074 |
| PC 37:4 | -0.122 | -1.085 |
| PC 36:3e | -0.124 | -1.090 |
| PE 36:4 PE 18:2-18:2 | -0.126 | -1.096 |
| SM d42:2 B | -0.126 | -1.097 |
| PC 31:0 | -0.127 | -1.101 |
| SM d39:1 | -0.128 | -1.105 |
| SM d38:1 | -0.130 | -1.111 |
| LPC 20:5 | -0.130 | -1.112 |
| PC 36:5 B | -0.134 | -1.122 |
| CL 72:8 CL 18:2-18:2-18:2-18:2 | -0.134 | -1.123 |
| PC 35:2 B | -0.136 | -1.128 |
| PC p-38:6/PC o-38:7 | -0.145 | -1.159 |
| PC 36:4 C (2) | -0.148 | -1.166 |
| PC 38:4 B | -0.157 | -1.194 |
| PE 37:4 | -0.159 | -1.201 |
| PC 34:3 B | -0.161 | -1.206 |
| PC 36:5 C | -0.162 | -1.211 |
| PC 38:6 B | -0.162 | -1.212 |
| PC p-38:4 or PC o-38:5 B | -0.164 | -1.217 |
| PE 37:4 PE 17:0-20:4 | -0.165 | -1.220 |
| PE 36:3 A | -0.166 | -1.222 |
| PC 36:4 B | -0.169 | -1.231 |
| SM d40:2 B (2) | -0.174 | -1.248 |
| PE 36:2 (2) | -0.176 | -1.254 |
| PC 36:4 C | -0.179 | -1.264 |
| PE 40:5 PE 18:0-22:5 | -0.185 | -1.281 |
| PE 36:3 | -0.190 | -1.299 |
| PC 37:2 (2) | -0.191 | -1.301 |
| PC 36:6 | -0.194 | -1.310 |
| SM d41:2B 2 | -0.195 | -1.314 |
| SM d42:3 (2) | -0.199 | -1.327 |
| PC 39:4 | -0.200 | -1.331 |
| PC p-34:1 or PC o-34:2 A | -0.204 | -1.344 |
| PE 33:2 PE 15:0-18:2 | -0.213 | -1.372 |
| PC 35:2 | -0.214 | -1.373 |
| SM d38:2 (2) | -0.218 | -1.387 |
| SM d41:2 B | -0.220 | -1.392 |
| PI 36:4 | -0.222 | -1.399 |
| PE 39:6 PE 17:0-22:6 | -0.239 | -1.451 |
| PC 37:2 | -0.240 | -1.455 |
| PE 38:6 (2) | -0.247 | -1.476 |
| PC 32:2 (2) | -0.251 | -1.490 |
| PE 38:5 B | -0.261 | -1.521 |
| PC 34:3 | -0.261 | -1.521 |
| PC 37:6 | -0.267 | -1.540 |
| PE 36:2 | -0.270 | -1.549 |
| PI 38:5 | -0.274 | -1.561 |
| PC 32:2 | -0.276 | -1.567 |
| PE 35:2 PE 17:0-18:2 | -0.309 | -1.673 |
| SM d42:3 | -0.320 | -1.705 |
| PC 36:5 A (2) | -0.326 | -1.725 |
| PC 34:4 | -0.336 | -1.757 |
| PC 36:5 A | -0.337 | -1.760 |
| PC 34:4 (2) | -0.342 | -1.775 |
| PC 33:2 (2) | -0.348 | -1.795 |
| PC 35:4 (2) | -0.356 | -1.820 |
| SM d40:2 B | -0.360 | -1.833 |
| PC 33:2 | -0.382 | -1.899 |
| PC p-36:2 or PC o-36:3 | -0.399 | -1.953 |
| PC 35:4 | -0.412 | -1.995 |
| LNAPS 38:5 LNAPS 18:0/n-20:5 | -0.431 | -2.055 |
| LNAPS 38:4 LNAPS 17:2/n-21:2 | -0.442 | -2.088 |
| LNAPS 36:2 LNAPS 17:2/n-19:0 | -0.443 | -2.093 |
| LNAPS 40:5 LNAPS 18:0/n-22:5 | -0.446 | -2.101 |
| LNAPS 36:4 LNAPS 16:0/n-20:4 | -0.459 | -2.142 |
| LNAPS 40:6 LNAPS 18:0/n-22:6 | -0.470 | -2.178 |
| LNAPS 38:6 | -0.543 | -2.405 |

**Table S6**. PC4

| **Lipids** | **PC4 Loadings** | **PC4_Zscore** |
| --- | --- | --- |
| PC p-36:3 or PC o-36:4 | 0.775 | 2.591 |
| PE p-38:4 or PE o-38:5 | 0.722 | 2.403 |
| PC p-38:4 or PC o-38:5 A | 0.721 | 2.398 |
| PC p-38:4 or PC o-38:5 B | 0.709 | 2.355 |
| PE p-36:4 or PE o-36:5 | 0.701 | 2.329 |
| PC p-38:4/PC o-38:5 B | 0.701 | 2.328 |
| PC p-36:3 or PC o-36:4 2 | 0.694 | 2.304 |
| PE 40:4 PE 18:0-22:4 | 0.694 | 2.302 |
| PC p-36:4/PC o-36:5 | 0.691 | 2.293 |
| PC 32:0 | 0.689 | 2.286 |
| SM d34:1 | 0.677 | 2.243 |
| PC p-38:3 or PC o-38:4 | 0.673 | 2.229 |
| SM d34:2 | 0.673 | 2.229 |
| PC p-38:4/PC o-38:5 A | 0.647 | 2.136 |
| PC p-38:3/PC o-38:4 B | 0.646 | 2.132 |
| PE p-40:4 or PE o-40:5 | 0.643 | 2.124 |
| PC 32:0 (2) | 0.637 | 2.101 |
| PE 38:4 B | 0.631 | 2.081 |
| SM d34:2 (2) | 0.607 | 1.996 |
| SM d40:2 A | 0.603 | 1.981 |
| PE p-38:5 or PE o-38:6 | 0.602 | 1.977 |
| SM d42:3 | 0.595 | 1.954 |
| SM d40:0 | 0.593 | 1.946 |
| PC o-32:0 | 0.589 | 1.933 |
| SM d34:1 (2) | 0.580 | 1.900 |
| SM d36:2 (2) | 0.569 | 1.861 |
| PC 36:4 C | 0.566 | 1.851 |
| PC o-32:0 (2) | 0.561 | 1.832 |
| SM d33:1 (2) | 0.558 | 1.823 |
| PE p-38:6 or PE o-38:7 | 0.557 | 1.817 |
| SM d34:0 (2) | 0.546 | 1.780 |
| SM d36:1 | 0.537 | 1.748 |
| PC 38:4 A | 0.536 | 1.742 |
| PE 38:4e PE 18:0e/20:4 | 0.524 | 1.701 |
| SM d42:3 (2) | 0.520 | 1.686 |
| PE 38:4 A | 0.517 | 1.678 |
| SM d38:0 | 0.513 | 1.661 |
| SM d33:1 | 0.504 | 1.630 |
| PC 37:4 | 0.502 | 1.622 |
| PC p-32:0 or PC o-32:1 2 | 0.501 | 1.618 |
| PC 38:4 C | 0.497 | 1.606 |
| PC p-40:3/PC o-40:4 | 0.493 | 1.590 |
| PC 36:4 B | 0.487 | 1.570 |
| CL 72:8 CL 18:2-18:2-18:2-18:2 | 0.486 | 1.568 |
| Cholesterol | 0.482 | 1.553 |
| PC 39:4 | 0.481 | 1.550 |
| SM d40:2 A (2) | 0.472 | 1.516 |
| SM d38:1 | 0.469 | 1.507 |
| PE 37:4 PE 17:0-20:4 | 0.456 | 1.461 |
| PC p-34:0 or PC o-34:1 | 0.456 | 1.460 |
| DG 38:6 | 0.437 | 1.395 |
| SM d44:2 | 0.433 | 1.380 |
| PC p-34:1 or PC o-34:2 A | 0.427 | 1.360 |
| PG 36:3 PG 18:1-18:2 | 0.425 | 1.352 |
| SM d38:1 (2) | 0.420 | 1.334 |
| PE 37:4 | 0.412 | 1.307 |
| SM d42:0 | 0.411 | 1.301 |
| CholesterolSulfate | 0.400 | 1.261 |
| Ceramide d34:0 | 0.397 | 1.250 |
| FA 16:3 | 0.394 | 1.241 |
| PE 36:5 B | 0.384 | 1.205 |
| SM d40:2 B | 0.377 | 1.181 |
| SM d36:1 (2) | 0.370 | 1.155 |
| PG 34:2 PG 16:0-18:2 | 0.369 | 1.154 |
| PC 36:4 C (2) | 0.368 | 1.149 |
| PC 34:2 | 0.368 | 1.148 |
| CE 18:1 | 0.366 | 1.142 |
| CE 18:3 | 0.364 | 1.135 |
| SM d39:1 | 0.361 | 1.126 |
| CE 18:2 | 0.358 | 1.114 |
| PE 38:6 | 0.354 | 1.099 |
| SM d39:1 (2) | 0.351 | 1.091 |
| FA 16:2 | 0.351 | 1.089 |
| SM d40:1 | 0.341 | 1.055 |
| SM d42:2 A | 0.340 | 1.051 |
| PE 40:7 B | 0.340 | 1.049 |
| DG 36:5 | 0.334 | 1.029 |
| CL 68:2 | 0.331 | 1.019 |
| PC 31:0 | 0.328 | 1.007 |
| PC p-36:2 or PC o-36:3 | 0.327 | 1.006 |
| PG 36:4 PG 18:2-18:2 | 0.323 | 0.991 |
| DG 36:4 A | 0.321 | 0.982 |
| PE 39:4 | 0.313 | 0.953 |
| PE 36:4 | 0.313 | 0.953 |
| Ceramide d40:0 | 0.309 | 0.941 |
| PE p-40:7 or PE o-40:8 | 0.309 | 0.939 |
| ACar 20:4 | 0.308 | 0.938 |
| PE 36:2 (2) | 0.306 | 0.931 |
| GlcCer d42:2 | 0.303 | 0.919 |
| SM d42:2 | 0.303 | 0.919 |
| FA 24:2 | 0.298 | 0.902 |
| GlcCer d42:2 (2) | 0.294 | 0.887 |
| SM d41:2 A | 0.294 | 0.886 |
| PE 38:7 | 0.293 | 0.882 |
| LPC 20:4 | 0.291 | 0.878 |
| LPE 20:4 A | 0.290 | 0.873 |
| CL 74:8 CL 18:1-18:2-18:2-20:3 | 0.290 | 0.873 |
| SM d36:0 | 0.290 | 0.871 |
| Ceramide d34:2 | 0.289 | 0.868 |
| CL 72:7 CL 18:1-18:2-18:2-18:2 | 0.289 | 0.868 |
| PC p-38:5 or PC o-38:6 | 0.288 | 0.867 |
| SM d40:2 B (2) | 0.287 | 0.861 |
| SM d38:2 (2) | 0.286 | 0.859 |
| PC p-38:6/PC o-38:7 | 0.285 | 0.854 |
| PE p-34:1 or PE o-34:2 | 0.279 | 0.835 |
| PC p-34:1/PC o-34:2 | 0.278 | 0.830 |
| Cer-NS d36:2 Cer-NS d20:1/16:1 | 0.277 | 0.829 |
| PC 30:0 | 0.277 | 0.828 |
| CE 22:6 | 0.276 | 0.825 |
| PC 35:4 | 0.275 | 0.820 |
| FA 12:0 (lauric acid) | 0.273 | 0.811 |
| Cer-NS d44:2 Cer-NS d18:1/26:1 | 0.265 | 0.784 |
| Cer-NS d34:1 Cer-NS d18:1/16:0 | 0.263 | 0.776 |
| PC 36:3e | 0.262 | 0.775 |
| PE p-36:1 or PE o-36:2 | 0.262 | 0.775 |
| PE 38:5 B | 0.255 | 0.749 |
| FA 24:4 | 0.247 | 0.721 |
| SM d41:2 A (2) | 0.245 | 0.715 |
| FA 18:4 | 0.244 | 0.711 |
| PE 35:2 PE 17:0-18:2 | 0.242 | 0.703 |
| Ceramide d34:1 | 0.242 | 0.703 |
| PC p-38:5/PC o-38:6 A | 0.241 | 0.699 |
| PC 40:4 | 0.240 | 0.697 |
| ACar 18:2 | 0.239 | 0.692 |
| PC 34:0 | 0.238 | 0.688 |
| DAG 40:6 DAG 18:2-22:4 | 0.238 | 0.687 |
| LNAPS 38:4 LNAPS 17:2/n-21:2 | 0.237 | 0.685 |
| PE 36:2 | 0.237 | 0.684 |
| TAG 60:12 TAG 20:4-20:4-20:4 | 0.230 | 0.659 |
| DG 38:5 | 0.224 | 0.638 |
| PE 34:2 | 0.223 | 0.637 |
| Cer-NS d38:2 Cer-NS d18:2/20:0 | 0.221 | 0.630 |
| CE 20:4 | 0.216 | 0.611 |
| PE 36:1 | 0.214 | 0.604 |
| SM d40:1 (2) | 0.207 | 0.579 |
| SM d34:0 | 0.200 | 0.556 |
| FA 20:5 | 0.198 | 0.546 |
| PE 40:9 | 0.193 | 0.529 |
| SM d42:1 | 0.191 | 0.523 |
| DG 34:2 | 0.190 | 0.520 |
| GlcCer d40:1 | 0.186 | 0.504 |
| Ceramide d36:1 | 0.185 | 0.500 |
| LNAPS 40:5 LNAPS 18:0/n-22:5 | 0.184 | 0.497 |
| LPE 20:4 | 0.182 | 0.492 |
| DAG 36:0 DAG 18:0-18:0 | 0.182 | 0.491 |
| LPE 20:4 B | 0.181 | 0.489 |
| Cer d34:1 | 0.180 | 0.483 |
| PC p-32:0 or PC o-32:1 | 0.174 | 0.462 |
| Ceramide d44:1 | 0.173 | 0.460 |
| LNAPS 36:2 LNAPS 17:2/n-19:0 | 0.169 | 0.444 |
| TAG 54:6 | 0.166 | 0.434 |
| PE p-40:6 or PE o-40:7 | 0.164 | 0.428 |
| SMd30:1 | 0.164 | 0.426 |
| Ceramide d38:1 | 0.162 | 0.420 |
| PC 35:4 (2) | 0.157 | 0.403 |
| TAG 56:8 TAG 16:0-18:2-22:6 | 0.156 | 0.397 |
| TAG 48:4 B | 0.155 | 0.395 |
| PE p-36:2 or PE o-36:3 | 0.154 | 0.392 |
| PC 40:4 (2) | 0.153 | 0.388 |
| PC p-36:2/PC o-36:3 | 0.152 | 0.386 |
| PC 34:2 (2) | 0.149 | 0.374 |
| DAG 38:3 DAG 20:1-18:2 | 0.147 | 0.367 |
| PE 37:6 PE 15:0-22:6 | 0.145 | 0.358 |
| SM d42:2 B | 0.142 | 0.349 |
| LPE 20:1 | 0.142 | 0.349 |
| Cer d36:1 | 0.141 | 0.346 |
| Cer d38:1 | 0.141 | 0.345 |
| PE 39:6 PE 17:0-22:6 | 0.139 | 0.337 |
| CL 70:5 | 0.132 | 0.315 |
| LNAPS 36:4 LNAPS 16:0/n-20:4 | 0.130 | 0.307 |
| PC 40:6 B | 0.127 | 0.295 |
| PC 38:6 B | 0.125 | 0.287 |
| LPE 18:2 A | 0.124 | 0.284 |
| PE p-38:3 or PE o-38:4 | 0.123 | 0.281 |
| LPC 18:2 A | 0.122 | 0.279 |
| PC 34:4 | 0.122 | 0.279 |
| TAG 60:7 | 0.122 | 0.277 |
| PC o-34:0 | 0.121 | 0.275 |
| FA 22:6 (docosahexaenoic acid) | 0.119 | 0.268 |
| Ceramide d33:1 | 0.114 | 0.251 |
| FA 18:2 (linoleic acid) | 0.113 | 0.246 |
| LPE 22:6 | 0.110 | 0.237 |
| PC 34:3 | 0.109 | 0.234 |
| PE 34:2 (2) | 0.107 | 0.225 |
| LNAPS 40:6 LNAPS 18:0/n-22:6 | 0.105 | 0.219 |
| DG 36:4 B | 0.104 | 0.216 |
| DG 36:3 | 0.104 | 0.214 |
| PC 34:0 (2) | 0.101 | 0.204 |
| PI 40:8 PI 20:4-20:4 | 0.101 | 0.203 |
| FAHFA 32:0 FAHFA 16:0/16:0 | 0.098 | 0.194 |
| PI 38:5 | 0.097 | 0.190 |
| PC 33:2 | 0.097 | 0.188 |
| PE 33:2 PE 15:0-18:2 | 0.096 | 0.185 |
| SM d44:1 | 0.092 | 0.171 |
| SM d43:1 A | 0.088 | 0.159 |
| GlcCer d40:1 (2) | 0.087 | 0.156 |
| Cer-NS d42:1 Cer-NS d18:1/24:0 A | 0.086 | 0.152 |
| PC 36:5 A | 0.085 | 0.148 |
| FA 18:3 (linolenic acid) | 0.084 | 0.144 |
| DAG 38:4 DAG 18:2-20:2 | 0.083 | 0.141 |
| CL 72:6 CL 36:3-36:3 | 0.079 | 0.126 |
| Cer-NS d38:1 Cer-NS d18:1/20:0 | 0.078 | 0.122 |
| SM d41:2 B | 0.078 | 0.122 |
| PE 40:6 B | 0.077 | 0.119 |
| TAG 56:9 TAG 18:2-18:2-20:5 | 0.075 | 0.113 |
| SM d43:1 | 0.075 | 0.111 |
| LPC 18:2 | 0.072 | 0.103 |
| PC 36:5 A (2) | 0.066 | 0.080 |
| LNAPS 38:6 | 0.061 | 0.063 |
| GlcCer d41:1 | 0.059 | 0.055 |
| CE 20:5 | 0.057 | 0.049 |
| CL 70:4 CL 34:1-36:3 | 0.057 | 0.049 |
| Cer-NS d42:3 Cer-NS d18:1/24:2 | 0.055 | 0.041 |
| PC 38:6 | 0.050 | 0.023 |
| TAG 52:4 TAG 16:0-18:2-18:2 | 0.048 | 0.016 |
| PC 36:2 (2) | 0.047 | 0.012 |
| TAG 54:3 TAG 16:0-18:0-20:3 | 0.045 | 0.007 |
| SM d41:1 (2) | 0.045 | 0.007 |
| PI 38:4 | 0.041 | -0.008 |
| PI 36:4 | 0.038 | -0.020 |
| TAG 53:4 | 0.038 | -0.020 |
| BMP 44:12 BMP 22:6-22:6 | 0.037 | -0.021 |
| LPE 19:0 | 0.037 | -0.022 |
| PE 38:2 | 0.036 | -0.027 |
| SM d41:2B 2 | 0.034 | -0.034 |
| LPC 14:0 (2) | 0.033 | -0.038 |
| LPC 16:0 A | 0.033 | -0.038 |
| PC 37:2 | 0.031 | -0.044 |
| LPE 19:0 (2) | 0.029 | -0.050 |
| PE 34:3 PE 16:0-18:3 | 0.029 | -0.052 |
| TAG 52:5 TAG 16:0-16:0-20:5 | 0.023 | -0.072 |
| LPE 17:0 | 0.021 | -0.081 |
| PC 40:6 B (2) | 0.019 | -0.086 |
| PC 33:2 (2) | 0.018 | -0.088 |
| LPC 20:5 (2) | 0.017 | -0.093 |
| PC 34:4 (2) | 0.016 | -0.097 |
| LPE 16:0 B | 0.012 | -0.111 |
| TAG 50:5 | 0.012 | -0.112 |
| TAG 52:7 TAG 16:1-16:1-20:5 | 0.011 | -0.115 |
| TAG 56:6 TAG 18:1-18:1-20:4 | 0.010 | -0.117 |
| TAG 50:4 TAG 16:1-16:1-18:2 | 0.010 | -0.119 |
| LPC 22:6 | 0.006 | -0.134 |
| SM d42:1 (2) | 0.004 | -0.140 |
| FA 20:5 (eicosapentaenoic acid) | 0.003 | -0.143 |
| PE 38:6 (2) | 0.001 | -0.149 |
| LPE 22:6 (2) | -0.001 | -0.158 |
| TAG 54:3 TAG 16:0-18:1-20:2 | -0.003 | -0.163 |
| TAG 55:3 | -0.004 | -0.169 |
| LPC 22:6 2 | -0.005 | -0.170 |
| PC 37:2 (2) | -0.005 | -0.171 |
| LPE 18:2e | -0.005 | -0.173 |
| SM d43:1 B | -0.007 | -0.178 |
| PE p-34:2 or PE o-34:3 | -0.007 | -0.180 |
| Cer-NS d40:2 Cer-NS d18:1/22:1 | -0.007 | -0.180 |
| PC p-42:3 or PC o-42:4 | -0.008 | -0.181 |
| LPE 18:0 (2) | -0.009 | -0.184 |
| DAG 32:2 DAG 16:1-16:1 | -0.012 | -0.195 |
| TAG 56:3 TAG 18:1-18:1-20:1 | -0.012 | -0.195 |
| FA 22:2 | -0.012 | -0.197 |
| DAG 36:4e DAG 19:2e/17:2 | -0.013 | -0.199 |
| PC 34:3 C | -0.014 | -0.205 |
| FA 22:1 | -0.015 | -0.207 |
| SM d32:1 | -0.016 | -0.210 |
| Ceramide d40:2 | -0.017 | -0.215 |
| TAG 53:3 TAG 17:1-17:1-19:1 | -0.019 | -0.222 |
| PC 36:2 | -0.020 | -0.223 |
| PC 38:4 B | -0.023 | -0.237 |
| LNAPS 38:5 LNAPS 18:0/n-20:5 | -0.025 | -0.242 |
| Ceramide d40:1 | -0.026 | -0.245 |
| DG 36:1 | -0.027 | -0.248 |
| LPE 18:2 B | -0.031 | -0.263 |
| PC 37:4 (2) | -0.034 | -0.275 |
| PC 36:4 A | -0.037 | -0.283 |
| PC 38:1 | -0.040 | -0.294 |
| LPE 20:0 | -0.040 | -0.295 |
| Cer-NS d41:1 Cer-NS d18:1/23:0 B | -0.040 | -0.296 |
| LPC 18:2 B | -0.041 | -0.300 |
| PC p-34:2 or PC o-34:3 | -0.042 | -0.302 |
| PC 34:1 (2) | -0.043 | -0.307 |
| Cer-NS d40:1 Cer-NS d18:1/22:0 | -0.044 | -0.308 |
| PC p-34:2/PC o-34:3 | -0.044 | -0.309 |
| PE 40:8 A | -0.044 | -0.310 |
| TAG 51:3 | -0.044 | -0.311 |
| BMP 34:1 BMP 16:0-18:1 | -0.045 | -0.313 |
| GlcCer d42:1 | -0.047 | -0.321 |
| LPE 18:0 | -0.048 | -0.323 |
| TAG 52:2 TAG 16:0-18:1-18:1 | -0.048 | -0.325 |
| Ceramide d39:1 | -0.049 | -0.329 |
| TAG 54:2 | -0.050 | -0.329 |
| FA 20:4 (arachidonic acid) | -0.051 | -0.333 |
| PC p-36:1/PC o-36:2 A | -0.053 | -0.340 |
| Cer-NS d43:1 Cer-NS d18:1/25:0 | -0.054 | -0.346 |
| LPE 18:1 | -0.056 | -0.352 |
| LPE 22:5 | -0.057 | -0.357 |
| FA 21:1 | -0.059 | -0.361 |
| PC 37:6 | -0.059 | -0.362 |
| LPC 20:5 | -0.059 | -0.363 |
| LPC 18:3 | -0.060 | -0.365 |
| PC 32:2 (2) | -0.060 | -0.365 |
| TAG 53:2 | -0.060 | -0.366 |
| LPE 17:0 (2) | -0.061 | -0.368 |
| LPC 22:4 | -0.062 | -0.375 |
| LPC 18:0 B | -0.063 | -0.378 |
| PC 38:3 A | -0.064 | -0.379 |
| LPE 16:1e | -0.066 | -0.387 |
| FA 16:0 (palmitic acid) | -0.067 | -0.390 |
| FA 20:1 | -0.069 | -0.398 |
| LPE 16:0 | -0.069 | -0.400 |
| Cer-NS d42:3 Cer-NS d18:2/24:1 | -0.072 | -0.410 |
| PE 35:0 PE 17:0-18:0 | -0.074 | -0.415 |
| FA 24:6 | -0.075 | -0.419 |
| LPC 22:5 | -0.075 | -0.421 |
| FA 17:0 (margaric acid) | -0.076 | -0.423 |
| FA 22:3 | -0.077 | -0.428 |
| PC 40:8 (2) | -0.078 | -0.430 |
| PE p-36:5 or PE o-36:6 | -0.082 | -0.445 |
| FA 20:0 | -0.088 | -0.464 |
| LPC 16:0 B | -0.089 | -0.467 |
| FA 21:0 | -0.094 | -0.488 |
| PC 34:3 B | -0.095 | -0.489 |
| FA 24:5 | -0.099 | -0.503 |
| CL 74:9 | -0.101 | -0.511 |
| PC 40:6 A | -0.102 | -0.514 |
| LPC 14:0 | -0.102 | -0.514 |
| PC 33:0 | -0.104 | -0.520 |
| Cer-NS d43:2 Cer-NS d20:2/23:0 | -0.105 | -0.527 |
| LPE 18:2 | -0.107 | -0.532 |
| PC 35:2 | -0.108 | -0.536 |
| DG 36:6 | -0.110 | -0.545 |
| PC 32:2 | -0.113 | -0.555 |
| FA 30:1 | -0.113 | -0.555 |
| FA 20:0 (arachidic acid) | -0.114 | -0.558 |
| FA 32:1 | -0.115 | -0.560 |
| LPE 18:1e | -0.116 | -0.564 |
| SM d32:1 (2) | -0.117 | -0.566 |
| PC 34:1 | -0.117 | -0.568 |
| FA 17:2 | -0.117 | -0.569 |
| PG 40:8 PG 18:2-22:6 | -0.118 | -0.573 |
| DG 34:1 | -0.120 | -0.578 |
| PE 40:5 PE 18:0-22:5 | -0.120 | -0.579 |
| DAG 34:3e DAG 19:2e/15:1 | -0.120 | -0.580 |
| TAG 52:1 | -0.121 | -0.581 |
| LPC p-16:0 or LPC o-16:1 | -0.121 | -0.582 |
| LPE 16:0 A | -0.122 | -0.585 |
| PI 40:6 | -0.122 | -0.587 |
| PC 35:2 B | -0.123 | -0.588 |
| PC 40:5 A (2) | -0.123 | -0.589 |
| PC 18:0e | -0.124 | -0.591 |
| TAG 48:3 | -0.124 | -0.592 |
| FA 19:0 | -0.125 | -0.596 |
| Cer-NS d43:1 Cer-NS d18:1/25:0 (2) | -0.126 | -0.600 |
| Cer-NS d41:2 Cer-NS d26:2/15:0 | -0.134 | -0.627 |
| DG 34:3 | -0.134 | -0.629 |
| PC 38:6 A | -0.136 | -0.634 |
| LPC 18:0 | -0.137 | -0.639 |
| FA 15:0 (pentadecylic acid) | -0.143 | -0.660 |
| FA 20:3 (eicosatrienoic acid) | -0.143 | -0.661 |
| AC 18:0 | -0.144 | -0.663 |
| DG 36:2 | -0.145 | -0.665 |
| Ceramide d42:2 A | -0.145 | -0.666 |
| PC 40:6 A (2) | -0.146 | -0.670 |
| FA 34:1 | -0.146 | -0.672 |
| LPE 18:1e (2) | -0.147 | -0.674 |
| FA 23:0 | -0.147 | -0.674 |
| PE 38:5 PE 18:1-20:4 | -0.148 | -0.679 |
| LPC 20:1 | -0.150 | -0.683 |
| PC 38:5 A (2) | -0.151 | -0.689 |
| Cer-NS d41:2 Cer-NS d18:2/23:0 | -0.152 | -0.691 |
| Gal-Gal-Cer d18:1/16:0 or Lactosylceramide d18:1/16:0 | -0.154 | -0.699 |
| Ceramide d41:1 | -0.155 | -0.701 |
| FA 24:1 | -0.155 | -0.702 |
| Cer-NS d42:2 Cer-NS d18:1/24:1 | -0.157 | -0.711 |
| PC 36:1 | -0.158 | -0.712 |
| Ceramide d43:1 | -0.170 | -0.755 |
| FA 22:5 | -0.171 | -0.758 |
| LPE 20:2 | -0.171 | -0.760 |
| PC p-36:5/PC o-36:6 | -0.174 | -0.768 |
| PE 36:4 PE 18:2-18:2 | -0.176 | -0.776 |
| FA 18:1 (oleic acid) | -0.177 | -0.780 |
| Ceramide d42:2 B | -0.177 | -0.782 |
| FA 14:1 (physeteric acid) | -0.180 | -0.791 |
| LPC 16:0 | -0.180 | -0.792 |
| PC 36:4 A (2) | -0.180 | -0.793 |
| DAG 32:0 DAG 16:0-16:0 | -0.182 | -0.798 |
| FA 20:2 | -0.187 | -0.815 |
| LPC 22:5 (2) | -0.192 | -0.834 |
| Cer-NS d41:1 Cer-NS d18:1/23:0 A | -0.197 | -0.852 |
| Ceramide d42:1 | -0.199 | -0.858 |
| FA 15:1 B | -0.200 | -0.861 |
| Cer d40:1 | -0.200 | -0.862 |
| PE 36:3 A | -0.203 | -0.873 |
| PC 35:3 | -0.206 | -0.883 |
| LPC 18:0 A | -0.207 | -0.886 |
| LPE 18:1 (2) | -0.209 | -0.895 |
| FA 14:0 (myristic acid) | -0.212 | -0.903 |
| Cer d42:2 A | -0.212 | -0.903 |
| TAG 50:2 | -0.213 | -0.907 |
| PE 36:3 | -0.214 | -0.910 |
| LPC 19:0-SN1 | -0.216 | -0.919 |
| DAG 35:2 DAG 17:1-18:1 | -0.216 | -0.920 |
| PC 40:5 A | -0.217 | -0.921 |
| PC 36:1 (2) | -0.219 | -0.930 |
| PC 38:5 B (2) | -0.222 | -0.941 |
| FA 26:1 | -0.224 | -0.947 |
| LPC p-18:0 or LPC o-18:1 | -0.225 | -0.949 |
| LPE 20:2 (2) | -0.228 | -0.961 |
| PC 38:5 A | -0.228 | -0.961 |
| LPC o-16:0 | -0.229 | -0.966 |
| LPC 15:0 | -0.231 | -0.970 |
| FA 18:1 | -0.234 | -0.981 |
| PC 36:5 C | -0.234 | -0.982 |
| PC 37:5 | -0.235 | -0.985 |
| FA 15:1 A | -0.235 | -0.986 |
| Cer-NS d42:1 Cer-NS d18:1/24:0 B | -0.235 | -0.987 |
| Cer d41:1 | -0.243 | -1.015 |
| LPC 17:1 | -0.252 | -1.045 |
| CL 70:7 CL 34:3-36:4 | -0.253 | -1.048 |
| FA 20:2 (eicosadienoic acid) | -0.255 | -1.056 |
| Ceramide d32:1 | -0.257 | -1.065 |
| PE 40:8 PE 20:4-20:4 | -0.260 | -1.073 |
| PC 42:6 | -0.262 | -1.082 |
| DAG 32:1 DAG 14:0-18:1 | -0.266 | -1.096 |
| LPE 16:1 | -0.271 | -1.112 |
| LPC 14:0-SN1 | -0.271 | -1.114 |
| PC 36:6 | -0.272 | -1.117 |
| Cer d42:1 | -0.274 | -1.125 |
| LPC 20:2 | -0.286 | -1.166 |
| Cer d42:2 B | -0.288 | -1.172 |
| LPC 20:3 | -0.289 | -1.175 |
| PC 36:5 D | -0.292 | -1.187 |
| FAHFA 18:0 FAHFA 7:0/11:0 | -0.292 | -1.188 |
| PC 36:5 B | -0.295 | -1.199 |
| PE 34:1 | -0.297 | -1.206 |
| PC 35:1 (2) | -0.299 | -1.211 |
| PE 38:5 A | -0.303 | -1.226 |
| LPE 20:3 | -0.303 | -1.226 |
| PC 40:7 B | -0.310 | -1.252 |
| FA 19:1 | -0.314 | -1.264 |
| PE 38:3 PE 18:0-20:3 | -0.316 | -1.273 |
| PE 40:7 PE 18:1-22:6 | -0.319 | -1.282 |
| LPE 20:3 (2) | -0.321 | -1.289 |
| PC 38:5 B | -0.321 | -1.289 |
| LPC 16:1 | -0.323 | -1.297 |
| PE 40:8 B | -0.327 | -1.313 |
| TAG 48:0 | -0.330 | -1.322 |
| FA 17:1 | -0.334 | -1.337 |
| LPC 18:1 | -0.335 | -1.340 |
| PC 34:3 A | -0.336 | -1.343 |
| LPC 18:1 (2) | -0.338 | -1.352 |
| LPC 20:0 | -0.349 | -1.390 |
| LPC 20:3 (2) | -0.351 | -1.398 |
| PE 38:4 PE 18:1-20:3 | -0.363 | -1.440 |
| LPC 16:1 (2) | -0.368 | -1.455 |
| PC 35:1 | -0.369 | -1.461 |
| PC 32:1 (2) | -0.374 | -1.477 |
| PE 36:3 B | -0.375 | -1.482 |
| PC 40:5 B | -0.375 | -1.482 |
| PC 40:8 | -0.382 | -1.505 |
| PE 34:3 PE 16:1-18:2 | -0.382 | -1.505 |
| PC 39:6 | -0.384 | -1.515 |
| FA 20:3 (homo-gamma-linolenic acid) | -0.388 | -1.527 |
| PC 38:2 | -0.389 | -1.532 |
| PC 36:3 A (2) | -0.390 | -1.535 |
| PC 31:1 | -0.390 | -1.535 |
| FA 16:1 (palmitoleic acid) | -0.390 | -1.535 |
| PC 36:3 A | -0.391 | -1.538 |
| PC 33:1 | -0.391 | -1.539 |
| LPC 20:2 2 | -0.393 | -1.544 |
| CL 70:6 CL 34:3-36:3 | -0.395 | -1.554 |
| PC 38:3 | -0.401 | -1.572 |
| PC 33:1 (2) | -0.410 | -1.607 |
| PE 36:5 PE 16:1-20:4 | -0.411 | -1.608 |
| PE 40:7 A | -0.412 | -1.612 |
| PC 38:3 B | -0.420 | -1.640 |
| PC 38:2 (2) | -0.425 | -1.657 |
| PI 36:3 PI 16:0-20:3 | -0.425 | -1.658 |
| PC 40:7 | -0.428 | -1.668 |
| PC 36:3 B (2) | -0.430 | -1.676 |
| PC 32:1 | -0.435 | -1.695 |
| PE 36:5 A | -0.442 | -1.718 |
| PE 40:6 A | -0.447 | -1.737 |
| PC 35:2 A | -0.455 | -1.765 |
| PC 38:7 | -0.470 | -1.816 |
| PC 38:6 C | -0.471 | -1.823 |
| PC 40:7 A | -0.480 | -1.852 |
| PC 36:3 B | -0.482 | -1.860 |
| PI 40:7 | -0.504 | -1.939 |
| PE 38:7 PE 16:1-22:6 | -0.525 | -2.013 |

**Table S7**. PC5

| **Lipids** | **PC5 Loadings** | **PC5_Zscore** |
| --- | --- | --- |
| PE p-36:1 or PE o-36:2 | 0.668 | 2.953 |
| SM d36:1 (2) | 0.668 | 2.952 |
| PE p-40:6 or PE o-40:7 | 0.617 | 2.712 |
| SM d38:1 (2) | 0.612 | 2.687 |
| DAG 36:0 DAG 18:0-18:0 | 0.549 | 2.389 |
| PE p-38:3 or PE o-38:4 | 0.545 | 2.368 |
| Ceramide d38:1 | 0.543 | 2.360 |
| Ceramide d36:1 | 0.541 | 2.351 |
| Cer d36:1 | 0.527 | 2.282 |
| SM d36:2 (2) | 0.507 | 2.187 |
| PC 30:0 | 0.504 | 2.177 |
| Cer-NS d38:1 Cer-NS d18:1/20:0 | 0.493 | 2.125 |
| Cer d38:1 | 0.490 | 2.108 |
| SM d38:2 (2) | 0.488 | 2.099 |
| LPE 18:2e | 0.487 | 2.095 |
| PC o-32:0 | 0.463 | 1.980 |
| PC o-34:0 | 0.463 | 1.980 |
| SM d38:1 | 0.462 | 1.977 |
| Cer-NS d38:2 Cer-NS d18:2/20:0 | 0.456 | 1.950 |
| PC 32:0 (2) | 0.448 | 1.908 |
| PC p-38:6/PC o-38:7 | 0.445 | 1.897 |
| SM d36:1 | 0.439 | 1.867 |
| PE p-38:6 or PE o-38:7 | 0.426 | 1.808 |
| SM d36:0 | 0.418 | 1.769 |
| PC o-32:0 (2) | 0.418 | 1.768 |
| PC p-36:1/PC o-36:2 A | 0.411 | 1.737 |
| LPE 16:1e | 0.410 | 1.728 |
| PE p-36:5 or PE o-36:6 | 0.405 | 1.707 |
| SM d38:0 | 0.398 | 1.673 |
| Ceramide d40:1 | 0.384 | 1.609 |
| LPC p-16:0 or LPC o-16:1 | 0.376 | 1.571 |
| LNAPS 38:5 LNAPS 18:0/n-20:5 | 0.376 | 1.567 |
| LNAPS 40:5 LNAPS 18:0/n-22:5 | 0.361 | 1.499 |
| PE 38:4e PE 18:0e/20:4 | 0.360 | 1.495 |
| Ceramide d40:0 | 0.353 | 1.460 |
| PC p-34:1/PC o-34:2 | 0.350 | 1.448 |
| PC 31:0 | 0.347 | 1.432 |
| CL 70:6 CL 34:3-36:3 | 0.345 | 1.423 |
| PC 38:4 B | 0.341 | 1.405 |
| LNAPS 38:6 | 0.334 | 1.372 |
| Cer-NS d40:1 Cer-NS d18:1/22:0 | 0.333 | 1.365 |
| PC p-34:2/PC o-34:3 | 0.327 | 1.339 |
| SM d40:2 B (2) | 0.326 | 1.331 |
| LPE 18:1e (2) | 0.324 | 1.322 |
| PC 34:1 | 0.323 | 1.316 |
| PC 33:0 | 0.321 | 1.309 |
| Cer d40:1 | 0.319 | 1.299 |
| PC p-34:2 or PC o-34:3 | 0.314 | 1.278 |
| PC 40:4 | 0.311 | 1.263 |
| LPE 18:1e | 0.311 | 1.261 |
| PE p-38:4 or PE o-38:5 | 0.306 | 1.239 |
| PC p-36:5/PC o-36:6 | 0.301 | 1.214 |
| PC 32:1 | 0.301 | 1.214 |
| PC 36:1 (2) | 0.299 | 1.207 |
| PC p-32:0 or PC o-32:1 | 0.298 | 1.202 |
| Cer-NS d36:2 Cer-NS d20:1/16:1 | 0.295 | 1.185 |
| DAG 32:1 DAG 14:0-18:1 | 0.288 | 1.153 |
| Ceramide d40:2 | 0.285 | 1.137 |
| PC p-34:0 or PC o-34:1 | 0.280 | 1.116 |
| PC 40:4 (2) | 0.278 | 1.107 |
| LNAPS 36:2 LNAPS 17:2/n-19:0 | 0.277 | 1.100 |
| DG 36:1 | 0.273 | 1.081 |
| SM d44:1 | 0.266 | 1.050 |
| PE p-38:5 or PE o-38:6 | 0.265 | 1.046 |
| LPC o-16:0 | 0.265 | 1.043 |
| Cer-NS d42:1 Cer-NS d18:1/24:0 B | 0.260 | 1.020 |
| PC p-38:5/PC o-38:6 A | 0.259 | 1.014 |
| PE p-34:1 or PE o-34:2 | 0.258 | 1.010 |
| LNAPS 40:6 LNAPS 18:0/n-22:6 | 0.255 | 0.998 |
| PC p-34:1 or PC o-34:2 A | 0.255 | 0.997 |
| SM d32:1 | 0.254 | 0.992 |
| PC p-36:2 or PC o-36:3 | 0.252 | 0.983 |
| PC 35:1 | 0.250 | 0.971 |
| PC p-42:3 or PC o-42:4 | 0.249 | 0.970 |
| PC 40:5 B | 0.248 | 0.962 |
| SM d32:1 (2) | 0.246 | 0.954 |
| DAG 32:2 DAG 16:1-16:1 | 0.241 | 0.932 |
| PE p-36:4 or PE o-36:5 | 0.239 | 0.919 |
| TAG 52:1 | 0.237 | 0.910 |
| PC 31:1 | 0.235 | 0.904 |
| Cer-NS d43:2 Cer-NS d20:2/23:0 | 0.235 | 0.900 |
| SM d34:2 (2) | 0.234 | 0.898 |
| Ceramide d42:1 | 0.233 | 0.891 |
| PC p-32:0 or PC o-32:1 2 | 0.231 | 0.885 |
| PC p-38:5 or PC o-38:6 | 0.231 | 0.883 |
| PC 34:1 (2) | 0.230 | 0.879 |
| FA 23:0 | 0.229 | 0.872 |
| LNAPS 38:4 LNAPS 17:2/n-21:2 | 0.228 | 0.871 |
| CL 70:7 CL 34:3-36:4 | 0.227 | 0.864 |
| PC 32:1 (2) | 0.225 | 0.853 |
| PC 38:5 B | 0.225 | 0.852 |
| PC 36:3e | 0.222 | 0.843 |
| DG 34:1 | 0.220 | 0.829 |
| PC 33:1 | 0.216 | 0.810 |
| LPC p-18:0 or LPC o-18:1 | 0.214 | 0.803 |
| FA 24:5 | 0.214 | 0.803 |
| Cholesterol | 0.209 | 0.779 |
| GlcCer d42:2 | 0.206 | 0.765 |
| Ceramide d43:1 | 0.201 | 0.743 |
| Ceramide d39:1 | 0.201 | 0.739 |
| PE p-34:2 or PE o-34:3 | 0.201 | 0.739 |
| PC 34:0 (2) | 0.200 | 0.738 |
| Ceramide d42:2 A | 0.199 | 0.733 |
| SM d34:1 (2) | 0.199 | 0.731 |
| DG 34:3 | 0.195 | 0.714 |
| PC 38:5 B (2) | 0.195 | 0.713 |
| SM d42:2 A | 0.195 | 0.712 |
| TAG 48:3 | 0.194 | 0.707 |
| TAG 54:2 | 0.193 | 0.704 |
| PC 32:0 | 0.191 | 0.693 |
| TAG 48:0 | 0.191 | 0.692 |
| PE 40:8 B | 0.190 | 0.691 |
| PE 36:1 | 0.188 | 0.678 |
| PC 34:0 | 0.187 | 0.675 |
| PC p-38:3/PC o-38:4 B | 0.187 | 0.674 |
| BMP 44:12 BMP 22:6-22:6 | 0.185 | 0.667 |
| TAG 51:3 | 0.185 | 0.666 |
| PC 33:1 (2) | 0.184 | 0.660 |
| PC 36:5 B | 0.184 | 0.660 |
| PE 38:5 A | 0.180 | 0.643 |
| Ceramide d44:1 | 0.179 | 0.638 |
| LNAPS 36:4 LNAPS 16:0/n-20:4 | 0.178 | 0.632 |
| PC 38:1 | 0.177 | 0.629 |
| PC 38:3 B | 0.175 | 0.618 |
| Cer d42:1 | 0.174 | 0.615 |
| PC 38:3 | 0.173 | 0.610 |
| PC 36:3 B | 0.173 | 0.608 |
| PC 35:1 (2) | 0.171 | 0.599 |
| PC 36:1 | 0.170 | 0.596 |
| SM d34:0 | 0.170 | 0.596 |
| DAG 35:2 DAG 17:1-18:1 | 0.170 | 0.593 |
| SM d40:1 | 0.169 | 0.591 |
| Cer-NS d42:2 Cer-NS d18:1/24:1 | 0.168 | 0.584 |
| FA 20:3 (homo-gamma-linolenic acid) | 0.168 | 0.583 |
| Ceramide d34:0 | 0.167 | 0.580 |
| Ceramide d32:1 | 0.166 | 0.577 |
| TAG 53:3 TAG 17:1-17:1-19:1 | 0.165 | 0.573 |
| Cer-NS d41:1 Cer-NS d18:1/23:0 A | 0.165 | 0.571 |
| TAG 53:2 | 0.164 | 0.566 |
| FA 24:2 | 0.164 | 0.565 |
| TAG 50:4 TAG 16:1-16:1-18:2 | 0.159 | 0.542 |
| PC p-36:2/PC o-36:3 | 0.157 | 0.531 |
| PE p-40:7 or PE o-40:8 | 0.155 | 0.521 |
| TAG 56:3 TAG 18:1-18:1-20:1 | 0.150 | 0.501 |
| Cer d42:2 B | 0.150 | 0.500 |
| SM d40:1 (2) | 0.150 | 0.499 |
| PC 36:2 (2) | 0.147 | 0.483 |
| PE p-36:2 or PE o-36:3 | 0.146 | 0.481 |
| PC 34:3 B | 0.146 | 0.481 |
| DG 36:6 | 0.146 | 0.479 |
| Cer-NS d42:3 Cer-NS d18:1/24:2 | 0.145 | 0.478 |
| PC 42:6 | 0.145 | 0.475 |
| Cer d34:1 | 0.144 | 0.469 |
| Cer-NS d41:2 Cer-NS d18:2/23:0 | 0.143 | 0.465 |
| PC 38:6 C | 0.141 | 0.455 |
| PC 36:3 B (2) | 0.135 | 0.429 |
| Cer d42:2 A | 0.135 | 0.428 |
| TAG 54:3 TAG 16:0-18:1-20:2 | 0.135 | 0.427 |
| TAG 48:4 B | 0.134 | 0.424 |
| SM d40:2 B | 0.134 | 0.423 |
| PE p-40:4 or PE o-40:5 | 0.132 | 0.414 |
| PC 36:2 | 0.132 | 0.413 |
| PC 37:5 | 0.131 | 0.408 |
| SM d39:1 (2) | 0.130 | 0.407 |
| TAG 52:2 TAG 16:0-18:1-18:1 | 0.130 | 0.405 |
| DG 34:2 | 0.130 | 0.404 |
| TAG 50:2 | 0.130 | 0.403 |
| PC 36:5 D | 0.127 | 0.392 |
| FA 20:5 | 0.126 | 0.386 |
| FA 24:1 | 0.125 | 0.383 |
| Cer d41:1 | 0.124 | 0.377 |
| Ceramide d42:2 B | 0.123 | 0.373 |
| TAG 56:6 TAG 18:1-18:1-20:4 | 0.122 | 0.365 |
| PC 34:3 | 0.121 | 0.363 |
| SM d42:2 | 0.120 | 0.356 |
| TAG 54:3 TAG 16:0-18:0-20:3 | 0.119 | 0.352 |
| PE 34:3 PE 16:0-18:3 | 0.118 | 0.347 |
| PC 38:2 | 0.117 | 0.342 |
| PE 38:3 PE 18:0-20:3 | 0.116 | 0.341 |
| Ceramide d41:1 | 0.116 | 0.338 |
| PE 35:0 PE 17:0-18:0 | 0.116 | 0.337 |
| PC 38:2 (2) | 0.113 | 0.327 |
| SM d42:3 (2) | 0.110 | 0.312 |
| PE 36:5 A | 0.110 | 0.310 |
| TAG 55:3 | 0.109 | 0.307 |
| FA 16:1 (palmitoleic acid) | 0.108 | 0.302 |
| TAG 52:5 TAG 16:0-16:0-20:5 | 0.105 | 0.287 |
| CL 74:9 | 0.105 | 0.285 |
| PC 36:3 A (2) | 0.105 | 0.285 |
| SM d41:2 A (2) | 0.103 | 0.279 |
| LPC 16:1 (2) | 0.103 | 0.278 |
| DG 36:2 | 0.102 | 0.273 |
| PC 40:7 B | 0.101 | 0.269 |
| PC p-36:3 or PC o-36:4 2 | 0.100 | 0.264 |
| Cer-NS d40:2 Cer-NS d18:1/22:1 | 0.100 | 0.264 |
| PC 34:2 (2) | 0.100 | 0.263 |
| CL 72:6 CL 36:3-36:3 | 0.099 | 0.259 |
| PE 34:3 PE 16:1-18:2 | 0.099 | 0.256 |
| PE 40:6 A | 0.097 | 0.250 |
| FA 24:4 | 0.093 | 0.232 |
| Ceramide d34:1 | 0.093 | 0.232 |
| Cer-NS d41:2 Cer-NS d26:2/15:0 | 0.093 | 0.230 |
| PC 34:3 A | 0.091 | 0.219 |
| TAG 56:8 TAG 16:0-18:2-22:6 | 0.091 | 0.219 |
| Cer-NS d42:3 Cer-NS d18:2/24:1 | 0.090 | 0.217 |
| SM d42:2 B | 0.090 | 0.214 |
| ACar 18:2 | 0.088 | 0.204 |
| GlcCer d40:1 (2) | 0.087 | 0.201 |
| GlcCer d42:2 (2) | 0.087 | 0.201 |
| FA 21:0 | 0.086 | 0.198 |
| PE 36:5 PE 16:1-20:4 | 0.085 | 0.192 |
| LPC 20:5 | 0.081 | 0.173 |
| PE 36:3 B | 0.081 | 0.172 |
| TAG 52:7 TAG 16:1-16:1-20:5 | 0.081 | 0.171 |
| Cer-NS d34:1 Cer-NS d18:1/16:0 | 0.078 | 0.159 |
| SM d43:1 B | 0.078 | 0.158 |
| SM d34:1 | 0.077 | 0.154 |
| PC 35:3 | 0.074 | 0.139 |
| PC p-38:3 or PC o-38:4 | 0.073 | 0.136 |
| GlcCer d42:1 | 0.070 | 0.123 |
| GlcCer d41:1 | 0.069 | 0.114 |
| PC 40:6 B | 0.067 | 0.105 |
| FA 20:0 | 0.066 | 0.102 |
| LPC 18:0 A | 0.065 | 0.099 |
| FA 22:1 | 0.062 | 0.081 |
| Ceramide d34:2 | 0.059 | 0.069 |
| DAG 38:4 DAG 18:2-20:2 | 0.059 | 0.069 |
| PC 40:5 A | 0.059 | 0.068 |
| PC 40:6 B (2) | 0.058 | 0.066 |
| PC p-38:4/PC o-38:5 A | 0.057 | 0.060 |
| PI 36:3 PI 16:0-20:3 | 0.057 | 0.059 |
| PC 35:2 A | 0.057 | 0.059 |
| PC 36:3 A | 0.057 | 0.058 |
| DAG 32:0 DAG 16:0-16:0 | 0.056 | 0.055 |
| DG 38:5 | 0.055 | 0.049 |
| SM d42:3 | 0.054 | 0.045 |
| ACar 20:4 | 0.054 | 0.044 |
| LPC 16:1 | 0.053 | 0.042 |
| PC 38:6 B | 0.053 | 0.040 |
| PC 40:7 A | 0.052 | 0.038 |
| SM d42:0 | 0.050 | 0.028 |
| TAG 52:4 TAG 16:0-18:2-18:2 | 0.049 | 0.022 |
| FA 17:1 | 0.049 | 0.021 |
| PC 40:5 A (2) | 0.048 | 0.018 |
| PE 34:1 | 0.048 | 0.015 |
| TAG 50:5 | 0.045 | 0.001 |
| PE 40:5 PE 18:0-22:5 | 0.043 | -0.005 |
| FA 20:3 (eicosatrienoic acid) | 0.040 | -0.020 |
| FA 14:0 (myristic acid) | 0.040 | -0.022 |
| PE 38:7 PE 16:1-22:6 | 0.038 | -0.030 |
| SM d34:2 | 0.038 | -0.031 |
| TAG 56:9 TAG 18:2-18:2-20:5 | 0.037 | -0.036 |
| PC 38:3 A | 0.036 | -0.039 |
| PC 38:7 | 0.035 | -0.044 |
| FA 14:1 (physeteric acid) | 0.034 | -0.048 |
| PE 34:2 (2) | 0.033 | -0.053 |
| FA 12:0 (lauric acid) | 0.033 | -0.053 |
| SM d39:1 | 0.032 | -0.057 |
| FAHFA 18:0 FAHFA 7:0/11:0 | 0.032 | -0.059 |
| GlcCer d40:1 | 0.031 | -0.062 |
| SM d34:0 (2) | 0.031 | -0.064 |
| BMP 34:1 BMP 16:0-18:1 | 0.029 | -0.072 |
| DG 36:3 | 0.029 | -0.074 |
| PE 38:2 | 0.026 | -0.089 |
| DAG 38:3 DAG 20:1-18:2 | 0.025 | -0.091 |
| TAG 54:6 | 0.025 | -0.091 |
| Gal-Gal-Cer d18:1/16:0 or Lactosylceramide d18:1/16:0 | 0.025 | -0.092 |
| SM d44:2 | 0.025 | -0.092 |
| FA 22:3 | 0.025 | -0.093 |
| CE 20:5 | 0.025 | -0.093 |
| FA 20:2 (eicosadienoic acid) | 0.024 | -0.095 |
| SM d41:2B 2 | 0.024 | -0.097 |
| PC 39:6 | 0.023 | -0.101 |
| PE 40:7 PE 18:1-22:6 | 0.022 | -0.107 |
| FA 24:6 | 0.020 | -0.115 |
| PG 40:8 PG 18:2-22:6 | 0.020 | -0.117 |
| PE 36:3 | 0.020 | -0.118 |
| PC p-36:3 or PC o-36:4 | 0.018 | -0.125 |
| SM d40:0 | 0.015 | -0.141 |
| PC p-36:4/PC o-36:5 | 0.014 | -0.142 |
| FA 18:1 | 0.014 | -0.144 |
| PI 40:6 | 0.012 | -0.152 |
| SM d42:1 | 0.012 | -0.154 |
| PC p-38:4 or PC o-38:5 A | 0.010 | -0.165 |
| Ceramide d33:1 | 0.008 | -0.173 |
| PE 38:4 PE 18:1-20:3 | 0.008 | -0.173 |
| PC 36:5 C | 0.007 | -0.175 |
| PE 40:7 A | 0.006 | -0.184 |
| LPC 18:1 | 0.004 | -0.189 |
| SM d41:2 A | 0.004 | -0.190 |
| PC 36:6 | 0.004 | -0.193 |
| PC 32:2 (2) | 0.002 | -0.201 |
| FA 20:5 (eicosapentaenoic acid) | 0.001 | -0.205 |
| CL 70:5 | 0.000 | -0.208 |
| LPC 20:2 2 | -0.002 | -0.221 |
| PI 40:7 | -0.003 | -0.225 |
| SM d41:2 B | -0.006 | -0.238 |
| TAG 53:4 | -0.006 | -0.239 |
| PC 34:2 | -0.008 | -0.246 |
| PC 38:6 | -0.008 | -0.246 |
| PC 35:2 B | -0.011 | -0.261 |
| FA 15:1 A | -0.012 | -0.266 |
| PC 40:7 | -0.012 | -0.268 |
| LPE 16:1 | -0.012 | -0.268 |
| PC 38:5 A (2) | -0.013 | -0.271 |
| FA 22:5 | -0.013 | -0.273 |
| DG 38:6 | -0.014 | -0.278 |
| DAG 40:6 DAG 18:2-22:4 | -0.015 | -0.282 |
| SM d42:1 (2) | -0.016 | -0.288 |
| PC 40:8 | -0.020 | -0.307 |
| FA 18:1 (oleic acid) | -0.020 | -0.307 |
| FA 19:0 | -0.021 | -0.307 |
| SM d40:2 A (2) | -0.027 | -0.340 |
| CL 70:4 CL 34:1-36:3 | -0.029 | -0.347 |
| PE 36:3 A | -0.029 | -0.347 |
| LPC 20:3 (2) | -0.032 | -0.362 |
| LPC 20:3 | -0.033 | -0.365 |
| SM d41:1 (2) | -0.033 | -0.365 |
| PC 32:2 | -0.033 | -0.366 |
| FA 22:6 (docosahexaenoic acid) | -0.033 | -0.369 |
| FA 19:1 | -0.034 | -0.369 |
| CholesterolSulfate | -0.035 | -0.375 |
| LPC 18:1 (2) | -0.037 | -0.383 |
| PC 35:2 | -0.046 | -0.428 |
| Cer-NS d43:1 Cer-NS d18:1/25:0 | -0.049 | -0.443 |
| PC 34:4 (2) | -0.049 | -0.444 |
| PC 33:2 (2) | -0.053 | -0.460 |
| FA 16:0 (palmitic acid) | -0.054 | -0.463 |
| LPE 20:3 (2) | -0.054 | -0.465 |
| Cer-NS d42:1 Cer-NS d18:1/24:0 A | -0.054 | -0.466 |
| FA 20:0 (arachidic acid) | -0.054 | -0.467 |
| FA 22:2 | -0.055 | -0.469 |
| PC 37:2 (2) | -0.055 | -0.469 |
| FA 20:2 | -0.057 | -0.480 |
| FA 21:1 | -0.057 | -0.482 |
| DG 36:4 A | -0.059 | -0.489 |
| FA 15:0 (pentadecylic acid) | -0.059 | -0.492 |
| PE 40:4 PE 18:0-22:4 | -0.060 | -0.493 |
| Cer-NS d41:1 Cer-NS d18:1/23:0 B | -0.061 | -0.497 |
| LPE 20:3 | -0.063 | -0.507 |
| FA 17:2 | -0.063 | -0.510 |
| PC 38:5 A | -0.064 | -0.514 |
| LPC 18:0 | -0.066 | -0.523 |
| TAG 60:7 | -0.067 | -0.529 |
| LPC 20:2 | -0.067 | -0.529 |
| PC 34:3 C | -0.067 | -0.530 |
| LPC 17:1 | -0.068 | -0.531 |
| TAG 60:12 TAG 20:4-20:4-20:4 | -0.069 | -0.537 |
| DG 36:4 B | -0.069 | -0.537 |
| PE 38:5 PE 18:1-20:4 | -0.069 | -0.538 |
| FA 20:1 | -0.070 | -0.539 |
| FA 26:1 | -0.072 | -0.551 |
| LPC 14:0-SN1 | -0.074 | -0.560 |
| SM d33:1 (2) | -0.075 | -0.566 |
| SM d33:1 | -0.077 | -0.574 |
| Cer-NS d43:1 Cer-NS d18:1/25:0 (2) | -0.080 | -0.590 |
| PI 38:5 | -0.081 | -0.592 |
| PC 36:4 A (2) | -0.082 | -0.600 |
| PC 40:6 A (2) | -0.089 | -0.632 |
| PC 18:0e | -0.093 | -0.651 |
| AC 18:0 | -0.094 | -0.653 |
| FA 20:4 (arachidonic acid) | -0.094 | -0.653 |
| LPC 18:0 B | -0.094 | -0.656 |
| FA 17:0 (margaric acid) | -0.095 | -0.661 |
| PC 37:2 | -0.096 | -0.664 |
| PE 36:2 (2) | -0.101 | -0.687 |
| LPE 18:0 | -0.106 | -0.711 |
| PC 36:4 A | -0.107 | -0.717 |
| DAG 36:4e DAG 19:2e/17:2 | -0.111 | -0.738 |
| PE 40:8 PE 20:4-20:4 | -0.112 | -0.742 |
| LPC 20:0 | -0.113 | -0.746 |
| PC 33:2 | -0.114 | -0.749 |
| LPC 22:6 2 | -0.114 | -0.751 |
| PC p-38:4/PC o-38:5 B | -0.117 | -0.763 |
| PC 38:6 A | -0.118 | -0.770 |
| LPE 20:0 | -0.120 | -0.777 |
| LPC 14:0 | -0.120 | -0.779 |
| Cer-NS d44:2 Cer-NS d18:1/26:1 | -0.121 | -0.782 |
| CL 72:7 CL 18:1-18:2-18:2-18:2 | -0.124 | -0.796 |
| SM d43:1 A | -0.124 | -0.796 |
| LPC 22:6 | -0.125 | -0.802 |
| PC 38:4 C | -0.126 | -0.806 |
| LPC 20:5 (2) | -0.126 | -0.808 |
| PE 38:6 | -0.129 | -0.820 |
| FA 30:1 | -0.130 | -0.827 |
| PC 37:4 (2) | -0.132 | -0.834 |
| PI 36:4 | -0.138 | -0.865 |
| PC 37:6 | -0.142 | -0.883 |
| PC 36:5 A (2) | -0.144 | -0.890 |
| DG 36:5 | -0.144 | -0.892 |
| LPC 20:1 | -0.145 | -0.894 |
| FA 18:2 (linoleic acid) | -0.145 | -0.895 |
| FA 15:1 B | -0.146 | -0.900 |
| LPE 22:5 | -0.147 | -0.904 |
| LPC 15:0 | -0.150 | -0.919 |
| PE 36:4 PE 18:2-18:2 | -0.152 | -0.928 |
| PE 36:2 | -0.153 | -0.935 |
| LPE 18:1 (2) | -0.153 | -0.937 |
| PC 36:4 C (2) | -0.161 | -0.973 |
| LPC 18:2 | -0.162 | -0.976 |
| LPC 18:2 A | -0.163 | -0.982 |
| SM d40:2 A | -0.164 | -0.986 |
| LPC 22:5 (2) | -0.165 | -0.991 |
| PE 38:4 B | -0.166 | -0.994 |
| PE 34:2 | -0.168 | -1.006 |
| LPC 18:2 B | -0.170 | -1.013 |
| PC 34:4 | -0.170 | -1.017 |
| LPC 16:0 | -0.171 | -1.020 |
| PC p-38:4 or PC o-38:5 B | -0.172 | -1.026 |
| LPC 18:3 | -0.173 | -1.027 |
| CE 22:6 | -0.174 | -1.034 |
| LPC 22:5 | -0.176 | -1.044 |
| LPC 22:4 | -0.176 | -1.045 |
| LPE 18:0 (2) | -0.177 | -1.047 |
| PE 33:2 PE 15:0-18:2 | -0.179 | -1.056 |
| LPC 16:0 B | -0.185 | -1.086 |
| LPE 18:1 | -0.186 | -1.090 |
| PE 39:6 PE 17:0-22:6 | -0.191 | -1.114 |
| PC p-40:3/PC o-40:4 | -0.194 | -1.130 |
| SM d43:1 | -0.194 | -1.130 |
| PC 40:6 A | -0.195 | -1.132 |
| CE 20:4 | -0.197 | -1.144 |
| FAHFA 32:0 FAHFA 16:0/16:0 | -0.198 | -1.147 |
| PC 36:5 A | -0.199 | -1.150 |
| PC 35:4 (2) | -0.201 | -1.161 |
| CL 74:8 CL 18:1-18:2-18:2-20:3 | -0.202 | -1.165 |
| LPE 19:0 (2) | -0.203 | -1.170 |
| PE 36:4 | -0.208 | -1.194 |
| LPE 20:2 (2) | -0.210 | -1.205 |
| PE 40:7 B | -0.211 | -1.210 |
| LPC 19:0-SN1 | -0.215 | -1.230 |
| PC 40:8 (2) | -0.217 | -1.237 |
| LPC 14:0 (2) | -0.218 | -1.244 |
| PE 38:4 A | -0.220 | -1.252 |
| FA 18:3 (linolenic acid) | -0.226 | -1.278 |
| LPE 20:2 | -0.228 | -1.288 |
| LPE 18:2 A | -0.234 | -1.319 |
| LPC 20:4 | -0.239 | -1.339 |
| PC 36:4 B | -0.240 | -1.344 |
| LPE 16:0 A | -0.241 | -1.351 |
| PC 38:4 A | -0.244 | -1.367 |
| FA 32:1 | -0.245 | -1.372 |
| LPE 22:6 (2) | -0.246 | -1.375 |
| PI 40:8 PI 20:4-20:4 | -0.250 | -1.395 |
| DAG 34:3e DAG 19:2e/15:1 | -0.250 | -1.395 |
| LPE 18:2 | -0.252 | -1.401 |
| PG 34:2 PG 16:0-18:2 | -0.256 | -1.424 |
| PE 38:6 (2) | -0.262 | -1.449 |
| LPE 22:6 | -0.263 | -1.453 |
| LPE 19:0 | -0.263 | -1.456 |
| CE 18:3 | -0.264 | -1.458 |
| LPE 16:0 | -0.270 | -1.488 |
| SMd30:1 | -0.275 | -1.512 |
| LPE 18:2 B | -0.278 | -1.525 |
| FA 34:1 | -0.278 | -1.525 |
| LPE 16:0 B | -0.278 | -1.525 |
| LPC 16:0 A | -0.282 | -1.547 |
| PE 40:6 B | -0.283 | -1.548 |
| PE 37:6 PE 15:0-22:6 | -0.289 | -1.577 |
| LPE 20:4 | -0.290 | -1.584 |
| PC 35:4 | -0.291 | -1.588 |
| PE 35:2 PE 17:0-18:2 | -0.297 | -1.615 |
| CL 72:8 CL 18:2-18:2-18:2-18:2 | -0.300 | -1.632 |
| CL 68:2 | -0.306 | -1.659 |
| CE 18:2 | -0.306 | -1.661 |
| LPE 20:4 A | -0.310 | -1.677 |
| PE 38:7 | -0.311 | -1.683 |
| PI 38:4 | -0.312 | -1.687 |
| LPE 17:0 | -0.329 | -1.768 |
| CE 18:1 | -0.334 | -1.793 |
| FA 16:3 | -0.335 | -1.797 |
| LPE 20:1 | -0.338 | -1.808 |
| PC 39:4 | -0.338 | -1.809 |
| LPE 20:4 B | -0.344 | -1.839 |
| PE 39:4 | -0.346 | -1.847 |
| LPE 17:0 (2) | -0.357 | -1.900 |
| FA 16:2 | -0.358 | -1.905 |
| PC 37:4 | -0.367 | -1.947 |
| PC 36:4 C | -0.369 | -1.958 |
| PE 38:5 B | -0.372 | -1.969 |
| PG 36:3 PG 18:1-18:2 | -0.374 | -1.981 |
| PE 37:4 PE 17:0-20:4 | -0.377 | -1.996 |
| FA 18:4 | -0.383 | -2.023 |
| PE 37:4 | -0.385 | -2.034 |
| PG 36:4 PG 18:2-18:2 | -0.393 | -2.071 |
| PE 40:9 | -0.407 | -2.137 |
| PE 36:5 B | -0.464 | -2.408 |
| PE 40:8 A | -0.539 | -2.760 |

**Figure S1.** **Study design.**

**
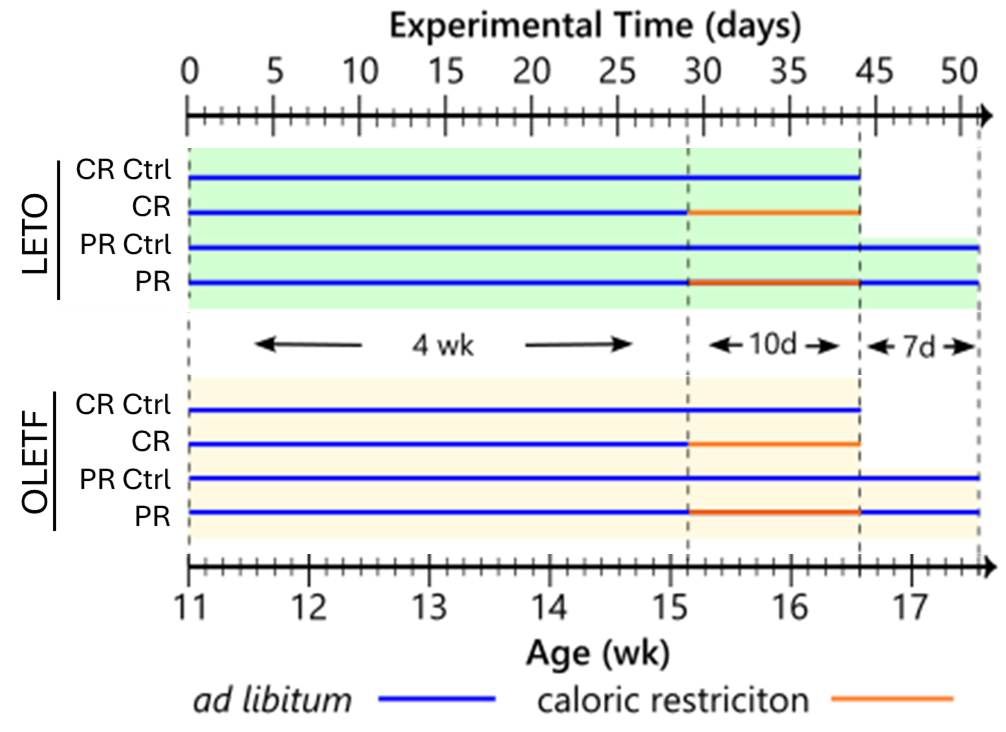
**

**Figure S2. PCA for each comparison.** Principal component analysis (PCA) score plots of principal components (x-axis) and (y-axis) for (A) LETO CR Ctrl vs OLETF CR Ctrl, (B) LETO PR Ctrl vs OLETF PR Ctrl, (C) LETO CR vs OLETF CR, (D) LETO PR vs OLETF PR, (E) LETO CR Ctrl vs LETO CR, (F) LETO PR Ctrl vs LETO PR, (G) OLETF CR Ctrl vs OLETF CR, (H) OLETF PR Ctrl vs OLETF PR (I) LETO CR vs LETO PR, and (J) OLETF CR vs OLETF PR.


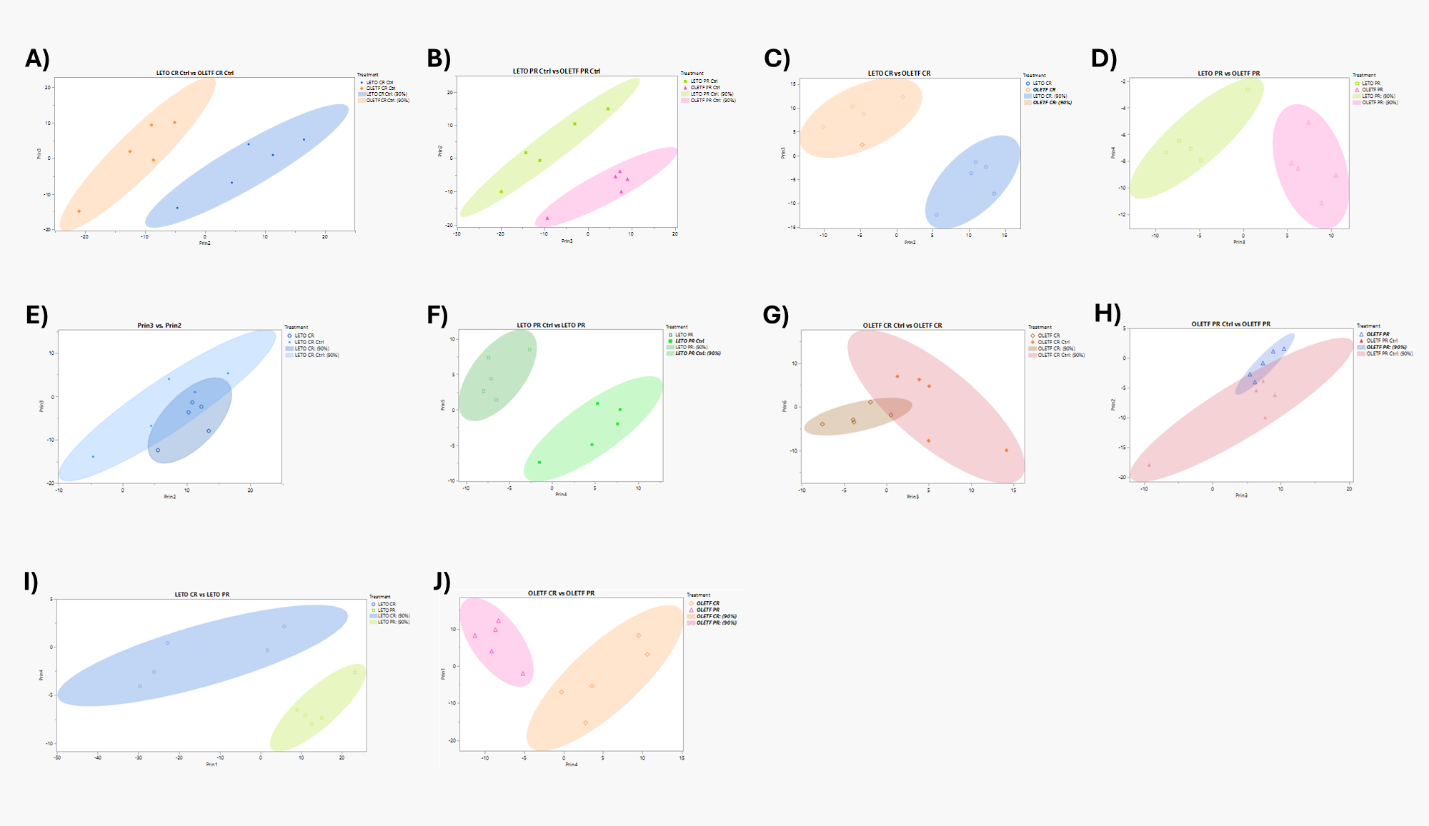


**Figure S3.** **Insulin AUC and WAT mass had the most lipid category correlation during PR.** Heat maps of Pearson’s correlation **(A)** ρ values for glucose AUC, insulin AUC and insulin resistance index (IRI) AUC, body mass (BM) change, and white adipose tissue (WAT) mass vs lipid classification per group and **(B)** p-values. The animal group names were concise to the following abbreviations: LCRC = LETO CR Ctrl
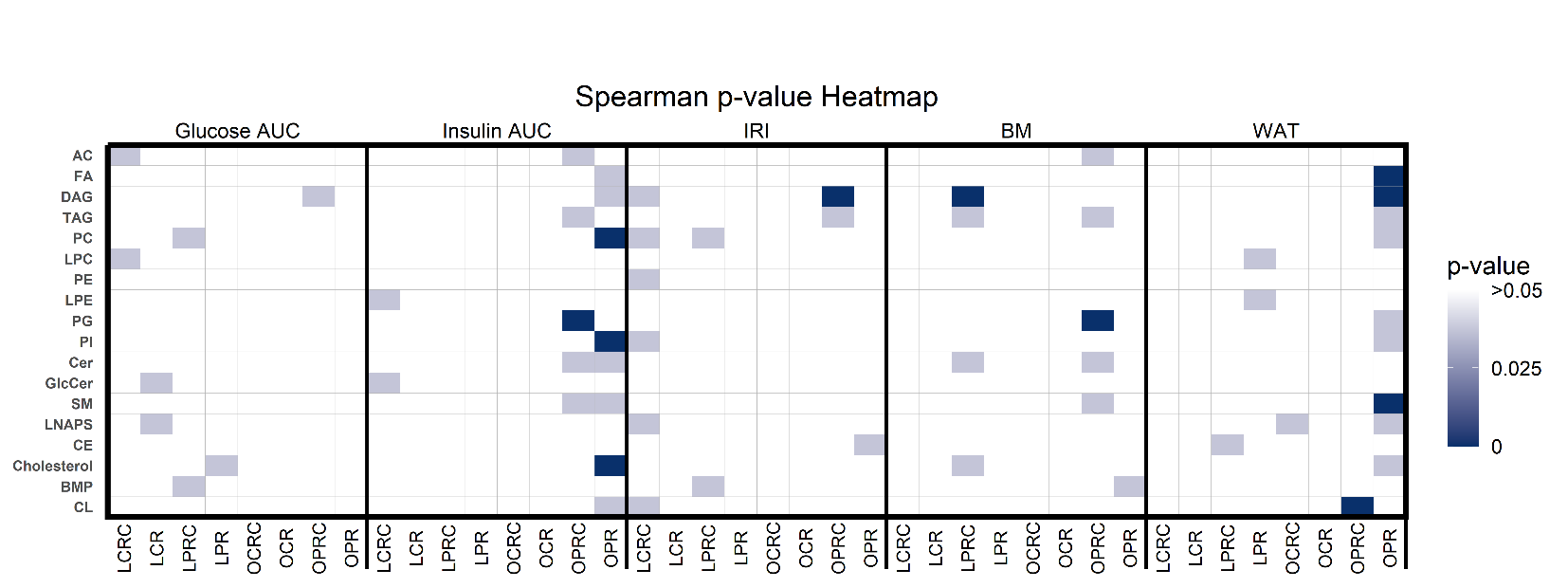
**
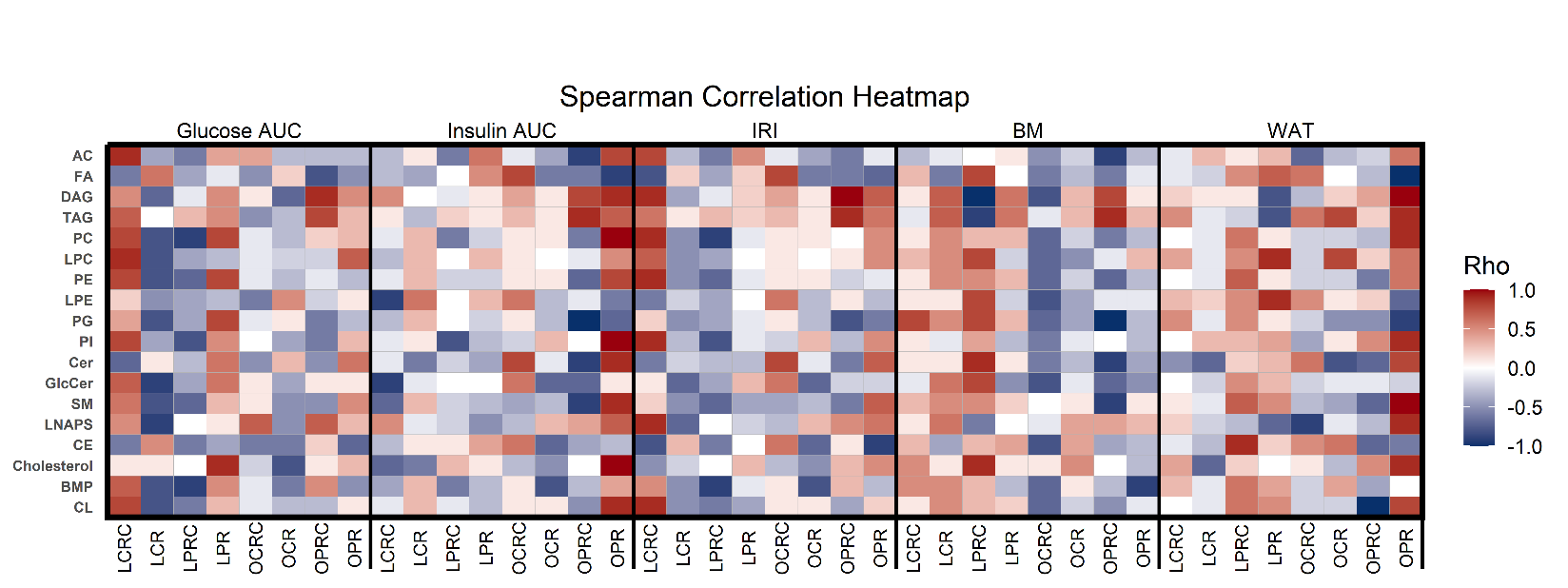
**, LCR = LETO CR, LPRC = LETO PR CTRL, LPR = LETO PR, OCRC = OLETF CR Ctrl, OCR = OLETF CR, OPRC = OLETF PR Ctrl, and OPR = OLETF PR Ctrl.

**B)**

**A)**
