# Supplementary material for: Partial body mass regain attenuates lipid utilization and alters the hepatic lipidome linked to HDL dysfunction during metabolic syndrome in male rats
Source: Physiol Rep. 2026 Jun 16;14(12):e70865. doi: 10.14814/phy2.70865 (PMC13273030; doi:10.14814/phy2.70865)
Supplement: Supplementary file 1 — Data S1. [file PHY2-14-e70865-s001.docx]

March 17th, 2026

Josephine C. Adams, Ph.D.

Editor-in-Chief

Physiological Reports

**Title:** Partial Body Mass Regain Attenuates Lipid Utilization and Alters the Hepatic Lipidome Linked to HDL Dysfunction during Metabolic Syndrome in Male Rats

**Authors:** Eira E. Jardines, M.S., Manuel A. Cornejo, Ph.D, John W. Newman, Ph.D., Oliver, Ph.D., Akira Nishiyama, M.D, Ph.D., and Rudy M. Ortiz, Ph.D.

Dear Dr. Adams,

On behalf of my co-authors, I am pleased to resubmit our revised original research manuscript for further consideration in *Physiological Reports.* I would like to thank you and the two reviewers for your continued investment of time to evaluate our manuscript. We addressed each of Reviewer #1’s four comments and highlight that we have satisfied Reviewer #2’s concerns as they did not provide any further comments.

In doing so, we have solidified the foundation regarding the importance of an acute and severe caloric restriction diet. More specifically we were able to clarify and justify the utilization of an acute and severe caloric restriction by demonstrating that this is an approach used in several studies of individuals with obesity as well as showing the negative impact body mass regain has on MAFLD in humans. Additionally, as mentioned by Reviewer #1, we have explicitly stated the relevance of the heatmaps and volcano plots due to them being crucial data to substantiate one of our main findings regarding LPC and lipid utilization disruption. Lastly, we reworded the caloric restriction findings to not distract from the main finding but instead to emphasize the detriments of body mass regain.

We remain excited for the opportunity to contribute to the journal’s collection of influential research in physiology and metabolism.

Thank you for your continued consideration.

Sincerely,

Eira E. Jardines M.S.

School of Natural Sciences

University of California, Merced
